# Exploring the Monoterpene Indole Alkaloid Scaffold for Reversing P-Glycoprotein-Mediated Multidrug Resistance in Cancer

**DOI:** 10.3390/ph14090862

**Published:** 2021-08-28

**Authors:** David S. P. Cardoso, Nikoletta Szemerédi, Gabriella Spengler, Silva Mulhovo, Daniel J. V. A. dos Santos, Maria-José U. Ferreira

**Affiliations:** 1 Research Institute for Medicines (iMed.ULisboa), Faculty of Pharmacy, Universidade de Lisboa, Av. Prof. Gama Pinto, 1649-003 Lisbon, Portugal; davidpcardoso@ff.ulisboa.pt (D.S.P.C.); ddsantos@fc.up.pt (D.J.V.A.d.S.); 2 Department of Medical Microbiology, Albert Szent-Györgyi Health Center, Faculty of Medicine, University of Szeged, Semmelweis utca 6, 6725 Szeged, Hungary; szemeredi.nikoletta@med.u-szeged.hu (N.S.); spengler.gabriella@med.u-szeged.hu (G.S.); 3 Centro de Estudos Moçambicanos e de Etnociências (CEMEC), Faculdade de Ciências Naturais e Matemática, Universidade Pedagógica Campus de Lhanguene, Av. De Moçambique, Maputo 21402161, Mozambique; smulhovo@hotmail.com; 4 LAQV@REQUIMTE/Department of Chemistry and Biochemistry, Faculty of Sciences, University of Porto, Rua do Campo Alegre, 4169-007 Porto, Portugal

**Keywords:** *Tabernaemontana elegans*, indole alkaloids, multidrug resistance, P-glycoprotein, ATPase activity, molecular docking, QSAR models

## Abstract

Dregamine (**1**), a major monoterpene indole alkaloid isolated from *Tabernaemontana elegans,* was submitted to chemical transformation of the ketone function, yielding 19 azines (**3**–**21**) and 11 semicarbazones (**22**–**32**) bearing aliphatic or aromatic substituents. Their structures were assigned mainly by 1D and 2D NMR (COSY, HMQC, and HMBC) experiments. Compounds **3**–**32** were evaluated as multidrug resistance (MDR) reversers through functional and chemosensitivity assays in a human *ABCB1*-transfected mouse T-lymphoma cell model, overexpressing P-glycoprotein. A significant increase of P-gp inhibitory activity was observed for most derivatives, mainly those containing azine moieties with aromatic substituents. Compounds with trimethoxyphenyl (**17**) or naphthyl motifs (**18**, **19**) were among the most active, exhibiting strong inhibition at 0.2 µM. Moreover, most of the derivatives showed selective antiproliferative effects toward resistant cells, having a collateral sensitivity effect. In drug combination assays, all compounds showed to interact synergistically with doxorubicin. Selected compounds (**12**, **17**, **18**, **20,** and **29**) were evaluated in the ATPase activity assay, in which all compounds but **12** behaved as inhibitors. To gather further insights on drug–receptor interactions, in silico studies were also addressed. A QSAR model allowed us to deduce that compounds bearing bulky and lipophilic substituents were stronger P-gp inhibitors.

## 1. Introduction

One of the main concerns regarding chemotherapy failure in cancer and infectious diseases is multidrug resistance (MDR). This complex phenomenon can be classified as intrinsic or acquired resistance. The former comprises all natural features of an organism or cell that makes them resistant to a certain drug, whereas acquired MDR involves a decrease in the susceptibility to a drug, generally due to a certain genetic modification [1,2]. In cancer cells, one well-known mechanism of acquired resistance results from the overexpression of ATP binding cassette (ABC) transporters, which extrude anticancer drugs using ATP, thus decreasing the intracellular drug concentration below the therapeutic window [3,4]. One of the main ABC transporters is the P-glycoprotein (P-gp/ABCB1) that is able to efflux a wide variety of chemically unrelated compounds. Several attempts have been made to develop effective inhibitors of this transporter. However, none of them have passed clinical trials, mainly due to their considerable toxicity or low in vivo efficacy [5,6]. The lack of detailed and reliable structural information of human P-gp, at a molecular level, has also been considered another hurdle in this field. Indeed, the first crystallographic structure of a mammalian P-gp (murine) only appeared in 2009, reported by Aller et al. [7]. Although the sequence identity to human P-gp is 87%, the first human P-gp structures were only reported in the last 3 years [8,9]. Owing to a large drug-binding pocket containing multiple drug-binding sites (DBSs), the substrate polyspecificity is huge [10]; thus, the search for novel molecules to act as MDR reversal agents that could inhibit the P-gp efflux mechanism and circumvent the drug promiscuity and poly-specific binding nature of P-gp has become emergent.

A different anti-MDR approach, known as collateral sensitivity (CS), focuses on searching compounds that are selectively cytotoxic against MDR cells over the parental ones, named CS agents [11]. In fact, MDR cancer cells overexpressing ABC transporters can be, at the same time, hypersensitive to certain agents. The mechanism behind this concept is not completely explained, although potent and highly selective CS agents have been recognized to have specific properties, namely, the ability to produce reactive oxygen species (ROS); to efflux endogenous substrates of a vital molecule; to take advantage of cells sensitivity, overexpressing ABC-transporters; to modify energy levels; or to alter the biophysical membrane properties [11]. Therefore, CS represents a novel strategy for avoiding ABC-transporters mediated MDR during chemotherapy or re-sensitizing MDR cancer cells and consequently reestablishing drug effectiveness.

Aiming at finding effective compounds for reversing MDR, our group has evaluated several plant-derived compounds with different scaffolds as effective ABC-transporter inhibitors, e.g., [12,13,14], with those containing nitrogen substituents receiving particular attention [13,14,15]. Owing to indole alkaloids privileged scaffold, coupled with high bioactivity, including their ability as anticancer agents [16], our ongoing research has been focused on the generation of small libraries of indole alkaloids, from the African medicinal plant *Tabernaemontana elegans*, through isolation and further derivatization, to establish structure–activity relationships, concerning their ability for reversing MDR [13,14,15,17]. The monoterpene indole alkaloid epimers dregamine (**1**) and tabernaemontanine, isolated from *T. elegans* [13]*,* were previously functionalized at the indole nitrogen, giving rise to several *N*-alkylated derivatives with a significant enhancement in the P-gp inhibitory activity, when compared to the parental compounds [13]. More precisely, it was found that compounds sharing *N*-phenethyl motifs strongly inhibited the P-gp efflux activity [13]. Likewise, the indole alkaloid scaffold was functionalized in the carbonyl group. The highest and selective P-gp inhibitory activity was found for compounds with a *para*-methylbenzylidene moiety, whereas other compounds with different substituents were selective for MRP1 [14]. Recently, using a different anti-MDR approach, we have identified an indole alkaloid derivative as an inhibitor of homologous DNA repair by disrupting the breast cancer susceptibility protein BRCA1 interaction with its binding partner, BRCA1-associated ring domain protein (BARD1), in triple-negative breast and ovarian cancers [17].

Therefore, taking into account the previous encouraging results, the present study aimed at preparing novel derivatives for increasing our pool of analogs and thus establishing new structure–activity relationships for optimizing their structures. Thus, by manipulating the dregamine carbonyl at C-3, through the insertion of new substituents containing nitrogen atoms together with aromatic rings, 30 (**3**–**32**) new compounds were prepared. Their ability to inhibit P-gp drug efflux activity was assessed, by flow cytometry, in human *ABCB1*-gene transfected mouse T-lymphoma cells. Moreover, the antiproliferative activity and the in vitro interaction between the compounds and the antineoplastic drug doxorubicin were also evaluated. The type of interaction between selected derivatives and P-gp was also addressed through the ATPase activity assay. Molecular docking studies were performed to identify the preferred DBS of the derivatives within the P-gp poly-specific drug-binding pocket. A quantitative structure–activity relationship (QSAR) model was also generated for a better comprehension of which molecular descriptors may affect the biological activity.

## 2. Results and Discussion

### 2.1. Chemistry

The monoterpene indole alkaloid of the corynanthe-type [18], dregamine (**1**), was previously isolated, in a large amount, from the alkaloid fraction of the methanol extract of *Tabernaemontana elegans* roots [13]. As mentioned above, dregamine (**1**) derivatives were reported as promising MDR reversers [13,14,15], prompting us to carry out new chemical modifications to obtain 19 azines (**3**–**21**) and 11 semicarbazone (**22**–**32**) derivatives.

**Scheme 1:**
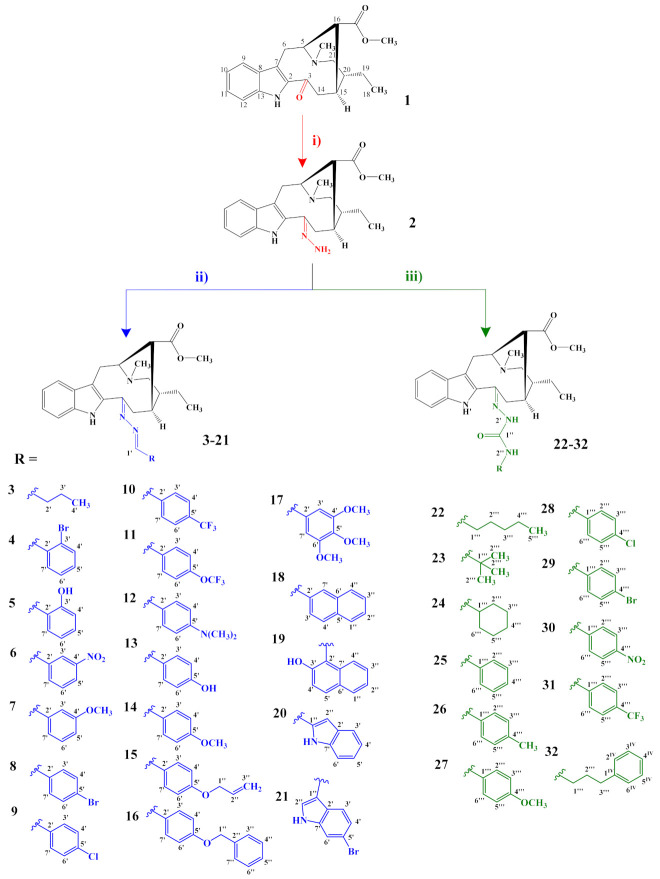
Preparation of azines **3**–**21** and semicarbazones **22**–**32**. Reagents and conditions: (i) hydrazine monohydrate solution 98% (5 equiv.) in MeOH, reflux, overnight. (ii) Aldehyde (5–6 equiv.), in EtOH, acetic acid (cat.), rt, 2 h. (iii) isocyanate (1.2–1.5 equiv.) in THF, 70 °C, 1 h.

Initially, a considerable amount of dregamine (**1**) was condensed with hydrazine monohydrate solution to give rise to dregamine hydrazone intermediate **2** (Scheme 1, **i**). Thereafter, compound **2** reacted with butyraldehyde or different benzaldehydes, naphthylaldehydes, and indolecarboxaldehydes to afford 19 new azines (**3**–**21)** (Scheme 1, **ii**). In turn, the reaction of compound **2** with aliphatic (pentyl, *tert*-butyl, cyclohexyl) or aromatic (phenyl, phenylpropyl) isocyanates yielded 11 new semicarbazone derivatives (**22**–**32**) (Scheme 1, **iii**).

The structures of the compounds were elucidated mainly by comparing their ^1^H and ^13^C NMR data with those of dregamine (**1**), coupled with two-dimensional NMR experiments (COSY, HMQC, and HMBC). When comparing the NMR data of compounds **3**–**21** with those of compound **1**, the main differences were additional carbon and proton signals owing to the new substituent, such as a downfield triplet observed for compound **3** (δ_H_ 7.86) and a singlet for compounds **4**–**21** (δ_H_ 8.45–9.55) in the ^1^H NMR spectra, which were assigned to the H-1′ (–C=N–N=CHR). Regarding the ^13^C NMR data, as expected, strong diamagnetic effects were observed at C-3 (δ_C_ 159.5–163.1), when compared with those of **1** (δ_C_ 191.6) as well as a carbon resonance (δ_C_ 150.7–162.9) assignable to a second imine function (–C=N–N=CHR).

Similarly, in compounds **22**–**32**, the semicarbazone structural feature (–C=N–NH–CO–NHR) was easily recognized in the ^1^H NMR spectra through a singlet, without correlation in the HMQC spectrum, at δ_H_ 7.58–8.82, assignable to the exchangeable NH-2′ protons (–C=N–NH–CO–NHR), whereas the NH-2″ (–C=N–NH–CO–NHR) proton signal was observed with different multiplicity and location, depending on each substituent, namely, as a singlet (δ_H_ 5.97, **23**; δ_H_ 8.11–8.59, **25**–**31**), doublet (δ_H_ 6.15, **24**), or triplet (δ_H_ 6.23–6.27, **22** and **32**). The assignment of the exchangeable NH protons signals, which were removed with the addition of deuterium oxide after heating (Supplementary Information), was substantiated by the cross-peaks observed in the ^1^H–^1^H COSY spectra (compounds **22**, **24,** and **32**) (Supplementary Information) and ^2^*J*_C__–H_ and ^3^*J*_C__–H_ heterocorrelations observed in the HMBC data between the NH-2′ proton and the carbonyl C-1″ and the imine carbon C-3. In turn, for the NH-2″ proton heterocorrelations with C-1‴, C-2‴, and C-6‴ were observed, depending on the substituent. Additionally, the semicarbazone moiety was corroborated in the ^13^C NMR spectra by the strong diamagnetic effect at C-3 corresponding to C=N (δ_C_ 143.1–149.1) and the presence of a shielded carbonyl group (δ_C_ 154.1–156.7) due to the monomeric effects of the adjacent nitrogen atoms (–C=N–NH–CO–NHR).

### 2.2. Biological Activity

#### 2.2.1. In Vitro Antiproliferative Assay and Collateral Sensitivity Effect

The antiproliferative activity of dregamine (**1**) and derivatives (**3**–**32**) was assessed through the thiazolyl blue tetrazolium bromide (MTT) assay on sensitive L5178Y mouse T-lymphoma cells (PAR) and corresponding resistant human *ABCB1*-gene transfected L5178Y subline (MDR). Non-cancer mouse embryonic fibroblasts (NIH/3T3) were also used. The results were obtained in terms of the concentration of the compound causing 50% inhibition (IC50), as shown in Table 1.

With the exception of compound **3**, all derivatives were found to have higher antiproliferative activity (IC_50_ values ranging between 5.43 ± 0.38 and 26.40 ± 0.53, PAR cells; 4.28 ± 0.25 and 12.91 ± 0.35, MDR cells) than the parental compound **1** (IC_50_ = 37.21 ± 4.99, PAR cells; 22.97 ± 0.48, MDR cells) on both sensitive and resistant cells. It is noteworthy that all compounds but **19**, **20**, and **29** were proved to have a stronger antiproliferative effect against the resistant cell line when compared to the parental one. Thus, in order to evaluate their potential collateral sensitivity effect, relative resistance (RR) values were determined as the ratio between the IC_50_ of a compound against a resistant subline and the IC_50_ against the corresponding parental line. Compounds having an RR < 1 show selectivity against the MDR cells, whereas RR ≤ 0.5 means that the CS effect occurs [20]. As can be observed in Table 1, compounds **5**, **7**, **9**, **11**, **13**, **14**, **23**, and **24** exhibited RR ≤ 0.5, pointing out their potential as CS agents.

This set of indole alkaloid derivatives possess metal-chelating properties that may explain, at least partially, their CS effect in P-gp-overexpressing cells. In fact, taking into account that the most promising MDR-selective compounds reported in the literature are metal chelators, it is believed that this metal-chelating ability is responsible for increased cytotoxicity against MDR cells [21]. This assumption has been substantiated by the MDR-selective toxicity against P-gp-overexpressing cells of several strong metal-chelating thiosemicarbazones such as triapine [22,23,24]. Moreover, it was demonstrated that they do not act only as simple chelators, removing cellular metals, such as iron, but also as metal-interacting agents [25,26].

In a previous study, we have found that some indole alkaloid derivatives, mostly sharing a new aliphatic azine moiety, showed CS activity in MRP1-overexpressing cancer cells. Furthermore, some of these compounds were able to induce MRP1-mediated glutathione efflux, thus increasing its intracellular depletion [14].

In addition, the antiproliferative activity of the compounds was also evaluated on non-cancerous mouse embryonic fibroblast cells (NIH/3T3), whose values were compared with those of PAR and MDR cells by evaluation of the selective index (SI) values. As it can be seen in Table 1, the SI values indicated that most of the compounds exerted a selective activity toward mouse T-lymphoma cells, mainly against the drug-resistant subline (SI_C/B_ > SI_C/A_). The highest selective index values (SI_C/A_ = 9.39; SI_C/B_ = 10.79) were found for the derivative bearing the trimethoxyphenyl substituent (**17**).

#### 2.2.2. Inhibition of P-Glycoprotein Efflux Activity

The evaluation of compounds’ ability for inhibiting P-gp efflux activity was assessed using the rhodamine-123 functional assay by flow cytometry on sensitive mouse T-lymphoma cell line (L5178Y-PAR) and the corresponding human *ABCB1*-transfected MDR subline (L5178Y-MDR). Fluorescence activity ratio (FAR) values were calculated, measuring the quotient between the intracellular accumulation of rhodamine-123 in resistant and sensitive cancer cells. All compounds were tested at 0.2 and 2 µM, and the corresponding results are summarized in Table 2 and Table 3. Verapamil (20 µM), a standard P-gp inhibitor, was used as a positive control. Inhibitory activity was assumed to take place when the FAR value was above 1, whereas the compounds were considered as strong inhibitors if the FAR ratio was higher than 10 [13].

The activity of the compounds was mostly observed in a concentration-dependent manner. When tested at the lowest concentration (0.2 µM), most of the azine derivatives were found to be active, with compounds **12**, **17**–**19** exhibiting strong (FAR > 10) P-gp inhibitory activities (FAR values ranging between 11.57 and 30.74) (Table 2). At 2 µM, it was found that, with the exception of compound **13**, the azines bearing aromatic substituents (**4**–**12**, **14**–**21**) strongly inhibited the P-gp efflux activity (FAR values ranging from 15.69 to 128.48), having FAR values significantly higher than verapamil (up to 20-fold at a 10-fold lower concentration), those containing benzyloxybenzene, trimethoxyphenyl, naphthyl, or indolyl moieties (**16**–**21**) being the most active (FAR > 95.5). Conversely, no significant activity was found for the azine derivative with an aliphatic substituent (**3**).

As can be observed in Table 3, the semicarbazone derivatives (**22**–**32**) were much less active, having significant FAR values only at 2 µM. As with azine derivatives, compounds sharing aromatic substituents (**25**–**32**) showed higher activity (FAR values = 3.20–8.77) than those with aliphatic moieties (**22**–**24**).

These results clearly emphasized the relevance of extra aromatic motifs attached to the monoterpene indole alkaloid scaffold, whose contribution to the modulatory activity enhancement may be explained owing to additional electrostatic and π–π interactions between aromatic substituents and amino acid residues in the P-gp drug binding site. Interestingly, the effect of extra aromatic moieties was still more evident for derivatives **16**, **18**–**21**, bearing substituents with more than one aromatic ring, exhibiting remarkable inhibition (FAR > 95.5, at 2 µM).

Furthermore, another substantial increase of activity was observed when the number of methoxy groups augmented (**14**, FAR = 2.37 and 16.63 at 0.2 and 2 µM, respectively, vs. **17**, FAR = 11.99 and 100.07 at 0.2 and 2 µM, respectively), highlighting the relevance of H-bond acceptor groups for P-gp inhibition. The importance of the H-bonding potential was also illustrated by FAR values obtained for azine derivatives sharing hydroxy or methoxy groups at *ortho*- or *meta*-positions (**5**, FAR = 3.16 and 22.72 at 0.2 and 2 µM, respectively; **7**, FAR = 7.54 and 25.06 at 0.2 and 2 µM, respectively) that revealed higher activity than those located at the *para*-position (**13**, FAR = 1.31 and 5.25 at 0.2 and 2 µM, respectively; **14**, FAR = 2.37 and 16.63 at 0.2 and 2 µM, respectively).

When comparing FAR values obtained at the lowest concentration (0.2 µM) for the azine derivatives bearing mono-substituted phenyl groups (**4**–**16**), compound **12** was the most active, corroborating that the presence of a tertiary nitrogen atom is also an important feature for P-gp modulation [27].

It is well known that several physico-chemical properties can be related to P-gp efflux activity including, lipophilicity, topological surface area (TPSA), and H-bond acceptors (HBA) and donors (HBD) [27]. However, P-gp inhibitors are structurally diverse, and therefore, it is not clear which common features directly contribute to the protein inhibition. In this work, it was noticed that the semicarbazones (**22**–**32**), which showed lower FAR values, have higher TPSA and numbers of hydrogen bond donors (TPSA ranging from 98.8 Å^2^ to 144.6 Å^2^; HBD = 3; Supplementary Information) in comparison to the azines set (TPSA ranging from 70.1 Å^2^ to 115.9 Å^2^; HBD between 1 and 2; Supplementary Information), which may contribute to the differences obtained in the biological activity.

Interestingly, several compounds with strong activity in the rhodamine accumulation (FAR > 10) also exhibited significant (IC_50_ < 10 µM) and selective (RR < 1) antiproliferative activity in MDR cells. Among them, compounds **5**, **7**, **9**, **11**, and **14** showed a collateral sensitivity effect (RR < 0.5).

#### 2.2.3. Checkerboard Combination Assay

**Figure 1:**
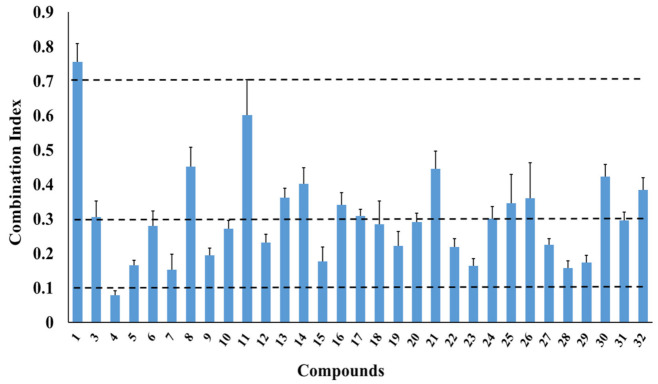
Effect of compounds (**1**, **3**–**32**) in the antiproliferative activity of doxorubicin, in drug combination assays, on human *ABCB1*-transfected L5178Y mouse lymphoma cells (L5178Y. Combination index (CI) parameter is the mean of three CI values determined based on different drug ratios ± standard deviation (SD), for an inhibitory concentration of 50% (IC_50_). CI < 0.1: very strong synergism; 0.1 < CI < 0.3: strong synergism; 0.3 < CI < 0.7: synergism; 0.7 < CI < 0.9: moderate to slight synergism [29].

The in vitro interactions between the compounds and the well-known antitumor drug and P-gp substrate, doxorubicin, were evaluated in a combination chemotherapy model on human *ABCB1*-transfected mouse T-lymphoma cells. The nature of drug–drug interactions was assessed by determination of the combination index (CI) using the Chou and Talalay method (Figure 1), and therefore evaluated as synergistic (CI < 1), additive (CI = 1), or antagonistic (CI > 1) [28].

As it can be seen in Figure 1, all compounds exhibited a synergistic behavior (CI < 1) when co-administered with doxorubicin, thus substantiating the results obtained in the transport assay. Among them, compound **4** showed very strong synergism (CI < 0.1), whereas other derivatives (**5**–**7**, **9**, **10**, **12**, **15**, **18**–**20**, **22**, **23**, **27**–**29**, and **31**) exhibited strong synergism with the anticancer drug. On the other hand, compounds inactive or with weak activity in the transport assay, such as the derivatives with aliphatic substituents (**3**, **22**–**24**), also enhanced the cytotoxicity of doxorubicin in a synergistic mode, thus suggesting that a different type of mechanism for re-sensitizing the MDR phenotype may occur.

#### 2.2.4. P-gp ATPase Activity Assay

Once it was established that ATP hydrolysis is directly associated with the P-gp efflux activity [8], the interaction mode between selected compounds (**12**, **17**, **18**, **20**, and **29**) and this transporter protein was further investigated using human P-gp membranes in the ATPase activity assay (P-gp-Glo^TM^) [30] to gather insights about their interaction with P-gp activity, namely, as stimulators or inhibitors. This ATPase activity assay is based on the ATP dependence of the light-generating reaction of firefly luciferase. After incubating P-gp with ATP, the reaction is stopped, and the ATP consumption by P-gp is given by a luciferase-generated luminescent signal due to the remaining unmetabolized ATP. Thus, a greater decrease in the signal means a higher P-gp activity [30].

**Figure 2:**
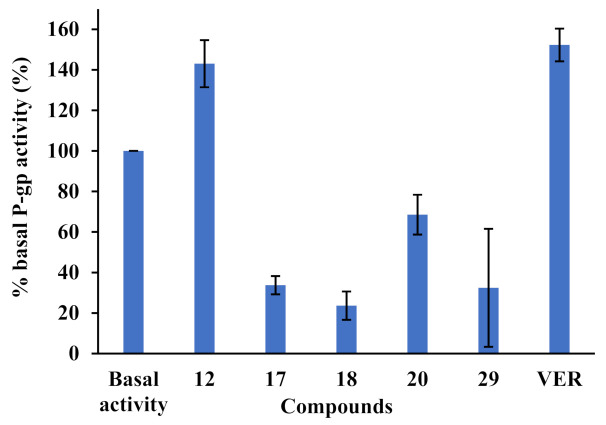
Effect of compounds **12**, **17**, **18**, **20**, **29**, and verapamil (VER) on P-gp ATPase activity. The compounds are identified as P-gp substrates if they stimulate its basal activity (>100%) or as modulators when inhibiting basal activity (<100%). Compounds were tested at 25 µM and verapamil at 0.5 mM. Results were calculated as the means ± SD from experiments performed in triplicates.

Sodium orthovanadate (Vanadate, Na_3_VO_4_) is an inhibitor of P-gp ATPase activity; thus, after treating samples with vanadate, no P-gp-dependent ATP consumption is observed [30]. Therefore, the P-gp basal ATP consumption (basal activity) is defined as the difference between the luminescent signal of samples treated with vanadate and untreated. Consequently, the tested compounds were ranked as stimulators or inhibitors by comparing their P-gp ATPase activity with the basal activity. Verapamil (0.5 mM), a known P-gp substrate and activator of the ATPase activity of this transporter, thus causing a decrease in the percentage of luminescence of luciferase when compared with untreated samples (Figure 2), was used as control. The results are represented in Figure 2 as the difference between the luminescence of luciferase observed when treated with the compounds and with vanadate in comparison with the basal activity (100%).

As it could be observed in Figure 2, at the concentration used, an increase in the luminescent signal in relation to the untreated sample (% basal P-gp activity < 100%) was observed for compounds **17**, **18**, **20**, and **29**, thus behaving as inhibitors of P-gp ATPase activity. In this way, they may possibly act by binding to a P-gp allosteric residue, decreasing its efflux activity and consequently inhibiting the ATPase activity, or by directly blocking ATP hydrolysis by binding to the P-gp ATP binding site (non-competitive P-gp inhibitors). Conversely, compound **12** showed a similar behavior of the P-gp substrate verapamil, increasing the ATP consumption by stimulating the P-gp activity, thus suggesting that, at the concentration tested, it acts probably as a P-gp substrate that may, competitively, inhibit the efflux of other P-gp substrates.

### 2.3. In Silico Studies

#### 2.3.1. Molecular Docking

**Figure 3:**
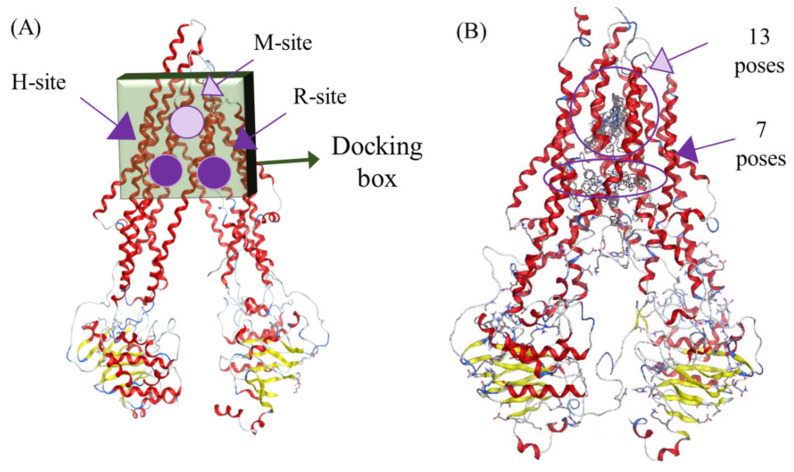
(**A**) Crystallographic structure of the P-gp transporter obtained from Protein Data Bank (PDB: 3G5U). (**B**) Twenty best docking poses of compound **16** in P-gp.

Compounds **1**, **3**–**32** were docked inside the P-gp internal drug-binding pocket [7], and the relative location of top-ranked binding poses was assessed to classify the molecules as modulators (M-site) or substrates (R- and H-sites), as previously reported [10] (Figure 3A). The 20 best poses were visualized, and the main results are displayed in Table 4, coupled with the experimental FAR values at 2 µM, as performed in our previous study. It should be noticed that most derivatives preferred to bind at M-site, and even those that had their best poses at substrate-binding sites (excluding compounds **10** and **11**) either had similar modulator-binding energy or a fewer number of poses compared to those found in the M-site.

According to our previous studies, it was proposed that the type, number, and distribution of interactions between a molecule bridging *N*-terminal and *C*-terminal P-gp halves (cross-interactions) could impact the P-gp ability for conformational changes and consequently have an influence on the efflux phenomenon [10,31]. Therefore, analysis of the interactions between P-gp and the best pose at the M-site was accomplished to determine the cross-interaction capability (CIc) for each molecule (Supplementary Information of [10]). Then, for a better understanding of the MDR-reversal capability of the derivatives, an overall view including binding energies, total number of interactions, and cross-interaction capabilities was performed, in which the main results are summarized in Table 4. These results showed that compounds **4**, **6**, **16**, and **19** had their best pose at the M-site and exhibited a strong binding affinity with the target coupled with strong cross-interactions with both P-gp halves, and therefore were considered as non-competitive inhibitors.

Additionally, by analyzing Table 4, it is clear that among the azine derivatives (**3**–**21**), the lowest binding energy was obtained for compound **16** (ΔG = −11.5 kcal.mol^−1^), with most poses docked at M-site (13 poses, Figure 3B). Despite compounds **10** and **11** also showing strong binding affinity with P-gp, the discrepancy in FAR values between compounds **10**, **11** (FAR ≈ 16), and **16** (FAR ≈ 105) cannot only be explained by the lowest binding energies obtained in the modulator binding site for compound **16** and substrate binding sites for compounds **10** and **11**. In effect, compound **16** showed more poses docked in the M-site together with stronger cross-interaction capability when compared to **10** and **11**. Therefore, compounds **10** and **11** are suggested as prototypes of competitive inhibitors.

Another observation showed that among the compounds bearing phenyl substituents displaying moderate affinity with P-gp (**5**, **7**, **12**–**15**), those having a weaker CIc match with lower experimental FAR values (compound **13**, FAR = 5.25; compound **14**, FAR = 16.63). An exception to this tendency was observed for compound **5**, which can possibly be elucidated by the much high number of poses docked in the M-site (16 poses) in comparison to the substrate-binding sites (R- and H-site).

The compounds with the highest FAR values (**16**–**21**) have shown both moderate to strong affinity for the M-site and CIc capability. In this classification, the compound bearing the trimethoxyphenyl substituent (**17**) is an outlier owing to its moderate affinity, in which the best M-pose obtained was the fifth, coupled with weak cross-interactions with both P-gp halves. However, although compound **17** is among the compounds having the lowest binding affinity to the M-site, it also had a moderate affinity to the substrate-binding sites, making this compound a prototype for competitive modulation through both sites. Unfortunately, the semicarbazones set (**22**–**32**) did not show any direct match between the FAR values and the virtual screening results presented herein and used previously to rationalize the activity in other sets of compounds [31]. In this regard, it should be highlighted that several physicochemical properties are not considered in this model, and therefore the virtual screening results were coupled with QSAR analysis in the next section for a better elucidation between molecular features and the biological activity.

#### 2.3.2. QSAR Modeling

Firstly, an extensive database of molecular descriptors (topological, geometrical, and constitutional) was generated, obtaining hundreds of physicochemical properties for each molecule using E-DRAGON, MOE, and PaDEL programs to isolate each descriptor that individually contributed the most to the molecule’s potency. Afterward, FAR values were added to the dataset, and a search for the most significant combination of molecular descriptors in each database was performed using WEKA software. The regression results were split between the two sets of compounds (azines **3**–**21** and semicarbazones **22**–**32**), and the coefficient of determination values for representative descriptors are shown in Table 5.

As it can be observed in Table 5, some physicochemical descriptors related to molecular shape and branching (EEig11x, X5, WTPT-2) and atomic contributions to log*P* (GCUT_SLOGP_3) or molar refractivity (GCUT_SMR_3) were found to have an influence on P-gp inhibitory activity for the compounds with azine substituents attached to the main scaffold. In contrast, the experimental FAR values for the semicarbazones did not show the same tendency, clearly demonstrating that they behave as a class with completely different outcomes regarding activity and are in agreement with the molecular docking analysis.

In order to allow a better understanding of which structural features in dregamine derivatives (**3**–**32**) contribute the most to P-gp modulatory activity, a QSAR model was built using the most suitable descriptors from E-DRAGON, MOE, and PaDEL software programs and assessed in the WEKA program through the select attributor tool (see the experimental section). After the reduction of the molecular descriptors, a QSAR model was generated in WEKA using the following multivariate linear regression (LR):

FAR_pred_ = 17.089 ∗ h_logD − 345.73 ∗ vsurf_HL2 +1418.98 ∗ PW4 − 849.21 ∗ BIC3 + 80.30 ∗ BEHv2 − 623.71 (1)

**Figure 4:**
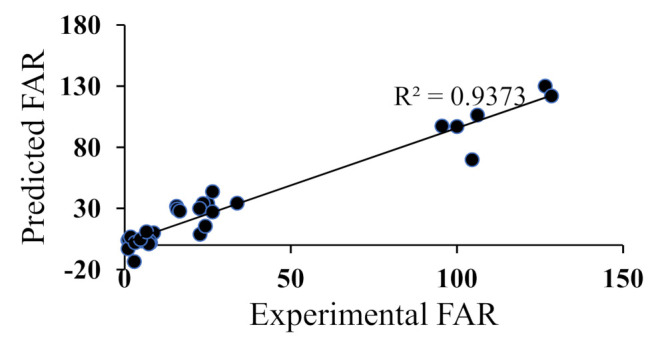
Plot of experimental vs. predicted activity at 2 µM, using the linear regression (LR) model.

The statistical data for the LR model (Figure 4) can be seen in Table 6, in which an R^2^ of 0.937 was obtained, with a mean absolute error (MAE) and root mean squared error (RMSE) of 6.941 and 9.998, respectively. The internal and external validations of the model were performed through the 10-fold cross-validation test and test set methods. The corresponding cross-validation parameter, q^2^, and squared correlation for the test set, R^2^_pred_, showed values of 0.887 and 0.763, respectively, confirming the reliability of the model.

Regarding the regression coefficient values observed in Equation (1), it is possible to verify that descriptor path/walk 4—Randic shape index (PW4) is the most relevant. This topological descriptor introduced by Randic [32] increases with increased branching in the vertices, showing a positive influence of branching on biological activity. The second most significant descriptor verified in this regression was the bond information content (BIC_3_) index (neighborhood symmetry of three-order) [33], indicating that the higher the edge’s number, the lower the BIC value. Consequently, the negative coefficient of BIC_3_ shows that the biological activity is estimated to increase for compounds with more edges. The highest eigenvalue No. 2 of the burden matrix/weighted by atomic van der Waals volumes (BEHv2) [34] also influences the biological activity in a positive manner. Thus, the P-gp inhibition tends to increase with the size of the molecule. Finally, other descriptors that proved to correlate with activity were the second hydrophilic-lipophilic balance (vsurf_HL2) and the octanol/water distribution coefficient at pH 7, calculated as a state average (h_logD). As expected, for more lipophilic compounds, the FAR parameter is estimated to increase, which is in agreement with our previous study [13].

In order to increase the robustness and predictability of the study, different QSAR models using machine-learning methods were applied, maintaining the set of descriptors selected above: artificial neural network (MLPRegressor) and support vector machine (SMOReg). As can be seen in Table 6, both models showed R^2^ and q^2^ values above 0.7, proving the reliability of the models.

## 3. Materials and Methods

### 3.1. General Experimental Procedures

All solvents were dried according to publish methods and distilled prior to use. Other reagents obtained from commercial suppliers were used without further purification. Low-resolution mass spectrometry was performed in a Triple Quadrupole mass spectrometer (Micromass Quattro Micro API, Waters). NMR spectra were recorded on a Bruker 300 Ultra-Shield instrument (^1^H 300 MHz, ^13^C 75 MHz). ^1^H and ^13^C NMR chemical shifts are expressed in δ (ppm), referenced to CDCl_3_ solvent, with the corresponding proton coupling constants (*J*) in Hertz (Hz). NMR spectra were assigned using appropriate DEPT, COSY, HSQC, and HMBC sequences. Column chromatography was performed on silica gel (Merck 9385). TLC was performed on precoated Merck silica gel 60 F_254_ plates, with visualization under UV light (λ 254 and 366 nm) and by spraying either with Dragendorff’s reagent or a solution of H_2_SO_4_-MeOH (1:1), followed by heating.

### 3.2. Test Compounds

Dregamine (**1**) was isolated from the MeOH extract of *Tabernaemontana elegans* roots, as previously reported [13].

The hydrazone derivative (**2**) was obtained from reaction of dregamine (**1)** (1 equiv.) with hydrazine monohydrate solution 98% (5 equiv.) dissolved in MeOH. The mixture was stirred under reflux overnight. Afterward, the reaction mixture was extracted with EtOAc (3 × 50 mL). After drying the organic layers with Na_2_SO_4_, the solvent was evaporated under vacuum at 40 °C, and the resulting residue was submitted to flash chromatography (silica gel, CH_2_Cl_2_/MeOH, 1:0 to 49:1). The preparation of the new derivatives **3**–**32** is described below.

#### 3.2.1. General Preparation of Dregamine Azines **3–21**

Compound (**2**) (15–32 mg, 1 equiv.) was dissolved in ethanol (EtOH, 2 mL) with suitable aldehyde (5–6 equiv.), and a catalytic amount of acetic acid was added. The mixture was stirred, at room temperature and under nitrogen atmosphere, for 2 h. After evaporating the solvent, under vacuum at 40 °C, the residue was submitted to flash chromatography and further purified by preparative TLC.

*3-[(butylidene)hydrazineylidene]dregamine* (**3**). Obtained from reaction of compound **2** (20 mg, 0.054 mmol, 1 equiv.) with butyraldehyde (29 µL, 0.33 mmol, 6 equiv.). After stirring the mixture for 2 h at room temperature, the reaction product was sequentially purified by column chromatography (silica gel, CH_2_Cl_2_/MeOH, 1:0 to 97:3) and preparative TLC (CH_2_Cl_2_/MeOH, 97:3) to afford 6 mg (0.014 mmol, yield 26%) of a yellow oil. ^1^H NMR (300 MHz, MeOD) δ 7.86 (1H, t, *J* = 5.4 Hz, H-1′), 7.60 (1H, bd, *J* = 8.0 Hz, H-9), 7.33 (1H, bd, *J* = 8.1 Hz, H-12), 7.20 (1H, m, H-11), 7.05 (1H, m, H-10), 3.94 (1H, m, H-5), 3.63 (1H, dd, *J* = 13.1, 6.1 Hz, H-14a), 3.15 (1H, dd, *J* = 14.7, 10.3 Hz, H-6a), 2.72–2.64* (5H, H-6b, H-21b, –COOMe), 2.62 (1H, m, H-15), 2.60–2.49* (5H, H-16, H-21a, *N*-Me), 2.47–2.31* (3H, H-14b, H-2′), 1.81 (1H, m, H-20), 1.68 (2H, m, H-3′), 1.43–1.34* (2H, m, H-19), 1.09–1.01* (6H, H-18, H-4′) ppm; ^13^C NMR (75 MHz, MeOD) δ 172.0 (–COOMe), 162.8 (C-1′), 160.0 (C-3), 138.0 (C-13), 133.6 (C-2), 130.5 (C-8), 125.4 (C-11), 120.2 (C-10), 120.0 (C-9), 117.5 (C-7), 112.2 (C-12), 58.9 (C-5), 50.9 (–COOMe), 50.0–48.0* (C-16, C-21), 44.1 (C-20), 42.2 (*N*-Me), 36.0 (C-2′), 31.8 (C-15), 25.4 (C-14), 24.3 (C-19), 21.2 (C-3′), 20.7 (C-6), 14.2 (C-4′), 11.5 (C-18) ppm. * signals overlapped. ESIMS *m*/*z* 423 (M + H)^+^.

*3-[(2″-bromobenzylidene)hydrazineylidene]dregamine* (**4**). Obtained from reaction of compound **2** (32 mg, 0.087 mmol, 1 equiv.) with 2-bromobenzaldehyde (60 µL, 0.52 mmol, 6 equiv.). After stirring the mixture for 2 h at room temperature, the reaction product was sequentially purified by column chromatography (silica gel, CH_2_Cl_2_/MeOH, 1:0 to 98.5:1.5) and preparative TLC (CH_2_Cl_2_/MeOH, 49:1) to afford 13 mg (0.023 mmol, yield 28%) of an amorphous yellow powder. ^1^H NMR (300 MHz, MeOD) δ 8.91 (1H, s, H-1′), 8.12 (1H, dd, *J* = 7.6, 1.8 Hz, H-4′), 7.63–7.55* (2H, m, H-9, H-7′), 7.39 (1H, bd, *J* = 7.9 Hz, H-12), 7.36–7.28* (2H, H-5′, H-6′), 7.23 (1H, m, H-11), 7.07 (1H, dd, *J* = 7.9, 7.1 Hz, H-10), 3.90 (1H, m, H-5), 3.79 (1H, dd, *J* = 13.2, 6.5 Hz, H-14a), 3.15 (1H, dd, *J* = 14.9, 8.2 Hz, H-6a), 2.85 (1H, dd, *J* = 15.1, 10.0 Hz, H-6b), 2.73–2.63* (5H, H-15, H-21b, –COOMe), 2.51–2.42* (5H, H-16, H-21a, *N*-Me), 2.19 (1H, t, *J* = 13.1, H-14b), 1.78 (1H, m, H-20), 1.38 (2H, m, H-19), 1.04 (3H, t, *J* = 7.3 Hz, H-18) ppm; ^13^C NMR (75 MHz, MeOD) δ 171.9 (–COOMe), 162.9 (C-3), 158.3 (C-1′), 138.3 (C-13), 134.9 (C-2′), 134.4 (C-7′), 133.7 (C-2), 133.1 (C-6′), 130.4 (C-8), 129.4 (C-4′), 128.7 (C-5′), 126.2 (C-3′), 125.9 (C-11), 120.4 (C-10), 120.2 (C-9), 118.4 (C-7), 112.5 (C-12), 58.7 (C-5), 51.0 (–COOMe), 50.0–48.0* (C-16, C-21), 43.9 (C-20), 42.0 (*N*-Me), 31.6 (C-15), 25.5 (C-14), 24.2 (C-19), 21.3 (C-6), 11.4 (C-18) ppm. * signals overlapped. ESIMS *m*/*z* 537 (M + H)^+^.

*3-[(2″-hydroxybenzylidene)hydrazineylidene]dregamine* (**5**). Obtained from reaction of compound **2** (27 mg, 0.073 mmol, 1 equiv.) with 2-hydroxybenzaldehyde (46 µL, 0.44 mmol, 6 equiv.). After stirring the mixture for 2 h at room temperature, the reaction product was sequentially purified by column chromatography (silica gel, CH_2_Cl_2_/MeOH, 1:0 to 97:3) and preparative TLC (CH_2_Cl_2_/MeOH, 97:3) to afford 25 mg (0.053 mmol, yield 72%) of an amorphous yellow powder. ^1^H NMR (300 MHz, MeOD/CDCl_3_) δ 8.63 (1H, s, H-1′), 7.62 (1H, bd, *J* = 8.0 Hz, H-9), 7.38 (1H, bd, *J* = 8.1 Hz, H-12), 7.35–7.25* (2H, H-5′, H-11), 7.21 (1H, dd, *J* = 7.4, 1.4 Hz, H-7′), 7.12 (1H, t, *J* = 7.2 Hz, H-10), 6.99 (1H, d, *J* = 8.2 Hz, H-4′), 6.87 (1H, t, *J* = 7.4 Hz, H-6′), 3.88 (1H, m, H-5), 3.40 (1H, m, H-14a), 3.17 (1H, dd, *J* = 14.8, 8.2 Hz, H-6a), 2.80 (1H, dd, *J* = 14.6, 10.2 Hz, H-6b), 2.72–2.61* (5H, H-15, H-21b, –COOMe), 2.48–2.37* (4H, H-16, *N*-Me), 2.29–2.10* (2H, H-14b, H-21a), 1.76 (1H, m, H-20), 1.46 (1H, m, H-19a), 1.31 (1H, m, H-19b), 1.04 (3H, t, *J* = 7.3 Hz, H-18) ppm; ^13^C NMR (75 MHz, MeOD/CDCl_3_) δ 171.4 (–COOMe), 163.4 (C-3′), 161.1 (C-3), 159.7 (C-1′), 137.0 (C-13), 132.9 (C-5′), 132.6 (C-7′), 132.5 (C-2), 129.5 (C-8), 125.7 (C-11), 120.1 (C-10), 120.0 (C-9), 119.6 (C-6′), 118.7 (C-7), 118.3 (C-2′), 116.8 (C-4′), 111.7 (C-12), 57.6 (C-5), 50.7 (–COOMe), 50.0–48.0* (C-16, C-21), 43.6 (C-20), 42.0 (*N*-Me), 31.5 (C-15), 25.2 (C-14), 23.9 (C-19), 20.6 (C-6), 11.8 (C-18) ppm. * signals overlapped. ESIMS *m*/*z* 473 (M + H)^+^.

*3-[(3″-nitrobenzylidene)hydrazineylidene]dregamine* (**6**). Obtained from reaction of compound **2** (28 mg, 0.076 mmol, 1 equiv.) with 3-nitrobenzaldehyde (69 mg, 0.46 mmol, 6 equiv.). After stirring the mixture for 2 h at room temperature, the reaction product was sequentially purified by column chromatography (silica gel, CH_2_Cl_2_/MeOH, 1:0 to 97:3) and preparative TLC (CH_2_Cl_2_/MeOH, 97:3) to afford 10 mg (0.019 mmol, yield 26%) of an amorphous yellow powder. ^1^H NMR (300 MHz, MeOD/CDCl_3_) δ 8.73 (1H, t, *J* = 1.6 Hz, H-3′), 8.68 (1H, s, H-1′), 8.28 (1H, dd, *J* = 8.2, 1.4 Hz, H-5′), 8.11 (1H, bd, *J* = 7.7 Hz, H-9), 7.70–7.64* (2H, H-6′, H-7′), 7.40 (1H, bd, *J* = 8.2 Hz, H-12), 7.28 (1H, t, *J* = 7.4 Hz, H-11), 7.12 (1H, t, *J* = 7.4 Hz, H-10), 4.08 (1H, m, H-5), 3.99 (1H, dd, *J* = 13.1, 6.3 Hz, H-14a), 3.38 (1H, m, H-6a), 3.15 (1H, dd, *J* = 14.5, 10.3 Hz, H-6b), 2.75–2.87* (3H, H-15, H-21a, H-21b), 2.57–2.70* (7H, *N*-Me, –COOMe, H-16), 2.40 (1H, t, *J* = 13.0 Hz, H-14b), 1.92 (1H, m, H-20), 1.52 (2H, m, H-19), 1.16 (3H, t, *J* = 7.3 Hz, H-18) ppm; ^13^C NMR (75 MHz, MeOD/CDCl_3_) δ 171.0 (–COOMe), 162.9 (C-3), 157.2 (C-1′), 149.2 (C-4′), 137.3 (C-13), 137.3 (C-2′), 134.8 (C-7′), 132.9 (C-2), 130.5 (C-6′), 129.5 (C-8), 125.8 (C-11), 125.4 (C-3′), 122.6 (C-5′), 120.2 (C-10), 119.7 (C-9), 117.6 (C-7), 111.8 (C-12), 58.2 (C-5), 50.9 (–COOMe), 50.0–48.0* (C-16, C-21), 42.9 (C-20), 41.8 (*N*-Me), 30.5 (C-15), 24.9 (C-14), 23.6 (C-19), 21.0 (C-6), 11.1 (C-18) ppm. * signals overlapped. ESIMS *m*/*z* 502 (M + H)^+^.

*3-[(3″-methoxybenzylidene)hydrazineylidene]dregamine* (**7**). Obtained from reaction of compound **2** (23 mg, 0.062 mmol, 1 equiv.) with 3-methoxybenzaldehyde (51 mg, 0.37 mmol, 6 equiv.). After stirring the mixture for 2 h at room temperature, the reaction product was sequentially purified by column chromatography (silica gel, CH_2_Cl_2_/MeOH, 1:0 to 97:3) and preparative TLC (CH_2_Cl_2_/MeOH, 97:3) to afford 9 mg (0.019 mmol, yield 30%) of an amorphous yellow powder. ^1^H NMR (300 MHz, MeOD) δ 8.50 (1H, s, H-1′), 7.56 (1H, bd, *J* = 8.0 Hz, H-9), 7.42 (1H, m, H-6′), 7.37 (1H, bd, *J* = 8.2 Hz, H-12), 7.34–7.28* (2H, H-3′, H-5′), 7.21 (1H, ddd, *J* = 8.1, 7.0, 1.0 Hz, H-11), 7.05 (1H, ddd, *J* = 8.0, 7.1, 1.0 Hz, H-10), 7.00 (1H, m, H-7′), 3.87–3.76* (5H, H-5, H-14a, 4′-OCH_3_), 3.13 (1H, dd, *J* = 14.9, 8.2 Hz, H-6a), 2.90 (1H, dd, *J* = 14.7, 10.1 Hz, H-6b), 2.72–2.63* (4H, H-21b, –COOMe), 2.61 (1H, m, H-15), 2.47–2.29* (5H, H-16, H-21a, *N*-Me), 2.22 (1H, t, *J* = 12.8 Hz, H-14b), 1.75 (1H, m, H-20), 1.37 (2H, m, H-19), 1.04 (3H, t, *J* = 7.3 Hz, H-18) ppm; ^13^C NMR (75 MHz, MeOD) δ 172.5 (–COOMe), 162.5 (C-4′), 161.4 (C-3), 159.7 (C-1′), 138.2 (C-13), 137.8 (C-2′), 133.8 (C-2), 130.7 (C-6′), 130.5 (C-8), 125.6 (C-11), 122.7 (C-7′), 120.3 (C-10), 120.2 (C-9), 118.8 (C-5′), 118.3 (C-7), 112.7 (C-3′), 112.3 (C-12), 58.6 (C-5), 55.7 (O–CH_3_), 50.8 (–COOMe), 50.0–48.0* (C-16, C-21), 44.2 (C-20), 42.3 (*N*-Me), 31.7 (C-15), 25.5 (C-14), 24.3 (C-19), 21.3 (C-6), 11.5 (C-18) ppm. * signals overlapped. ESIMS *m*/*z* 487 (M + H)^+^.

*3-[(4″-bromobenzylidene)hydrazineylidene]dregamine* (**8**). Obtained from reaction of compound **2** (23 mg, 0.062 mmol, 1 equiv.) with 4-bromobenzaldehyde (58 mg, 0.31 mmol, 5 equiv.). After stirring the mixture for 2 h at room temperature, the reaction product was sequentially purified by column chromatography (silica gel, CH_2_Cl_2_/MeOH, 1:0 to 97:3) and preparative TLC (CH_2_Cl_2_/MeOH, 97:3) to afford 8 mg (0.015 mmol, yield 24%) of an amorphous yellow powder. ^1^H NMR (300 MHz, MeOD/CDCl_3_) δ 8.50 (1H, s, H-1′), 7.70 (2H, d, *J* = 8.6 Hz, H-3′, H-7′), 7.61–7.53* (3H, H-9, H-4′, H-6′), 7.37 (1H, bd, *J* = 8.2 Hz, H-12), 7.23 (1H, ddd, *J* = 8.1, 6.9, 1.0 Hz, H-11), 7.07 (1H, ddd, *J* = 8.0, 6.9, 0.9 Hz, H-10), 3.93–3.80* (2H, H-5, H-14a), 3.20 (1H, dd, *J* = 14.8, 8.2 Hz, H-6a), 3.01 (1H, dd, *J* = 14.6, 10.0 Hz, H-6b), 2.75–2.61* (5H, H-15, H-21b, –COOMe), 2.53 (1H, m, H-16), 2.48–2.37* (5H, H-14b, H-21a, *N*-Me), 1.80 (1H, m, H-20), 1.39 (2H, m, H-19), 1.05 (3H, t, *J* = 7.3 Hz, H-18) ppm; ^13^C NMR (75 MHz, MeOD/CDCl_3_) δ 172.3 (–COOMe), 162.5 (C-3), 158.2 (C-1′), 138.0 (C-13), 135.3 (C-2′), 133.5 (C-2), 132.9 (C-4′, C-6′), 130.5 (C-3′, C-7′), 130.3 (C-8), 125.7 (C-11), 125.7 (C-5′), 120.2 (C-10), 120.0 (C-9), 118.7 (C-7), 112.1 (C-12), 58.4 (C-5), 50.8 (–COOMe), 50.0–48.0* (C-16, C-21), 44.1 (C-20), 42.3 (*N*-Me), 31.6 (C-15), 25.4 (C-14), 24.2 (C-19), 21.2 (C-6), 11.4 (C-18) ppm. * Signals overlapped. ESIMS *m*/*z* 537 (M + H)^+^.

*3-[(4″-chlorobenzylidene)hydrazineylidene]dregamine* (**9**). Obtained from reaction of compound **2** (23 mg, 0.062 mmol, 1 equiv.) with 4-chlorobenzaldehyde (53 mg, 0.37 mmol, 6 equiv.). After stirring the mixture for 2 h at room temperature, the reaction product was sequentially purified by column chromatography (silica gel, CH_2_Cl_2_/MeOH, 1:0 to 49:1) and preparative TLC (CH_2_Cl_2_/MeOH, 49:1) to afford 11 mg (0.022 mmol, yield 36%) of an amorphous yellow powder. ^1^H NMR (300 MHz, MeOD) δ 8.50 (1H, s, H-1′), 7.75 (2H, d, *J* = 8.4 Hz, H-3′, H-7′), 7.59 (1H, bd, *J* = 7.9 Hz, H-9), 7.44–7.38* (3H, H-12, H-4′, H-6′), 7.25 (1H, t, *J* = 7.4 Hz, H-11), 7.09 (1H, t, *J* = 7.5 Hz, H-10), 3.89–3.73* (2H, H-5, H-14a), 3.10 (1H, m, H-6a), 2.83 (1H, m, H-6b), 2.71–2.58* (5H, H-15, H-21b, –COOMe), 2.44–2.32* (5H, H-16, H-21a, *N*-Me), 2.17 (1H, m, H-14b), 1.75 (1H, m, H-20), 1.36 (2H, m, H-19), 1.04 (3H, t, *J* = 7.3 Hz, H-18) ppm; ^13^C NMR (75 MHz, MeOD) δ 172.3 (–COOMe), 162.8 (C-3), 158.3 (C-1′), 138.2 (C-13), 137.5 (C-5′), 135.2 (C-2′), 133.8 (C-2), 130.5 (C-4′, C-6′), 130.5 (C-8), 130.0 (C-3′, C-7′), 125.7 (C-11), 120.3 (C-10), 120.2 (C-9), 118.7 (C-7), 112.3 (C-12), 58.5 (C-5), 50.9 (–COOMe), 50.0–48.0* (C-16, C-21), 44.1 (C-20), 42.1 (*N*-Me), 31.7 (C-15), 25.4 (C-14), 24.3 (C-19), 21.2 (C-6), 11.4 (C-18) ppm. * Signals overlapped. ESIMS *m*/*z* 491 (M + H)^+^.

*3-[(4″-(trifluoromethyl)benzylidene)hydrazineylidene]dregamine* (**10**). Obtained from reaction of compound **2** (15 mg, 0.041 mmol, 1 equiv.) with 4-trifluoromethylbenzaldehyde (35 mg, 0.37 mmol, 5 equiv.). After stirring the mixture for 2 h at room temperature, the reaction product was sequentially purified by column chromatography (silica gel, CH_2_Cl_2_/MeOH, 1:0 to 97:3) and preparative TLC (CH_2_Cl_2_/MeOH, 97:3) to afford 6 mg (0.011 mmol, yield 28%) of an amorphous yellow powder. ^1^H NMR (300 MHz, MeOD) δ 8.60 (1H, s, H-1′), 7.97 (2H, d, *J* = 8.1 Hz, H-3′, H-7′), 7.71 (2H, d, *J* = 8.3 Hz, H-4′, H-6′), 7.62 (1H, bd, *J* = 8.1 Hz, H-9), 7.39 (1H, bd, *J* = 8.2 Hz, H-12), 7.25 (1H, t, *J* = 7.3 Hz, H-11), 7.08 (1H, t, *J* = 7.5 Hz, H-10), 3.94 (1H, m, H-5), 3.85 (1H, dd, *J* = 13.3, 6.4 Hz, H-14a), 3.25 (1H, dd, *J* = 15.1, 8.5 Hz, H-6a), 3.02 (1H, dd, *J* = 14.7, 10.2 Hz, H-6b), 2.75–2.63* (5H, H-15, H-21b, –COOMe), 2.61–2.46* (5H, H-16, H-21a, *N*-Me), 2.35 (1H, t, *J* = 13.2 Hz, H-14b), 1.81 (1H, m, H-20), 1.41 (2H, m, H-19), 1.06 (3H, t, *J* = 7.3 Hz, H-18); ^13^C NMR (75 MHz, MeOD) δ 172.1 (–COOMe), 163.0 (C-3), 157.9 (C-1′), 140.1 (C-2′), 138.3 (C-13), 133.7 (C-2), 130.5 (C-8), 129.5 (C-3′, C-7′), 126.7 (C-4′), 126.6 (C-6′), 125.9 (C-11), 120.4 (C-10), 120.3 (C-9), 118.7 (C-7), 112.4 (C-12), 58.7 (C-5), 50.9 (–COOMe), 50.0–48.0* (C-16, C-21), 44.0 (C-20), 42.1 (*N*-Me), 31.7 (C-15), 25.6 (C-14), 24.2 (C-19), 21.4 (C-6), 11.3 (C-18) ppm. * Signals overlapped. ESIMS *m*/*z* 525 (M + H)^+^.

*3-[(4″-(trifluoromethoxy)benzylidene)hydrazineylidene]dregamine* (**11**). Obtained from reaction of compound **2** (15 mg, 0.041 mmol, 1 equiv.) with 4-trifluoromethoxybenzaldehyde (29 µL, 0.20 mmol, 5 equiv.). After stirring the mixture for 2 h at room temperature, the reaction product was sequentially purified by column chromatography (silica gel, CH_2_Cl_2_/MeOH, 1:0 to 97:3) and preparative TLC (CH_2_Cl_2_/MeOH, 97:3) to afford 15 mg (0.028 mmol, yield 68%) of an amorphous yellow powder. ^1^H NMR (300 MHz, MeOD) δ 8.55 (1H, s, H-1′), 7.87 (2H, d, *J* = 8.6, 2.3 Hz, H-4′, H-6′), 7.58 (1H, bd, *J* = 8.1 Hz, H-9), 7.40 (1H, bd, *J* = 8.2 Hz, H-12), 7.31 (2H, d, *J* = 8.3 Hz, H-3′, H-7′), 7.24 (1H, ddd, *J* = 8.1, 7.1, 1.0 Hz, H-11), 7.08 (1H, ddd, *J* = 7.9, 7.0, 0.9 Hz, H-10), 3.88–3.73* (2H, H-5, H-14a), 3.10 (1H, dd, *J* = 14.9, 8.2 Hz, H-6a), 2.80 (1H, dd, *J* = 14.6, 10.2 Hz, H-6b), 2.71–2.57* (5H, H-15, H-21b, –COOMe), 2.39–2.29* (5H, H-16, H-21a, *N*-Me), 2.15 (1H, t, *J* = 12.7 Hz, H-14b), 1.73 (1H, m, H-20), 1.37 (2H, m, H-19), 1.04 (3H, t, *J* = 7.3 Hz, H-18) ppm; ^13^C NMR (75 MHz, MeOD) δ 172.3 (–COOMe), 163.0 (C-3), 158.0 (C-1′), 151.8 (C-5′), 138.2 (C-13), 135.4 (C-2′), 133.7 (C-2), 130.8 (C-3′, C-7′), 130.5 (C-8), 125.7 (C-11), 122.1 (C-4′, C-6′), 120.3 (C-10), 120.3 (C-9), 118.8 (C-7), 112.4 (C-12), 58.5 (C-5), 50.9 (–COOMe), 50.0–48.0* (C-16, C-21), 44.1 (C-20), 42.1 (*N*-Me), 31.7 (C-15), 25.4 (C-14), 24.3 (C-19), 21.2 (C-6), 11.4 (C-18) ppm. * Signals overlapped. ESIMS *m*/*z* 541 (M + H)^+^.

*3-[(4″-(dimethylamino)benzylidene)hydrazineylidene]dregamine* (**12**). Obtained from reaction of compound **2** (20 mg, 0.054 mmol, 1 equiv.) with 4-dimethylaminobenzaldehyde (25 µL, 0.27 mmol, 5 equiv.). After stirring the mixture for 2 h at room temperature, the reaction product was sequentially purified by column chromatography (silica gel, CH_2_Cl_2_/MeOH, 1:0 to 97:3) and preparative TLC (CH_2_Cl_2_/MeOH, 97:3) to afford 7 mg (0.013 mmol, yield 26%) of an amorphous yellow powder. ^1^H NMR (300 MHz, MeOD) δ 8.45 (1H, s, H-1′), 7.67 (2H, d, *J* = 8.9 Hz, H-3′, H-7′), 7.57 (1H, bd, *J* = 8.0 Hz, H-9), 7.37 (1H, bd, *J* = 8.1 Hz, H-12), 7.21 (1H, ddd, *J* = 8.1, 7.0, 1.0 Hz, H-11), 7.05 (1H, ddd, *J* = 8.0, 6.9, 0.9 Hz, H-10), 6.75 (2H, d, *J* = 8.9 Hz, H-4′, H-6′), 3.99–3.88* (2H, H-5, H-14a), 3.20 (1H, dd, *J* = 14.9, 8.3 Hz, H-6a), 3.03 (6H, s, 5′-*N*-Me), 2.97 (1H, dd, *J* = 13.7, 9.4 Hz, H-6b), 2.74–2.62* (5H, H-15, H-21a, –COOMe), 2.60–2.45* (5H, H-16, H-21b, *N*-Me), 2.21 (1H, t, *J* = 12.8 Hz, H-14b), 1.80 (1H, m, H-20), 1.39 (2H, m, H-19), 1.08 (3H, t, *J* = 7.3 Hz, H-18) ppm; ^13^C NMR (75 MHz, MeOD) δ 172.0 (–COOMe), 160.5 (C-5′), 160.5 (C-3), 153.8 (C-1′), 138.0 (C-13), 134.3 (C-2), 130.9 (C-3′, C-7′), 130.6 (C-8), 125.3 (C-11), 123.9 (C-2′), 120.2 (C-10), 120.0 (C-9), 116.9 (C-7), 112.8 (C-4′, C-6′), 112.2 (C-12), 58.9 (C-5), 50.9 (–COOMe), 50.0–48.0* (C-16, C-21), 43.9 (C-20), 42.0 (*N*-Me), 40.3 (5′-*N*-Me), 31.5 (C-15), 25.1 (C-14), 24.2 (C-19), 21.3 (C-6), 11.4 (C-18) ppm. * Signals overlapped. ESIMS *m*/*z* 500 (M + H)^+^.

*3-[(4″-hydroxybenzylidene)hydrazineylidene]dregamine* (**13**). Obtained from reaction of compound **2** (29 mg, 0.078 mmol, 1 equiv.) with 4-hydroxybenzaldehyde (36 µL, 0.47 mmol, 6 equiv.). After stirring the mixture for 2 h at room temperature, the reaction product was sequentially purified by column chromatography (silica gel, CH_2_Cl_2_/MeOH, 1:0 to 97:3) and preparative TLC (CH_2_Cl_2_/MeOH, 97:3) to afford 6 mg (0.013 mmol, yield 16%) of an amorphous yellow powder. ^1^H NMR (300 MHz, MeOD) δ 8.44 (1H, s, H-1′), 7.67 (2H, d, *J* = 8.6 Hz, H-3′, H-7′), 7.59 (1H, bd, *J* = 8.0 Hz, H-9), 7.37 (1H, bd, *J* = 8.1 Hz, H-12), 7.21 (1H, ddd, *J* = 8.1, 7.0, 0.9 Hz, H-11), 7.06 (1H, ddd, *J* = 8.0, 7.0, 0.9 Hz, H-10), 6.84 (2H, d, *J* = 8.6 Hz, H-4′, H-6′), 3.97–3.84* (2H, H-5, H-14a), 3.19 (1H, dd, *J* = 14.4, 6.1 Hz, H-6a), 2.98 (1H, dd, *J* = 14.6, 10.3 Hz, H-6b), 2.76–2.63* (5H, H-15, H-21b, –COOMe), 2.56–2.42* (5H, H-16, *N*-Me, H-21a), 2.24 (1H, m, H-14b), 1.79 (1H, m, H-20), 1.38 (2H, m, H-19), 1.06 (3H, t, *J* = 7.3 Hz, H-18) ppm; ^13^C NMR (75 MHz, MeOD) δ 172.1 (–COOMe), 161.5 (C-5′), 161.2 (C-3), 159.7 (C-1′), 138.1 (C-13), 134.1 (C-2), 131.2 (C-3′, C-7′), 130.5 (C-8), 127.9 (C-2′), 125.5 (C-11), 120.3 (C-10), 120.1 (C-9), 117.5 (C-7), 116.6 (C-4′, C-6′), 112.2 (C-12), 58.8 (C-5), 50.9 (–COOMe), 50.0–48.0* (C-16, C-21), 44.0 (C-20), 42.1 (*N*-Me), 31.6 (C-15), 25.3 (C-14), 24.2 (C-19), 21.3 (C-6), 11.3 (C-18) ppm. * Signals overlapped. ESIMS *m*/*z* 473 (M + H)^+^.

*3-[(4″-methoxybenzylidene)hydrazineylidene]dregamine* (**14**). Obtained from reaction of compound **2** (25 mg, 0.068 mmol, 1 equiv.) with 4-methoxybenzaldehyde (50 µL, 0.41 mmol, 6 equiv.). After stirring the mixture for 2 h at room temperature, the reaction product was sequentially purified by column chromatography (silica gel, CH_2_Cl_2_/MeOH, 1:0 to 97:3) and preparative TLC (CH_2_Cl_2_/MeOH, 97:3) to afford 5 mg (0.009 mmol, yield 15%) of an amorphous yellow powder. ^1^H NMR (300 MHz, MeOD) δ 8.49 (1H, s, H-1′), 7.75 (2H, d, *J* = 8.8 Hz, H-3′, H-7′), 7.58 (1H, bd, *J* = 8.0 Hz, H-9), 7.37 (1H, bd, *J* = 8.2 Hz, H-12), 7.21 (1H, ddd, *J* = 8.1, 7.0, 0.9 Hz, H-11), 7.06 (1H, ddd, *J* = 8.0, 7.0, 1.0 Hz, H-10), 6.96 (2H, d, *J* = 8.8 Hz, H-4′, H-6′), 3.94–3.83* (5H, H-5, H-14a, 5′-OCH_3_), 3.20 (1H, dd, *J* = 15.3, 9.0 Hz, H-6a), 3.00 (1H, dd, *J* = 15.2, 10.4 Hz, H-6b), 2.74–2.63* (5H, H-15, H-21b, –COOMe), 2.54 (1H, m, H-16), 2.49–2.38* (4H, H-21a, *N*-Me), 2.28 (1H, t, *J* = 12.7 Hz, H-14b), 1.78 (1H, m, H-20), 1.39 (2H, m, H-19), 1.06 (3H, t, *J* = 7.3 Hz, H-18) ppm; ^13^C NMR (75 MHz, MeOD) δ 172.2 (–COOMe), 163.4 (C-5′), 161.6 (C-3), 159.3 (C-1′), 138.1 (C-13), 134.0 (C-2), 130.9 (C-3′, C-7′), 130.5 (C-8), 129.1 (C-2′), 125.5 (C-11), 120.2 (C-10), 120.1 (C-9), 117.9 (C-7), 115.2 (C-4′, C-6′), 112.2 (C-12), 58.8 (C-5), 55.9 (–OCH_3_), 50.9 (–COOMe), 50.0–48.0* (C-16, C-21), 44.1 (C-20), 42.2 (*N*-Me), 31.7 (C-15), 25.4 (C-14), 24.3 (C-19), 21.3 (C-6), 11.4 (C-18) ppm. * Signals overlapped. ESIMS *m*/*z* 487 (M + H)^+^.

*3-[(4″-(allyloxy)benzylidene)hydrazineylidene]dregamine* (**15**). Obtained from reaction of compound **2** (20 mg, 0.054 mmol, 1 equiv.) with 4-allyloxybenzaldehyde (39 µL, 0.27 mmol, 5 equiv.). After stirring the mixture for 2 h at room temperature, the reaction product was sequentially purified by column chromatography (silica gel, CH_2_Cl_2_/MeOH, 1:0 to 97:3) and preparative TLC (CH_2_Cl_2_/MeOH, 97:3) to afford 13 mg (0.025 mmol, yield 47%) of an amorphous yellow powder. ^1^H NMR (300 MHz, MeOD) δ 8.47 (1H, s, H-1′), 7.73 (2H, d, *J* = 8.7 Hz, H-3′, H-7′), 7.55 (1H, bd, *J* = 8.0 Hz, H-9), 7.37 (1H, bd, *J* = 8.1 Hz, H-12), 7.20 (1H, t, *J* = 7.5 Hz, H-11), 7.04 (1H, t, *J* = 7.5 Hz, H-10), 6.95 (2H, d, *J* = 8.7 Hz, H-4′, H-6′), 6.09 (1H, ddt, *J* = 17.1, 10.4, 5.2 Hz, H-2″), 5.44 (1H, dd, *J* = 17.3, 1.6 Hz, H-3″a), 5.29 (1H, dd, *J* = 10.6, 1.3 Hz, H-3″b), 4.59 (2H, dt, *J* = 5.1, 1.3 Hz, H-1″), 3.87–3.77* (2H, H-5, H-14a), 3.08 (1H, dd, *J* = 14.9, 8.2 Hz, H-6a), 2.81 (1H, dd, *J* = 14.6, 10.3 Hz, H-6b), 2.68–2.58* (5H, H-15, H-21b, –COOMe), 2.40–2.31* (5H, H-16, H-21a, *N*-Me), 2.12 (1H, t, *J* = 12.8 Hz, H-14b), 1.71 (1H, m, H-20), 1.35 (2H, m, H-19), 1.03 (3H, t, *J* = 7.3 Hz, H-18) ppm; ^13^C NMR (75 MHz, MeOD) δ 172.3 (–COOMe), 162.2 (C-5′), 161.8 (C-3), 159.4 (C-1′), 138.1 (C-13), 134.5 (C-2″), 134.0 (C-2), 130.9 (C-3′, C-7′), 130.5 (C-8), 129.2 (C-2′), 125.5 (C-11), 120.2 (C-10), 120.1 (C-9), 118.1 (C-3″), 117.8 (C-7), 115.9 (C-4′, C-6′), 112.3 (C-12), 69.8 (C-1″), 58.6 (C-5), 50.9 (–COOMe), 50.0–48.0* (C-16, C-21), 44.1 (C-20), 42.2 (*N*-Me), 31.6 (C-15), 25.3 (C-14), 24.3 (C-19), 21.2 (C-6), 11.4 (C-18) ppm. * Signals overlapped. ESIMS *m*/*z* 513 (M + H)^+^.

*3-[(4″-(benzyloxy)benzylidene)hydrazineylidene]dregamine* (**16**). Obtained from reaction of compound **2** (20 mg, 0.054 mmol, 1 equiv.) with 4-(benzyloxy)benzaldehyde (51 µL, 0.27 mmol, 5 equiv.). After stirring the mixture for 2 h at room temperature, the reaction product was sequentially purified by column chromatography (silica gel, CH_2_Cl_2_/MeOH, 1:0 to 97:3) and preparative TLC (CH_2_Cl_2_/MeOH, 97:3) to afford 16 mg (0.028 mmol, yield 52%) of an amorphous yellow powder. ^1^H NMR (300 MHz, MeOD) δ 8.47 (1H, s, H-1′), 7.72 (2H, d, *J* = 8.8, 2.3 Hz, H-3′, H-7′), 7.55 (1H, bd, *J* = 8.0 Hz, H-9), 7.45–7.30* (6H, H-12, H-3″, H-4″, H-5″, H-6″, H-7″), 7.21 (1H, ddd, *J* = 8.1, 7.0, 0.9 Hz, H-11), 7.06 (1H, ddd, *J* = 8.0, 6.9, 0.9 Hz, H-10), 6.98 (2H, d, *J* = 8.8, 2.4 Hz, H-4′, H-6′), 5.06 (2H, s, H-1″), 3.87–3.75* (2H, H-5, H-14a), 3.06 (1H, dd, *J* = 14.9, 8.2 Hz, H-6a), 2.77 (1H, dd, *J* = 14.6, 10.2 Hz, H-6b), 2.69–2.56* (5H, H-15, H-21b, –COOMe), 2.41–2.28* (5H, H-16, H-21a, *N*-Me), 2.07 (1H, t, *J* = 12.7 Hz, H-14b), 1.72 (1H, m, H-20), 1.33 (2H, m, H-19), 1.02 (3H, t, *J* = 7.2 Hz, H-18) ppm; ^13^C NMR (75 MHz, MeOD) δ 172.3 (–COOMe), 162.4 (C-5′), 161.8 (C-3), 159.4 (C-1′), 138.2 (C-2″), 138.1 (C-13), 134.0 (C-2), 131.0 (C-3′, C-7′), 130.5 (C-8), 129.5 (C-4″, C-6″), 129.3 (C-5″), 129.0 (C-2′), 128.6 (C-3″, C-7″), 125.5 (C-11), 120.3 (C-10), 120.1 (C-9), 118.1 (C-7), 116.1 (C-4′, C-6′), 112.3 (C-12), 71.0 (C-1″), 58.6 (C-5), 50.9 (–COOMe), 50.0–48.0* (C-16, C-21), 44.1 (C-20), 42.1 (*N*-Me), 31.6 (C-15), 25.3 (C-14), 24.3 (C-19), 21.2 (C-6), 11.4 (C-18) ppm. * Signals overlapped. ESIMS *m*/*z* 563 (M + H)^+^.

*3-[(3″,4″,5″-trimethoxybenzylidene)hydrazineylidene]dregamine* (**17**). Obtained from reaction of compound **2** (36 mg, 0.098 mmol, 1 equiv.) with 3,4,5-trimethoxybenzaldehyde (60 µL, 0.49 mmol, 5 equiv.). After stirring the mixture for 2 h at room temperature, the reaction product was sequentially purified by column chromatography (silica gel, CH_2_Cl_2_/MeOH, 1:0 to 97:3) and preparative TLC (CH_2_Cl_2_/MeOH, 97:3) to afford 8 mg (0.015 mmol, yield 15%) of an amorphous yellow powder. ^1^H NMR (300 MHz, MeOD) δ 8.48 (1H, s, H-1′), 7.58 (1H, bd, *J* = 7.9 Hz, H-9), 7.38 (1H, bd, *J* = 8.2 Hz, H-12), 7.22 (1H, t, *J* = 7.6 Hz, H-11), 7.17–7.13* (2H, H-3′, H-7′), 7.06 (1H, t, *J* = 7.6 Hz, H-10), 3.91–3.81* (11H, 4′-OCH_3_, 5′-OCH_3_, 6′-OCH_3_, H-5, H-14a), 3.18 (1H, dd, *J* = 14.9, 8.1 Hz, H-6a), 2.96 (1H, dd, *J* = 14.8, 10.4 Hz, H-6b), 2.79–2.59* (5H, –COOMe, H-15, H-21b), 2.51, (1H, m, H-16), 2.44 (3H, s, *N*-Me), 2.40–2.22* (2H, H-14b, H-21a), 1.78 (1H, m, H-20), 1.36 (2H, m, H-19), 1.08 (3H, t, *J* = 7.3 Hz, H-18) ppm; ^13^C NMR (75 MHz, MeOD) δ 172.5 (–COOMe), 162.5 (C-3), 159.8 (C-1′), 154.8 (C-4′, C-6′), 141.5 (C-5′), 138.2 (C-13), 133.9 (C-2), 132.2 (C-2′), 130.6 (C-8), 125.6 (C-11), 120.3 (C-10), 120.2 (C-9), 118.7 (C-7), 112.2 (C-12), 106.5 (C-3′, C-7′), 61.2 (5′-OCH_3_), 58.6 (C-5), 56.6 (4′-OCH_3_, 6′-OCH_3_), 50.8 (–COOMe), 50.0–48.0* (C-16, C-21), 44.1 (C-20), 42.3 (*N*-Me), 31.5 (C-15), 25.4 (C-14), 24.3 (C-19), 21.3 (C-6), 11.6 (C-18) ppm. * Signals overlapped. ESIMS *m*/*z* 547 (M + H)^+^.

*3-[(2″-naphthalenylmethylene)hydrazineylidene]dregamine* (**18**). Obtained from reaction of compound **2** (15 mg, 0.041 mmol, 1 equiv.) with 2-naphthaldehyde (27 µL, 0.20 mmol, 5 equiv.). After stirring the mixture for 2 h at room temperature, the reaction product was sequentially purified by column chromatography (silica gel, CH_2_Cl_2_/MeOH, 1:0 to 97:3) and preparative TLC (CH_2_Cl_2_/MeOH, 97:3) to afford 13 mg (0.026 mmol, yield 63%) of an amorphous yellow powder. ^1^H NMR (300 MHz, MeOD/CDCl_3_) δ 8.46 (1H, s, H-1′), 7.87 (1H, dd, *J* = 8.4, 1.4 Hz, H-3′), 7.79 (1H, s, H-7′), 7.67–7.54* (3H, H-9, H-1″, H-4″), 7.41 (1H, d, *J* = 8.2 Hz, H-4′), 7.37–7.25* (2H, H-2″, H-3″), 7.21 (1H, bd, *J* = 8.1 Hz, H-12), 7.07 (1H, t, *J* = 7.7 Hz, H-11), 6.91 (1H, t, *J* = 7.7 Hz, H-10), 3.80–3.70* (2H, H-5, H-14a), 3.01 (1H, dd, *J* = 14.8, 8.2 Hz, H-6a), 2.72 (1H, dd, *J* = 14.7, 10.3 Hz, H-6b), 2.62–2.45* (5H, H-15, H-21a, –COOMe), 2.34–2.23* (5H, H-16, H-21a, *N*-Me), 2.02 (1H, m, H-14b), 1.63 (1H, m, H-20), 1.23 (2H, m, H-19), 0.91 (3H, t, *J* = 7.3 Hz, H-18) ppm; ^13^C NMR (75 MHz, MeOD/CDCl_3_) δ 171.7 (-COOMe), 161.9 (C-3), 159.7 (C-1′), 137.67 (C-13), 135.6 (C-5′), 134.2 (C-6′), 133.5 (C-2), 133.5 (C-2′), 131.3 (C-7′), 130.0 (C-8), 129.3 (C-4″), 129.1 (C-2″), 128.5 (C-3″), 128.0 (C-4′), 127.3 (C-1″), 125.6 (C-11), 124.2 (C-3′), 120.2 (C-10), 119.8 (C-9), 117.7 (C-7), 112.0 (C-12), 58.3 (C-5), 50.8 (–COOMe), 50.0–48.0* (C-16, C-21), 43.5 (C-20), 42.0 (*N*-Me), 31.1 (C-15), 25.1 (C-14), 23.9 (C-19), 21.1 (C-6), 11.3 (C-18) ppm. *Signals overlapped. ESIMS *m*/*z* 507 (M + H)^+^.

*3-[((2″-hydroxy-1″naphthalenyl)methylene)hydrazineylidene]dregamine* (**19**). Obtained from reaction of compound **2** (15 mg, 0.041 mmol, 1 equiv.) with 2-hydroxy-1-naphthaldehyde (30 µL, 0.20 mmol, 5 equiv.). After stirring the mixture for 2 h at room temperature, the reaction product was sequentially purified by column chromatography (silica gel, CH_2_Cl_2_/MeOH, 1:0 to 97:3) and preparative TLC (CH_2_Cl_2_/MeOH, 97:3) to afford 12 mg (0.023 mmol, yield 56%) of an amorphous light orange powder. ^1^H NMR (300 MHz, MeOD/CDCl_3_) δ 9.55 (1H, s, H-1′), 8.00 (1H, d, *J* = 8.4 Hz, H-4″), 7.66–7.55* (3H, H-9, H-5′, H-1″), 7.48–7.37* (2H, H-12, H-3″), 7.34–7.25* (2H, H-11, H-2″), 7.13 (1H, m, H-10), 6.99 (1H, d, *J* = 9.0 Hz, H-4′), 3.88 (1H, m, H-5), 3.41 (1H, m, H-14a), 3.14 (1H, dd, *J* = 14.9, 8.2 Hz, H-6a), 2.76–2.63* (6H, H-6b, H-15, H-21b, -COOMe), 2.47–2.33* (4H, H-16, *N*-Me), 2.23–2.09* (2H, H-14b, H-21a), 1.75 (1H, m, H-20), 1.53 (1H, m, H-19a), 1.36 (1H, m, H-19b), 1.07 (3H, t, *J* = 7.3 Hz, H-18) ppm; ^13^C NMR (75 MHz, MeOD/CDCl_3_) δ 171.6 (-COOMe), 161.3 (C-3″), 160.5 (C-3), 160.3 (C-1′), 137.7 (C-13), 134.7 (C-1′’), 133.3 (C-6′), 133.2 (C-2), 129.9 (C-8), 129.6 (C-5′), 128.9 (C-7′), 128.2 (C-3′’), 125.8 (C-11), 124.2 (C-2″), 120.7 (C-4″), 120.4 (C-10), 119.9 (C-9), 119.1 (C-4′), 118.2 (C-7), 112.1 (C-12), 109.5 (C-2′), 58.1 (C-5), 50.9 (–COOMe), 50.0–48.0* (C-16, C-21), 43.9 (C-20), 42.0 (*N*-Me), 31.8 (C-15), 25.6 (C-14), 24.3 (C-19), 20.9 (C-6), 12.0 (C-18) ppm. * Signals overlapped. ESIMS *m*/*z* 523 (M + H)^+^.

*3-[((1H-indol-2-yl)methylene)hydrazineylidene]dregamine* (**20**). Obtained from reaction of compound **2** (30 mg, 0.081 mmol, 1 equiv.) with indole-2-carboxaldehyde (37 µL, 0.41 mmol, 5 equiv.). After stirring the mixture for 2 h at room temperature, the reaction product was sequentially purified by column chromatography (silica gel, CH_2_Cl_2_/MeOH, 1:0 to 96.5:3.5) and preparative TLC (CH_2_Cl_2_/MeOH, 96.5:3.5) to afford 10 mg (0.020 mmol, yield 25%) of an amorphous yellow powder. ^1^H NMR (300 MHz, MeOD) δ 8.50 (1H, s, H-1′), 7.63–7.54* (2H, H-9, H-3′), 7.44–7.34* (2H, H-12, H-6′), 7.25–7.17* (2H, H-11, H-4′), 7.09–7.02* (2H, H-10, H-5′), 6.84 (1H, s, H-2″), 3.93–3.79* (2H, H-5, H-14a), 3.21 (1H, dd, *J* = 14.8, 8.1 Hz, H-6a), 3.00 (1H, dd, *J* = 14.5, 10.3 Hz, H-6b), 2.70–2.64* (5H, H-15, H-21b, –COOMe), 2.50–2.43* (5H, H-16, H-21a, *N*-Me), 2.28 (1H, t, *J* = 12.7 Hz, H-14b), 1.75 (1H, m, H-20), 1.50 (1H, m, H-19a), 1.31 (1H, m, H-19b), 0.99 (3H, t, *J* = 7.4 Hz, H-18) ppm; ^13^C NMR (75 MHz, MeOD) δ 172.4 (–COOMe), 161.1 (C-3), 150.7 (C-1′), 139.5 (C-7′), 138.1 (C-13), 135.2 (C-2), 134.0 (C-2′), 130.6 (C-8), 129.7 (C-1″), 125.57 (C-11), 125.1 (C-4′), 122.3 (C-5′), 120.9 (C-3′), 120.2 (C-10), 120.1 (C-9), 118.2 (C-7), 112.6 (C-6′), 112.2 (C-12), 109.2 (C-2″), 58.7 (C-5), 50.9 (–COOMe), 50.0–48.0* (C-16, C-21), 44.4 (C-20), 42.3 (*N*-Me), 32.4 (C-15), 25.6 (C-14), 24.5 (C-19), 21.2 (C-6), 11.7 (C-18) ppm. * Signals overlapped. ESIMS *m*/*z* 496 (M + H)^+^.

*3-[((6-bromo-1H-indol-3-yl)methylene)hydrazineylidene]dregamine* (**21**). Obtained from reaction of compound **2** (37.5 mg, 0.102 mmol, 1 equiv.) with 6-bromoindole-3-carboxaldehyde (71 µL, 0.51 mmol, 5 equiv.). After stirring the mixture for 2 h at room temperature, the reaction product was sequentially purified by column chromatography (silica gel, CH_2_Cl_2_/MeOH, 1:0 to 24:1) and preparative TLC (CH_2_Cl_2_/MeOH, 24:1) to afford 20 mg (0.035 mmol, yield 34%) of an amorphous orange powder. ^1^H NMR (300 MHz, MeOD) δ 8.74 (1H, s, H-1′), 8.16 (1H, d, *J* = 8.4 Hz, H-3′), 7.59–7.51* (3H, H-9, H-6′, H-2″), 7.40 (1H, bd, *J* = 8.1 Hz, H-12), 7.23 (1H, ddd, *J* = 8.1, 7.0, 0.9 Hz, H-11), 7.13–7.02* (2H, H-10, H-4′), 4.10–3.98* (2H, H-5, H-14a), 3.11 (1H, dd, *J* = 14.8, 8.3 Hz, H-6a), 2.75–2.64* (6H, H-6b, H-15, H-16, –COOMe), 2.60 (1H, m, H-21b), 2.53 (3H, s, *N*-Me), 2.45 (1H, m, H-21a), 2.00 (1H, m, H-14b), 1.81 (1H, m, H-20), 1.46 (1H, m, H-19a), 1.39 (1H, m, H-19b), 1.04 (1H, t, *J* = 7.3 Hz, H-18) ppm; ^13^C NMR (75 MHz, MeOD) δ 171.2 (–COOMe), 159.5 (C-3), 156.8 (C-1′), 139.7 (C-7′), 137.9 (C-13), 134.7 (C-2), 133.2 (C-2″), 130.4 (C-8), 125.4 (C-11), 125.2 (C-2′, C-3′), 124.7 (C-4′), 120.4 (C-10), 120.0 (C-9), 117.3 (C-7), 115.6 (C-6′), 115.3 (C-5′), 114.9 (C-1″), 112.2 (C-12), 59.0 (C-5), 51.1 (–COOMe), 50.0–48.0* (C-16, C-21), 43.6 (C-20), 41.5 (*N*-Me), 31.6 (C-15), 25.1 (C-14), 24.3 (C-19), 21.3 (C-6), 12.0 (C-18) ppm. * Signals overlapped. ESIMS *m*/*z* 576 (M + H)^+^.

#### 3.2.2. General Preparation of Dregamine Semicarbazones **22–32**

Compound **2** (50 mg, 1 equiv.) and the suitable isocyanate (1.2–1.5 equiv.) were dissolved in tetrahydrofuran (THF, 2 mL) at room temperature. The mixture was stirred at 70 °C for 1 h under nitrogen atmosphere. After evaporating the solvent under vacuum, at 40 °C, the obtained residue was purified by flash chromatography, followed by preparative TLC.

*3-[(2″-(pentylcarbamoyl)hydrazineylidene]dregamine* (**22**). Obtained from reaction of compound **2** (50 mg, 0.136 mmol, 1 equiv.) with pentyl isocyanate (19 µL, 0.18 mmol, 1.3 equiv.). The mixture was stirred for 1 h at 70 °C and purified by column chromatography (silica gel, CH_2_Cl_2_/MeOH, 1:0 to 24:1) followed by preparative TLC (CH_2_Cl_2_/MeOH, 24:1) to afford 5 mg (0.010 mmol, yield 8%) of an amorphous light yellow powder. ^1^H NMR (300 MHz, CDCl_3_) δ 8.94 (1H, s, NH), 8.04 (1H, s, NH-2′), 7.57 (1H, bd, *J* = 7.9 Hz, H-9), 7.27 (1H, b, *J* = 7.8 Hz, H-12), 7.21 (1H, ddd, *J* = 8.0, 6.8, 1.1 Hz, H-11), 7.09 (1H, ddd, *J* = 8.0, 6.9, 1.2 Hz, H-10), 6.27 (1H, t, *J* = 5.9 Hz, NH-2″), 3.96 (1H, m, H-5), 3.40 (2H, m, H-1‴), 3.25 (1H, dd, *J* = 15.0, 8.2 Hz, H-6a), 3.11 (1H, dd, *J* = 14.8, 10.1 Hz, H-6b), 2.83–2.69* (3H, H-14a, H-15, H-16), 2.62 (3H, s, –COOMe), 2.57 (3H, s, *N*-Me), 2.61–2.53* (3H, H-14b, H-21), 1.83 (1H, m, H-20), 1.64 (2H, m, H-2‴), 1.50–1.27* (6H, H-19, H-3‴, H-4‴), 1.01 (3H, t, *J* = 7.4 Hz, H-18), 0.92 (3H, t, *J* = 7.0 Hz, H-5‴) ppm; ^13^C NMR (75 MHz, CDCl_3_) δ 171.0 (–COOMe), 156.6 (C-1″), 143.6 (C-3), 135.9 (C-13), 132.5 (C-8), 129.7 (C-2), 124.4 (C-11), 119.7 (C-10), 119.0 (C-9), 115.3 (C-7), 110.6 (C-12), 58.1 (C-5), 50.3 (–COOMe), 49.0 (C-16), 48.4 (C-21), 43.6 (C-20), 42.6 (*N*-Me), 40.2 (C-1‴), 31.3 (C-15), 30.2 (C-3‴), 29.3 (C-2‴), 24.0 (C-19), 23.3 (C-14), 22.6 (C-4‴), 20.0 (C-6), 14.2 (C-5‴), 12.2 (C-18) ppm. * Signals overlapped. ESIMS *m*/*z* 482 (M + H)^+^.

*3-[(2″-(tert-butylcarbamoyl)hydrazineylidene]dregamine* (**23**). Obtained from reaction of compound **2** (50 mg, 0.136 mmol, 1 equiv.) with *tert*-butyl isocyanate (23 µL, 0.20 mmol, 1.5 equiv.). The mixture was stirred for 1 h at 70 °C and purified by column chromatography (silica gel, CH_2_Cl_2_/MeOH, 1:0 to 19:1) followed by preparative TLC (CH_2_Cl_2_/MeOH, 19:1) to afford 8 mg (0.018 mmol, yield 13%) of an amorphous light yellow powder. ^1^H NMR (300 MHz, CDCl_3_) δ 8.69 (1H, s, NH), 7.58 (1H, s, NH-2′), 7.56 (1H, bd, *J* = 7.8 Hz, H-9), 7.27 (1H, d, *J* = 7.9 Hz, H-12), 7.20 (1H, ddd, *J* = 8.1, 6.8, 1.1 Hz, H-11), 7.08 (1H, ddd, *J* = 8.0, 6.8, 1.2 Hz, H-10), 5.97 (1H, s, NH-2″), 3.97 (1H, m, H-5), 3.26 (1H, dd, *J* = 15.0, 8.3 Hz, H-6a), 3.16 (1H, dd, *J* = 14.9, 10.0 Hz, H-6b), 2.81–2.66* (5H, H-14a, H-15, H-16, H-21), 2.63 (1H, m, H-14b), 2.61 (3H, s, –COOMe), 2.58 (3H, s, *N*-Me), 1.88 (1H, m, H-20), 1.47* (9H, s, H-2‴), 1.38 (2H, m, H-19), 1.03 (3H, t, *J* = 7.4 Hz, H-18) ppm; ^13^C NMR (75 MHz, CDCl_3_) δ 171.1 (–COOMe), 155.1 (C-1″), 143.1 (C-3), 135.8 (C-13), 132.3 (C-8), 129.7 (C-2), 124.4 (C-11), 119.7 (C-10), 119.0 (C-9), 115.3 (C-7), 110.6 (C-12), 58.1 (C-5), 50.7 (C-1‴), 50.3 (–COOMe), 48.9 (C-16), 48.6 (C-21), 43.5 (C-20), 42.6 (*N*-Me), 31.1 (C-15), 29.4 (C-2‴), 23.9 (C-19), 23.3 (C-14), 20.1 (C-6), 12.1 (C-18) ppm. * Signals overlapped. ESIMS *m*/*z* 468 (M + H)^+^.

*3-[(2″-(cyclohexylcarbamoyl)hydrazineylidene]dregamine* (**24**). Obtained from reaction of compound **2** (50 mg, 0.136 mmol, 1 equiv.) with cyclohexyl isocyanate (23 µL, 0.18 mmol, 1.3 equiv.). The mixture was stirred for 1 h at 70 °C and purified by column chromatography (silica gel, CH_2_Cl_2_/MeOH, 1:0 to 19:1) followed by preparative TLC (CH_2_Cl_2_/MeOH, 19:1) to afford 17 mg (0.033 mmol, yield 25%) of an amorphous light yellow powder. ^1^H NMR (300 MHz, CDCl_3_) δ 8.99 (1H, s, NH), 8.23 (1H, s, NH-2′), 7.56 (1H, bd, *J* = 8.0 Hz, H-9), 7.26 (1H, bd, *J* = 7.9 Hz, H-12), 7.19 (1H, ddd, *J* = 8.0, 6.9, 1.0 Hz, H-11), 7.07 (1H, ddd, *J* = 8.0, 6.9, 1.2 Hz, H-10), 6.15 (1H, d, *J* = 8.3 Hz, NH-2″), 3.95 (1H, m, H-5), 3.80 (1H, m, H-1‴), 3.24 (1H, dd, *J* = 15.0, 8.2 Hz, H-6a), 3.13 (1H, dd, *J* = 14.7, 10.1 Hz, H-6b), 2.89–2.70* (3H, H-14a, H-15, H-16), 2.61 (3H, s, –COOMe), 2.60–2.53* (2H, H-14b, H-21), 2.57 (3H, s, *N*-Me), 2.06 (2H, m, H-2‴), 1.88–1.73* (3H, H-20, H-3‴), 1.65 (1H, m, H-4‴a), 1.51 (1H, m, H-19a), 1.39–1.14* (6H, H-19b, H-6‴, H-5‴, H-4‴b), 1.01 (3H, t, *J* = 7.4 Hz, H-18) ppm; ^13^C NMR (75 MHz, CDCl_3_) δ 171.0 (–COOMe), 155.9 (C-1″), 143.8 (C-3), 135.9 (C-13), 132.5 (C-8), 129.6 (C-2), 124.3 (C-11), 119.5 (C-10), 118.9 (C-9), 115.1 (C-7), 110.6 (C-12), 58.0 (C-5), 50.2 (–COOMe), 49.1 (C-1‴), 49.0 (C-16), 48.4 (C-21), 43.7 (C-20), 42.6 (*N*-Me), 33.9 (C-2‴), 33.9 (C-6‴), 31.4 (C-15), 25.7 (C-4‴), 25.4 (C-3‴, C-5‴), 23.9 (C-19), 23.5 (C-14), 19.9 (C-6), 12.2 (C-18) ppm. * Signals overlapped. ESIMS *m*/*z* 494 (M + H)^+^.

*3-[(2″-(phenylcarbamoyl)hydrazineylidene]dregamine* (**25**). Obtained from reaction of compound **2** (50 mg, 0.136 mmol, 1 equiv.) with phenyl isocyanate (19 µL, 0.18 mmol, 1.3 equiv.). The mixture was stirred for 1 h at 70 °C and purified by column chromatography (silica gel, CH_2_Cl_2_/MeOH, 1:0 to 24:1) followed by preparative TLC (CH_2_Cl_2_/MeOH, 19:1) to afford 20 mg (0.041 mmol, yield 30%) of an amorphous yellow powder. ^1^H NMR (300 MHz, CDCl_3_) δ 9.43 (1H, s, NH), 8.82 (1H, s, NH-2′), 8.30 (1H, s, NH-2″), 7.61–7.50* (3H, H-9, H-2‴), 7.31–7.17* (4H, H-11, H-12, H-3‴), 7.14–6.99* (2H, H-10, H-4‴), 3.93 (1H, m, H-5), 3.22 (1H, dd, *J* = 14.8, 8.1 Hz, H-6a), 3.06 (1H, dd, *J* = 14.5, 10.2 Hz, H-6b), 2.90–2.66* (3H, H-14a, H-15, H-16), 2.61 (3H, s, –COOMe), 2.54 (3H, s, *N*-Me), 2.52–2.44* (3H, H-14b, H-21), 1.74 (1H, m, H-20), 1.30 (2H, m, H-19), 0.88 (3H, t, *J* = 7.3 Hz, H-18) ppm; ^13^C NMR (75 MHz, CDCl_3_) δ 171.4 (–COOMe)), 154.6 (C-1″), 146.2 (C-3), 138.2 (C-1‴), 136.4 (C-13), 132.5 (C-8), 129.7 (C-2), 129.0 (C-3‴, C-5‴), 124.6 (C-11), 123.6 (C-4‴), 120.3 (C-2‴, C-6‴), 119.7 (C-10), 119.1 (C-9), 116.0 (C-7), 111.0 (C-12), 58.2 (C-5), 50.5 (–COOMe), 49.1 (C-16), 48.4 (C-21), 43.7 (C-20), 42.7 (*N*-Me), 31.4 (C-15), 24.1 (C-19), 23.9 (C-14), 20.0 (C-6), 12.1 (C-18) ppm. * Signals overlapped. ESIMS *m*/*z* 488 (M + H)^+^.

*3-[(2″-(p-tolylcarbamoyl)hydrazineylidene]dregamine* (**26**). Obtained from reaction of compound **2** (50 mg, 0.136 mmol, 1 equiv.) with 4-tolyl isocyanate (18 µL, 0.18 mmol, 1.3 equiv.). The mixture was stirred for 1 h at 70 °C and purified by column chromatography (silica gel, CH_2_Cl_2_/MeOH, 1:0 to 24:1) followed by preparative TLC (CH_2_Cl_2_/MeOH, 19:1) to afford 14 mg (0.027 mmol, yield 21%) of an amorphous yellow powder. ^1^H NMR (300 MHz, CDCl_3_) δ 9.25 (1H, s, NH), 8.50 (1H, s, NH-2′), 8.11 (1H, s, NH-2″), 7.58 (1H, bd, *J* = 7.9 Hz, H-9), 7.44 (2H, d, *J* = 8.4 Hz, H-2‴, H-6‴), 7.28 (1H, bd, *J* = 8.0 Hz, H-12), 7.21 (1H, ddd, *J* = 8.0, 6.8, 0.9 Hz, H-11), 7.13–7.05* (3H, H-10, H-3‴, H-5‴), 3.95 (1H, m, H-5), 3.26 (1H, dd, *J* = 14.9, 8.1 Hz, H-6a), 3.12 (1H, dd, *J* = 14.9, 10.5 Hz, H-6b), 2.9–2.70* (3H, H-14a, H-15, H-16), 2.62 (3H, s, –COOMe), 2.57 (3H, s, *N*-Me), 2.56–2.45* (3H, H-14b, H-21), 2.30 (3H, s, 4‴-Me), 1.79 (1H, m, H-20), 1.34 (2H, m, H-19), 0.92 (3H, t, *J* = 7.3 Hz, H-18) ppm; ^13^C NMR (75 MHz, CDCl_3_) δ 171.2 (–COOMe), 154.4 (C-1″), 145.7 (C-3), 136.3 (C-13), 135.5 (C-1‴), 133.1 (C-4‴), 132.4 (C-8), 129.6 (C-2), 129.5 (C-3‴, C-5‴), 124.5 (C-11), 120.4 (C-2‴, C-6‴), 119.7 (C-10), 119.0 (C-9), 115.8 (C-7), 110.9 (C-12), 58.1 (C-5), 50.5 (–COOMe), 49.0 (C-16), 48.4 (C-21), 43.6 (C-20), 42.6 (*N*-Me), 31.3 (C-15), 23.9 (C-19), 23.9 (C-14), 20.9 (4‴-Me), 19.9 (C-6), 12.1 (C-18) ppm. * Signals overlapped. ESIMS *m*/*z* 502 (M + H)^+^.

*3-[2″-((4‴-methoxyphenyl)carbamoyl)hydrazineylidene]dregamine* (**27**). Obtained from reaction of compound **2** (50 mg, 0.136 mmol, 1 equiv.) with 4-methoxyphenyl isocyanate (21 µL, 0.16 mmol, 1.2 equiv.). The mixture was stirred for 1 h at 70 °C and purified by column chromatography (silica gel, CH_2_Cl_2_/MeOH, 1:0 to 97:3) followed by preparative TLC (CH_2_Cl_2_/MeOH, 19:1) to afford 25 mg (0.048 mmol, yield 36%) of an amorphous light orange powder. ^1^H NMR (300 MHz, CDCl_3_) δ 9.35 (1H, s, N-H), 8.55 (1H, s, NH-2′), 8.15 (1H, s, NH-2″), 7.57 (1H, bd, *J* = 7.9 Hz, H-9), 7.43 (2H, d, *J* = 9.0 Hz, H-2‴, H-6‴), 7.25–7.16* (2H, H-11, H-12), 7.08 (1H, ddd, *J* = 8.0, 6.4, 1.7 Hz, H-10), 6.83 (2H, d, *J* = 9.0 Hz, H-3‴, H-5‴), 3.96 (1H, m, H-5), 3.75 (3H, s, 4‴-OMe), 3.23 (1H, dd, *J* = 14.8, 8.1 Hz, H-6a), 3.05 (1H, dd, *J* = 14.5, 10.2 Hz, H-6b), 2.87–2.68* (3H, H-14a, H-15, H-16), 2.61 (3H, s, –COOMe), 2.55 (3H, s, *N*-Me), 2.53–2.41* (3H, H-14b, H-21), 1.77 (1H, m, H-20), 1.30 (2H, m, H-19), 0.92 (3H, t, *J* = 7.3 Hz, H-18); ^13^C NMR (75 MHz, CDCl_3_) δ 171.1 (–COOMe), 156.2 (C-4‴), 154.8 (C-1″), 145.4 (C-3), 136.2 (C-13), 132.5 (C-8), 131.1 (C-1‴), 129.5 (C-2), 124.5 (C-11), 122.7 (C-2‴, C-6‴), 119.6 (C-10), 118.9 (C-9), 115.5 (C-7), 114.2 (C-3‴, C-5‴), 110.9 (C-12), 58.1 (C-5), 55.5 (C-5′-OMe), 50.4 (–COOMe), 48.9 (C-16), 48.3 (C-21), 43.5 (C-20), 42.5 (*N*-Me), 31.2 (C-15), 23.8 (C-19), 23.6 (C-14), 20.0 (C-6), 12.0 (C-18) ppm. * Signals overlapped. ESIMS *m*/*z* 518 (M + H)^+^.

*3-[2″-((4‴-chlorophenyl)carbamoyl)hydrazineylidene]dregamine* (**28**). Obtained from reaction of compound **2** (50 mg, 0.136 mmol, 1 equiv.) with 4-chlorophenyl isocyanate (27 mg, 0.18 mmol, 1.3 equiv.). The mixture was stirred for 1 h at 70 °C and purified by column chromatography (silica gel, CH_2_Cl_2_/MeOH, 1:0 to 24:1) followed by preparative TLC (CH_2_Cl_2_/MeOH, 19:1) to afford 24 mg (0.045 mmol, yield 34%) of an amorphous yellow powder. ^1^H NMR (300 MHz, CDCl_3_) δ 9.48 (1H, s, NH), 8.66 (1H, s, NH-2′), 8.24 (1H, s, NH-2″), 7.58 (1H, bd, *J* = 8.0 Hz, H-9), 7.46 (2H, d, *J* = 8.9 Hz, H-2*‴*, H-6*‴*), 7.32 (1H, bd, *J* = 8.0 Hz, H-12), 7.26 (1H, m, H-11), 7.20 (2H, d, *J* = 8.8 Hz, H-3*‴*, H-5*‴*), 7.11 (1H, ddd, *J* = 8.0, 6.8, 1.0 Hz, H-10), 3.95 (1H, m, H-5), 3.25 (1H, dd, *J* = 14.8, 8.1 Hz, H-6a), 3.11 (1H, dd, *J* = 14.6, 10.3 Hz, H-6b), 2.88–2.67* (3H, H-14a, H-15, H-16), 2.59 (3H, s, –COOMe), 2.56 (3H, s, *N*-Me), 2.56–2.46* (3H, H-21, H-14b), 1.75 (1H, m, H-20), 1.25 (2H, m, H-19), 0.88 (3H, t, *J* = 7.4 Hz, H-18) ppm; ^13^C NMR (75 MHz, CDCl_3_) δ 171.4 (–COOMe), 154.6 (C-1″), 147.3 (C-3), 136.8 (C-1‴), 136.5 (C-13), 132.5 (C-8), 129.5 (C-2), 128.9 (C-3‴, C-5‴), 128.3 (C-4‴), 124.7 (C-11), 121.0 (C-2‴, C-6‴), 119.7 (C-10), 119.0 (C-9), 116.1 (C-7), 111.0 (C-12), 58.2 (C-5), 50.5 (–COOMe), 49.1 (C-16), 48.3 (C-21), 43.6 (C-20), 42.6 (*N*-Me), 31.4 (C-15), 24.2 (C-14), 23.8 (C-19), 19.9 (C-6), 12.0 (C-18) ppm. * Signals overlapped. ESIMS *m*/*z* 522 (M + H)^+^.

*3-[2″-((4‴-bromophenyl)carbamoyl)hydrazineylidene]dregamine* (**29**). Obtained from reaction of compound **2** (50 mg, 0.136 mmol, 1 equiv.) with 4-bromophenyl isocyanate (30 mg, 0.15 mmol, 1.1 equiv.). The mixture was stirred for 1 h at 70 °C and purified by column chromatography (silica gel, CH_2_Cl_2_/MeOH, 1:0 to 24:1) followed by preparative TLC (CH_2_Cl_2_/MeOH, 19:1) to afford 36 mg (0.063 mmol, yield 47%) of an amorphous yellow powder. ^1^H NMR (300 MHz, CDCl_3_) δ 9.62 (1H, s, NH), 8.76 (1H, s, NH-2′), 8.34 (1H, s, NH-2″), 7.57 (1H, bd, *J* = 8.0 Hz, H-9), 7.40 (2H, d, *J* = 8.9 Hz, H-2‴, H-6‴), 7.35–7.28* (3H, H-12, H-3‴, H-5‴), 7.22 (1H, ddd, *J* = 8.0, 6.9, 0.9 Hz, H-11), 7.09 (1H, ddd, 8.0, 7.2, 1.0 Hz, H-10), 3.93 (1H, m, H-5), 3.22 (1H, dd, *J* = 14.8, 8.1 Hz, H-6a), 3.04 (1H, dd, *J* = 14.5, 10.3 Hz, H-6b), 2.85–2.64* (3H, H-14a, H-15, H-16), 2.57 (3H, s, –COOMe), 2.54 (3H, s, *N*-Me), 2.51–2.37* (3H, H-14b, H-21), 1.71 (1H, m, H-20), 1.23 (2H, m, H-19), 0.86 (3H, t, *J* = 7.3 Hz, H-18) ppm; ^13^C NMR (75 MHz, CDCl_3_) δ 171.4 (–COOMe), 154.7 (C-1″), 147.1 (C-3), 137.3 (C-1‴), 136.5 (C-13), 132.5 (C-8), 131.8* (C-3‴, C-5‴), 129.5 (C-2), 124.6 (C-11), 121.5 (C-2‴, C-6‴), 119.7 (C-10), 119.0 (C-9), 115.9* (C-7, C-4‴), 111.0 (C-12), 58.1 (C-5), 50.5 (–COOMe), 49.0 (C-16), 48.2 (C-21), 43.5 (C-20), 42.5 (*N*-Me), 31.3 (C-15), 24.0 (C-14), 23.7 (C-19), 19.9 (C-6), 12.0 (C-18) ppm. * Signals overlapped. ESIMS *m*/*z* 568 (M + H)^+^.

*3-[2″-((4‴-nitrophenyl)carbamoyl)hydrazineylidene]dregamine* (**30**). Obtained from reaction of compound **2** (50 mg, 0.136 mmol, 1 equiv.) with 4-nitrophenyl isocyanate (29 mg, 0.18 mmol, 1.3 equiv.). The mixture was stirred for 1 h at 70 °C and purified by column chromatography (silica gel, CH_2_Cl_2_/MeOH, 1:0 to 24:1) followed by preparative TLC (CH_2_Cl_2_/MeOH, 19:1) to afford 17 mg (0.032 mmol, yield 24%) of an amorphous orange powder. ^1^H NMR (300 MHz, CDCl_3_) δ 9.49 (1H, s, NH), 8.69 (1H, s, NH-2′), 8.59 (1H, s, NH-2″), 8.07 (2H, d, *J* = 9.1 Hz, H-3‴, H-5‴), 7.68 (2H, d, *J* = 9.2 Hz, H-2‴, H-6‴), 7.60 (1H, bd, *J* = 8.0 Hz, H-9), 7.38 (1H, bd, *J* = 8.1 Hz, H-12), 7.27 (1H, ddd, *J* = 8.1, 6.9, 0.8 Hz, H-11), 7.13 (1H, ddd, J = 8.0, 6.8, 0.7 Hz, H-10), 3.95 (1H, m, H-5), 3.27 (1H, dd, *J* = 14.8, 8.1 Hz, H-6a), 3.13 (1H, dd, *J* = 14.7, 10.3 Hz, H-6b), 2.92–2.68* (3H, H-14a, H-15, H-16), 2.67–2.51* (9H, H-14b, H-21, –COOMe, *N*-Me), 1.77 (1H, m, H-20), 1.30 (2H, m, H-19), 0.91 (3H, t, *J* = 7.3 Hz, H-18) ppm; ^13^C NMR (75 MHz, CDCl_3_) δ 171.7 (–COOMe), 154.0 (C-1″), 149.1 (C-3), 144.3 (C-4‴), 142.8 (C-1‴), 136.7 (C-13), 132.1 (C-8), 129.5 (C-2), 125.1 (C-11), 125.0 (C-3‴, C-5‴), 119.9 (C-10), 119.2 (C-9), 118.5 (C-2‴, C-6‴), 116.9 (C-7), 111.1 (C-12), 58.2 (C-5), 50.6 (–COOMe), 49.1 (C-16), 48.3 (C-21), 43.6 (C-20), 42.6 (*N*-Me), 31.4 (C-15), 24.4 (C-14), 23.8 (C-19), 19.9 (C-6), 12.0 (C-18) ppm. * Signals overlapped. ESIMS *m*/*z* 533 (M + H)^+^.

*3-[2″-((4‴-(trifluoromethyl)phenyl)carbamoyl)hydrazineylidene]dregamine* (**31**). Obtained from reaction of compound **2** (50 mg, 0.136 mmol, 1 equiv.) with 4-(trifluoromethyl)phenyl isocyanate (25 µL, 0.18 mmol, 1.3 equiv.). The mixture was stirred for 1 h at 70 °C and purified by column chromatography (silica gel, CH_2_Cl_2_/MeOH, 1:0 to 24:1) followed by preparative TLC (CH_2_Cl_2_/MeOH, 19:1) to afford 33 mg (0.059 mmol, yield 44%) of an amorphous yellow powder. ^1^H NMR (300 MHz, CDCl_3_) δ 9.61 (1H, s, NH), 8.67 (1H, s, NH-2′), 8.33 (1H, s, NH-2″), 7.67–7.57* (3H, H-9, H-2‴, H-6‴), 7.47–7.38* (3H, H-12, H-3‴, H-5‴), 7.28 (1H, ddd, *J* = 8.1, 7.0, 1.0 Hz, H-11), 7.12 (1H, ddd, *J* = 7.9, 7.0, 0.9 Hz, H-10), 3.96 (1H, m, H-5), 3.28 (1H, dd, *J* = 14.8, 8.1 Hz, H-6a), 3.17 (1H, dd, *J* = 14.6, 10.3 Hz, H-6b), 2.92–2.76* (2H, H-14a, H-15), 2.73–2.62* (4H, H-14b, H-16, H-21), 2.60 (3H, s, –COOMe), 2.58 (3H, s, *N*-Me), 1.74 (1H, m, H-20), 1.27 (2H, m, H-19), 0.89 (3H, t, *J* = 7.4 Hz, H-18) ppm; ^13^C NMR (75 MHz, CDCl_3_) δ 171.7 (–COOMe), 154.5 (C-1″), 148.4 (C-3), 141.4 (C-1‴), 136.9 (C-13), 132.5 (C-8), 129.6 (C-2), 126.2 (C-3‴), 126.1 (C-5‴), 125.1 (C-4‴), 124.8 (C-11), 124.7 (4‴-CF_3_), 119.8 (C-10), 119.1 (C-9), 118.6 (C-2‴, C-6‴), 116.4 (C-7), 111.2 (C-12), 58.3 (C-5), 50.7 (–COOMe), 49.2 (C-16), 48.3 (C-21), 43.8 (C-20), 42.6 (*N*-Me), 31.6 (C-15), 24.6 (C-14), 23.4 (C-19), 20.0 (C-6), 12.0 (C-18) ppm. * Signals overlapped. ESIMS *m*/*z* 556 (M + H)^+^.

*3-[2″-((3-phenylpropyl)carbamoyl)hydrazineylidene]dregamine* (**32**). Obtained from reaction of compound **2** (50 mg, 0.136 mmol, 1 equiv.) with 3-phenylpropyl isocyanate (25 µL, 0.16 mmol, 1.2 equiv.). The mixture was stirred for 1 h at 70 °C and purified by column chromatography (silica gel, CH_2_Cl_2_/MeOH, 1:0 to 19:1) followed by preparative TLC (CH_2_Cl_2_/MeOH, 93:7) to afford 6 mg (0.011 mmol, yield 8%) of an amorphous light yellow powder. ^1^H NMR (300 MHz, CDCl_3_) δ 8.85 (1H, s, NH), 7.95 (1H, s, NH-2′), 7.57 (1H, bd, *J* = 8.0 Hz, H-9), 7.32–7.18* (7H, H-11, H-12, H-2^IV^, H-3^IV^, H-4^IV^, H-5^IV^, H-6^IV^), 7.10 (1H, ddd, *J* = 8.0, 6.8, 1.2 Hz, H-10), 6.23 (1H, t, *J* = 6.0 Hz, NH-2″), 4.00 (1H, m, H-5), 3.42 (2H, m, H-1‴), 3.27 (1H, dd, *J* = 14.9, 8.3 Hz, H-6a), 3.13 (1H, dd, *J* = 14.8, 10.2 Hz, H-6b), 2.84–2.68* (5H, H-14a, H-15, H-16, H-3‴), 2.66–2.61* (2H, H-14b, H-21), 2.60 (3H, s, -COOMe), 2.60 (3H, s, *N*-Me), 2.00 (2H, m, H-2‴), 1.87 (1H, m, H-20), 1.41 (2H, m, H-19), 1.02 (3H, t, *J* = 7.4 Hz, H-18) ppm; ^13^C NMR (75 MHz, CDCl_3_) δ 171.0 (–COOMe), 156.5 (C-1″), 143.6 (C-3), 141.8 (C-1^IV^), 135.9 (C-13), 132.4 (C-8), 129.6 (C-2), 128.5* (C-2^IV^, C-3^IV^, C-5^IV^, C-6^IV^), 126.1 (C-4^IV^), 124.5 (C-11), 119.7 (C-10), 119.0 (C-9), 115.3 (C-7), 110.6 (C-12), 58.1 (C-5), 50.4 (–COOMe), 48.7 (C-16), 48.5 (C-21), 43.4 (C-20), 42.5 (*N*-Me), 39.8 (C-1‴), 33.4 (C-3‴), 31.9 (C-2‴), 31.2 (C-15), 23.7 (C-19), 23.3 (C-14), 19.5 (C-6), 11.8 (C-18) ppm. * Signals overlapped. ESIMS *m*/*z* 530 (M + H)^+^.

### 3.3. Biological Assays

#### 3.3.1. Cell lines and Cultures

L5178Y mouse T-lymphoma cells (ECACC catalog no. 87111908, US FDA, Silver Spring, MD, USA) were transfected with the pHa *MDR1*/A retrovirus as previously reported [35]. The ABCB1 (P-gp) overexpressing cells were selected by culturing the infected cells with 60 ng/mL of colchicine (Sigma-Aldrich Chemie GmbH, Steinheim, Germany) to preserve the MDR phenotype. L5178Y (parental, PAR) mouse T-cell lymphoma cells and the human *ABCB1*-transfected subline were cultured in McCoy’s 5A supplemented with 10% heat-inactivated horse serum, 100 U/L L-glutamine, and 100 mg/L penicillin-streptomycin mixture, all obtained from Sigma-Aldrich. The mouse embryonic fibroblast (NIH 3T3) cell line was purchased from ATCC (CRL-1658) and was cultured in DMEM medium supplemented with 10% fetal bovine serum. These adherent cells were detached using 0.25% trypsin and 0.02% EDTA for 5 min at 37 °C. Before the antiproliferative assay using NIH 3T3 cell line, the cells were seeded in untreated 96-well flat bottomed microtiter plates, following a 4 h incubation period in a humidified atmosphere (5% CO_2_, 95% air) at 37 °C.

#### 3.3.2. Antiproliferative Assays

The antiproliferative effects of all compounds were tested in a range of decreasing concentrations (starting with 100 µM, then two-fold serial dilution), using mouse lymphoma cells as experimental model, in 96-well flat bottomed microtiter plates. Cisplatin (TEVA Pharmaceutical Company, Petah Tikva, Israel) used in cell lines served as positive control. First, the compounds were diluted in 100 µL of medium. The maximum tested concentration of each compound was 100 µM. Then, 6 × 10^3^ cells in 100 µL of medium were added to each well, with the exception of the medium control wells. The culture plates were initially incubated at 37 °C for 72 h, and at the end of the incubation period, 20 µL of MTT (thiazolyl blue tetrazolium bromide; Sigma-Aldrich Chemie GmbH, Steinheim) solution of 5 mg/mL in phosphate-buffered saline (PBS) was added to each well and incubated for another 4 h. Then, 100 µL of 10% SDS (sodium dodecyl sulfate, Sigma) solution (10% in 0.01 N HCl) was added to each well, and the plates were further incubated overnight at 37 °C. Cell growth was determined by measuring the optical density (OD) at 540/630 nm with a Multiscan EX ELISA reader (ThermoLabsSystems, Cheshire, WA, USA). The percentage of inhibition of cell growth was determined according to Equation (2). All experiments were performed in triplicate. The results were expressed as the mean IC_50_ ± SD, and the IC_50_ values were obtained by best-fitting the dose-dependent inhibition curves in GraphPad Prism 5 software [29]. Only data from analysis with R^2^ > 0.90 were presented.

(2) $$100-\left[\frac{O_{\mathrm{sample}}-{\mathrm{OD}}_{\mathrm{mediumcontrol}}}{{\mathrm{OD}}_{\mathrm{cellcontrol}}-{\mathrm{OD}}_{\mathrm{medium}\;\mathrm{control}}}\right]\times 100$$

The assay was performed according to the previously applied experimental settings [13].

The statistical analysis of data was performed using GraphPad Prism 5 software, applying the two-tailed *t*-test, and *p*-values of <0.05 were considered significant.

#### 3.3.3. Rhodamine-123 Accumulation Assay

PAR and MDR mouse T-lymphoma cells were used in a density of 2 × 10^6^ cells/mL, resuspended in serum-free McCoy’s 5A medium and distributed in 500 µL aliquots. The compounds were pipetted at two concentrations (0.2 or 2 µM), and verapamil (positive control, EGIS Pharmaceuticals PLC, Budapest, Hungary) was applied at 20 µM. DMSO at 2% was also added as solvent control. The samples were incubated for 10 min at room temperature, after which 10 µL of rhodamine-123 (5.2 µM final concentration) was measured to the samples. After 20 min of incubation at 37 °C, the samples were washed twice, resuspended in 1 mL of PBS, and analyzed by flow cytometry (Partec CyFlow Space Instrument, Partec GmbH, Münster, Germany). The resulting histograms were evaluated regarding mean fluorescence intensity (FL-1), standard deviation, both forward scatter (FSC) and side scatter (SSC) parameters, and the peak channel of 20,000 individual cells belonging to the total and the gated populations (Supporting Information). The fluorescence activity ratio was calculated on the basis of the quotient between FL-1 of treated/untreated resistant cell line (*ABCB1*-transfected mouse lymphoma cells) and the respective treated/untreated sensitive cell line (PAR mouse lymphoma cells), according to Equation (3).

(3) $$\mathrm{FAR}=\frac{\frac{\mathrm{FL}1{\mathrm{MDR}}_{\mathrm{treated}}}{\mathrm{FL}1{\mathrm{MDR}}_{\mathrm{untreated}}}}{\frac{\mathrm{FL}1{\mathrm{PAR}}_{\mathrm{treated}}}{\mathrm{FL}1{\mathrm{PAR}}_{\mathrm{untreated}}}}$$

#### 3.3.4. Drug Combination Assay

Doxorubicin (2 mg/mL, Teva Pharmaceuticals, Budapest, Hungary) was serially diluted horizontally in 100 µL as previously described, starting with 8.6 µM. The resistance modifier was subsequently diluted vertically in 50 µL; the starting concentration was determined based on the IC_50_. After resuspending the cells in culture medium, they were distributed into each well in 50 µL containing 6 × 10^3^ cells, with the exception of the medium control wells, to a final volume of 200 µL per well. The checkerboard plates were kept for 72 h at 37 °C in a CO_2_ incubator, and at the end of the incubation period, the cell growth was determined by MTT staining method, as described earlier. Drug interactions were evaluated using Calcusyn software [36]. Each dose–response curve (for individual agents as well as combinations) was fit to a linear model using the median effect equation in order to obtain the median effect value (corresponding to the IC_50_) and slope (m) [28,29]. The goodness-of-fit was assessed using the linear correlation coefficient, *r*, and only data from analysis with *r* > 0.90 were presented. The extent of interaction between drugs was expressed using the combination index, in which a CI value close to 1 indicates additivity, while a CI < 1 is defined as synergy and a CI > 1 as antagonism.

#### 3.3.5. ATPase Activity Assay

The P-glycoprotein ATPase activity was determined using the Pgp-Glo^TM^ Assay Systems Promega kit according to the manufacturer’s instructions [30]. Briefly, 20 µL of recombinant human P-gp membranes (1.25 mg/mL), expressing high levels of human P-gp, were incubated in 20 µL of Pgp-Glo^TM^ assay buffer for 5 min at 37 °C. Compounds were tested at 25 µM sodium orthovanadate (Na_3_VO_4_, 0.25 mM) as an inhibitor control, verapamil as a substrate control (0.5 mM), and DMSO at 2% as solvent control. The reaction was initiated by adding 10 µL of 25 mM MgATP and incubated at 37 °C for 40 min. The ATPase reaction was stopped, and after adding 50 µL of ATP Detection Reagent, the samples were incubated at room temperature for 20 min. The luciferase-generated luminescent signal emitted was measured in a CLARIOstar Plus plate reader (BMG Labtech, UK) at 580 nm. The % of basal P-gp activity was calculated through the ratio between the luminescence measured of the P-gp ATPase activity of each compound and the basal activity according to Equation (4).

(4) $$\%\;\mathrm{basal}\;P-\mathrm{gp}\;\mathrm{activity}\;=\;\frac{{\mathrm{Lum}}_{{\mathrm{Na}}_{3}{\mathrm{VO}}_{4}}-{\mathrm{Lum}}_{\mathrm{treated}}}{{\mathrm{Lum}}_{{\mathrm{Na}}_{3}{\mathrm{VO}}_{4}}-{\mathrm{Lum}}_{\mathrm{untreated}}}\times 100\%$$

### 3.4. Computational Studies

#### 3.4.1. Molecular Docking

Compounds were previously drawn, energy minimized (default force-field, adjustment of hydrogens and lone pairs), and exported as mol2 files in MOE v2019.01 program. PDBQT files were created with AutoDockTools v1.5.6rc2 for further use in AutoDock VINA 1.1.2 docking software. The murine P-glycoprotein structure (ID: 3G5U) was obtained from the Protein Data Bank (PDB), in which the small linker sequence was added according to Ferreira et al. [10]. The binding location was defined by a docking box, including the whole internal cavity as defined previously by Ferreira et al. [10]. AutoDock VINA was used to generate docking poses, from which the 20 top-ranked were visualized to determine at which different drug binding sites (M, R or H) the pose was located. The ability to modulate efflux by cross-interacting with both P-gp halves (CIc) and, therefore, hinder conformation changes leading to efflux was performed using the top-ranked docking pose at the M-site (box centered at the M-site with dimensions xyz of 35.25 Å × 30.75 Å × 29.25 Å. [10] The corresponding CIc was calculated by the ratio between the nonbonded interactions at the *N*-terminal and *C*-terminal halves (with LIGPLOT). MDR-reversal capability of derivatives and inhibition category was thereafter evaluated considering the total number of contacts, binding energies, binding site, and CIc values.

#### 3.4.2. QSAR Modeling

For each molecule, an extensive database of molecular descriptors (constitutional, topological, and geometrical) was generated from E-DRAGON [37] (1665 descriptors), PaDEL [38] (105 descriptors), and MOE (429 descriptors) software programs. Constitutional descriptors give information about the number of atoms and bonds within each molecule, whereas topological and geometrical descriptors make reference to the composition and spatial arrangement of a certain compound.

Thereafter, experimental FAR values obtained were added to the dataset, and the most relevant combination of molecular descriptors in each database was selected by the CfsSubsetEval attribute evaluator [39] within the WEKA software [40] using the BestFirst algorithm as the search method. In the end, the molecular descriptors were reduced to 21 (17 and 4 for E-DRAGON and MOE subsets, respectively), and the corresponding QSAR models were built in WEKA software [40]. QSAR models from each subset were obtained by univariate linear regression to identify which molecular descriptors perform better. In parallel, accurate QSAR models were also built using the machine-learning methods: a support vector machine by the SMOReg approach, using RegSMOImproved as the learning algorithm [41], and an artificial neural network approach (MLPRegressor), by training a multilayer perceptron model with a single hidden layer using WEKA’s optimization class, minimizing the squared error plus a quadratic penalty with the Broyden–Fletcher–Goldfarb–Shanno (BFGS) method [42]. In both cases, the number of descriptors used was reduced as small as possible, leading to models of easier interpretation while keeping a good predictive result. The robustness of the created models was assessed by a *k*-fold cross-validation correlation coefficient (tenfold, q^2^) and their predictive power (R^2^_pred_) by splitting the dataset into training and test sets (75:25). Other parameters as the mean absolute error (MAE) and root mean squared error (RMSE) were calculated to reinforce the reliability of the model.

## 4. Conclusions

As ongoing research on the optimization of monoterpene indole alkaloids as MDR-reversers, this work was focused on the generation of new analogs by modifying the ketone group of dregamine (**1**), yielding 19 azines (**3**–**21**) and 11 semicarbazones (**22**–**32**). Among the 30 new derivatives (**3**–**32**), most showed remarkable enhancement in P-gp inhibitory activity. In the transport assay, the strongest MDR reversal compounds were those having azine substituents attached to the indole alkaloid scaffold, containing trimethoxyphenyl (**17**) or naphthyl moieties (**18**, **19**), being, at a 10-fold lower concentration, up to 20-fold more active than the reference inhibitor verapamil. Moreover, most of the azine derivatives bearing aromatic substituents exhibited, simultaneously, a significant and MDR-selective antiproliferative effect in P-gp-overexpressing cells (**5**–**9**, **11**, **12**, **14**–**18**), thus showing a dual role in reversing P-gp-mediated MDR. The results obtained in the functional assay were substantiated by those found in a combination assay, where all derivatives (**3**–**32**) displayed synergistic interactions with doxorubicin. In the ATPase activity assay, it was observed that the selected compounds **17**, **18**, **20,** and **29** showed to behave as inhibitors, whereas compound **12** stimulated the ATPase activity, acting, possibly, as a competitive inhibitor.

In silico studies revealed that despite most compounds having their best pose at the modulator binding site (M-site), only a few azine derivatives (**4**, **6**, **16**, and **19**) showed to interact strongly with P-gp together with strong cross-interaction capability, acting as non-competitive inhibitors. Conversely, compounds **10**, **11**, and **17** displayed a high affinity with the substrate-binding sites (R- and H-sites), and the best M-pose showed weak cross-interactions with both P-gp halves and thus was considered as a prototype of competitive inhibitors. A QSAR model was built, and the results showed that compounds having more lipophilic and bulkier substituents may affect, in a positive manner, the P-gp inhibitory activity.

## Figures and Tables

**Table 1:** IC_50_ values of compounds (**1**–**32**) and cisplatin, obtained in the antiproliferative assay, against sensitive (PAR), *ABCB1*-transfected (MDR) L5178Y mouse lymphoma cells, and non-cancer mouse embryonic fibroblasts (NIH/3T3).

Compound	IC_50_ (µM) ^1^		RR ^2^	IC_50_ (µM)	SI ^3^	
	PAR (A)	MDR (B)		NIH/3T3 (C)	C/A	C/B
**1**	37.21 ± 4.99 ^†^	22.97 ± 0.48 ^§^	0.62	>100 ^§^	>2.69	***>4.35***
**3**	53.13 ± 3.16 ^§^	31.50 ± 1.84 ^§^	0.59	>100 ^§^	>1.88	***>3.17***
**4**	19.05 ± 0.76 ^§^	10.86 ± 1.34 ^†^	0.57	34.94 ± 5.63 ^†^	1.83	***3.22***
**5**	14.80 ± 1.23 ^†^	5.96 ± 0.26 ^†^	**0.40**	12.66 ± 2.29 *	0.86	2.12
**6**	11.93 ± 1.09 ^†^	8.72 ± 0.52 ^§^	0.73	14.44 ± 1.36 ^†^	1.21	1.65
**7**	13.05 ± 0.74 ^§^	4.62 ± 0.14 *	**0.35**	12.05 ± 1.40 ^†^	0.92	2.61
**8**	8.84 ± 0.76 *	7.47 ± 0.17 ^†^	0.85	11.67 ± 2.07 *	1.32	1.56
**9**	14.46 ± 0.84 ^§^	6.57 ± 0.12 ^†^	**0.45**	9.67 ± 0.94 ^†^	0.67	1.47
**10**	20.73 ± 0.80 ^§^	11.69 ± 0.67 ^§^	0.56	12.42 ± 1.58 ^†^	0.60	1.06
**11**	23.66 ± 0.64 ^§^	8.40 ± 0.52 ^§^	**0.36**	13.47 ± 1.14 ^†^	0.57	1.60
**12**	12.84 ± 0.16 ^†^	7.42 ± 0.48 ^§^	0.58	14.17 ± 0.59 ^§^	1.10	1.91
**13**	26.40 ± 0.53 ^§^	7.75 ± 0.55 ^§^	**0.29**	30.50 ± 0.95 ^§^	1.16	***3.94***
**14**	18.08 ± 2.69 *	8.64 ± 0.67 ^§^	**0.48**	19.73 ± 0.41 ^§^	1.09	2.28
**15**	12.56 ± 1.06 ^†^	6.41 ± 0.33 ^†^	0.51	14.86 ± 0.72 ^§^	1.18	2.32
**16**	8.93 ± 0.75 *	5.86 ± 0.54 ^†^	0.66	18.26 ± 1.30 ^†^	2.04	***3.12***
**17**	9.07 ± 0.67 *	7.90 ± 0.44 ^§^	0.87	85.21 ± 1.62 ^§^	**9.39**	**10.79**
**18**	11.31 ± 0.84 ^†^	9.58 ± 0.74 ^†^	0.85	11.73 ± 1.53 ^†^	1.04	1.22
**19**	12.90 ± 0.35 ^†^	12.91 ± 0.35 ^§^	1.00	17.48 ± 1.03 ^†^	1.35	1.35
**20**	8.72 ± 0.53 *	8.73 ± 0.53 ^§^	1.00	13.75 ± 0.69 ^†^	1.58	1.57
**21**	13.62 ± 0.03 ^†^	12.91 ± 1.57 ^†^	0.95	14.78 ± 2.30 ^†^	1.08	1.14
**22**	5.43 ± 0.38 *	4.28 ± 0.25 *	0.79	26.71 ± 0.63 ^§^	***4.92***	**6.24**
**23**	26.27 ± 0.50 ^§^	12.33 ± 1.80 ^†^	**0.47**	>100 ^§^	***>3.81***	**>8.11**
**24**	12.85 ± 0.12 ^†^	5.69 ± 0.71 *	**0.44**	26.64 ± 0.31 ^§^	2.07	***4.68***
**25**	12.95 ± 0.04 ^†^	10.77 ± 0.71 ^§^	0.83	27.57 ± 0.81 ^§^	2.13	2.56
**26**	9.41 ± 0.13 *	8.22 ± 1.10 ^†^	0.87	25.89 ± 0.81 ^§^	2.75	***3.15***
**27**	6.88 ± 0.35 *	6.37 ± 0.83 ^†^	0.92	40.23 ± 3.49 ^†^	***5.84***	**6.32**
**28**	6.22 ± 0.59 *	5.21 ± 0.09 *	0.84	12.79 ± 0.95 ^†^	2.06	2.45
**29**	6.56 ± 0.04 *	7.40 ± 0.20 ^†^	1.13	13.03 ± 0.40 ^§^	1.99	1.76
**30**	6.04 ± 0.36 *	4.83 ± 0.05 *	0.80	13.99 ± 0.92 ^†^	2.32	2.90
**31**	6.97 ± 0.12 *	6.48 ± 0.25 ^†^	0.93	6.70 ± 0.06 ^§^	0.96	1.03
**32**	6.82 ± 0.64 *	4.46 ± 1.38 *	0.65	9.51 ± 0.52 *	1.39	2.13
**Cisplatin**	8.16 ± 1.70	3.29 ± 0.52	0.40	1.06 ± 0.02	0.13	0.32

^1^ IC_50_ parameter is given as the mean of three independent experiments * *p* < 0.05, ^†^ *p* < 0.01, ^§^ *p* < 0.001; ^2^ RR (relative resistance ratio) = IC_50_MDR/IC_50_PAR; compounds with CS effect (RR ≤ 0.5) are highlighted in bold; ^3^ SI: Selectivity Index; SI < 1 values denote lack of selectivity, 1 < SI < 3 mean a slight selectivity and 3 < SI < 6 values indicate moderate selectivity and are marked with bold and italics; whereas values of SI > 6 indicate that the compounds are strongly selective and are highlighted in bold [19].

**Table 2:** P-gp inhibitory activity of compounds **1** and **3**–**21** on human *ABCB1*-transfected L5178Y mouse lymphoma cells.

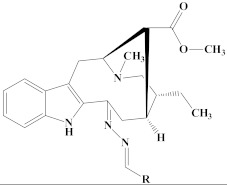							
**Compound**	**R**	**Conc. (µM)**	**FAR ^1^**	**Compound**	**R**	**Conc. (µM)**	**FAR ^1^**
**1**	Dregamine	0.2	1.10	**12**	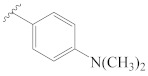	0.2	11.57
		2	1.03			2	26.51
**3**	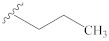	0.2	0.81	**13**	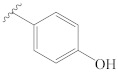	0.2	1.31
		2	1.22			2	5.25
**4**	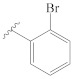	0.2	4.99	**14**	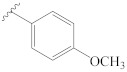	0.2	2.37
		2	33.93			2	16.63
**5**	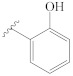	0.2	3.16	**15**	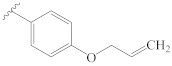	0.2	7.08
		2	22.72			2	26.51
**6**	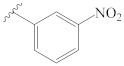	0.2	5.78	**16**	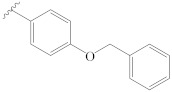	0.2	3.50
		2	24.34			2	104.59
**7**	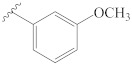	0.2	7.54	**17**	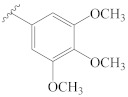	0.2	11.99
		2	25.06			2	100.07
**8**	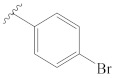	0.2	6.82	**18**	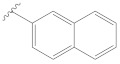	0.2	30.74
		2	23.61			2	126.58
**9**	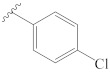	0.2	6.55	**19**	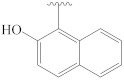	0.2	16.31
		2	22.60			2	128.48
**10**	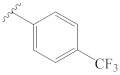	0.2	1.70	**20**	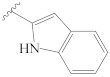	0.2	6.07
		2	15.69			2	106.19
**11**	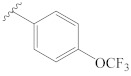	0.2	2.70	**21**	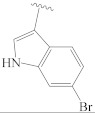	0.2	3.00
		2	15.88			2	95.56
Verapamil ^2^	-	20	6.34	DMSO ^3^	-	2%	0.79

^1^FAR (fluorescence activity ratio) = (FL-1_MDRtreated_/FL-1_MDRuntreated_)/(FL-1_PARtreated_/FL-1_PARuntreated_); ^2,3^ Verapamil and DMSO were used as positive and negative control, respectively.

**Table 3:** P-gp inhibitory activity of compounds **22**–**32** on human *ABCB1*-transfected L5178Y mouse lymphoma cells.

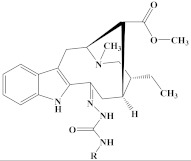			
**Compound**	**R**	**Conc. (µM)**	**FAR ^1^**
**22**	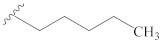	0.2	1.36
		2	2.93
**23**	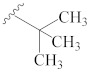	0.2	0.96
		2	1.10
**24**	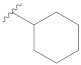	0.2	1.06
		2	1.82
**25**	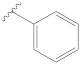	0.2	1.26
		2	5.93
**26**	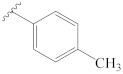	0.2	1.00
		2	8.77
**27**	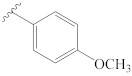	0.2	0.81
		2	3.20
**28**	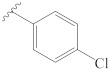	0.2	0.79
		2	7.80
**29**	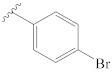	0.2	1.16
		2	7.44
**30**	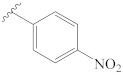	0.2	1.06
		2	7.29
**31**	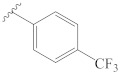	0.2	0.73
		2	4.80
**32**	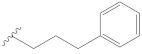	0.2	1.41
		2	6.60
Verapamil ^2^	-	20	6.34
DMSO ^3^	-	2%	0.79

^1^ FAR (fluorescence activity ratio) = (FL-1_MDRtreated_/FL-1_MDRuntreated_)/(FL-1_PARtreated_/FL-1_PARuntreated_); ^2,3^ Verapamil and DMSO were used as positive and negative control, respectively.

**Table 4:** Molecular docking results.

Cpd.	FAR	ΔG/kcal.mol^−1^ (n Poses)			Best	Affinity	CIc *
		M-Site	R-Site	H-Site	M-Pose		
**1**	1.03	−9.1 (9)	−8.3 (11)	− (0)	1	Moderate	Weak
**3**	1.22	−8.8 (12)	−9.2 (6)	−7.7 (2)	2	Moderate	Moderate
**4**	33.93	−10.5 (8)	−9.5 (5)	−9.2 (7)	1	Strong	Strong
**5**	22.72	−9.7 (16)	−9.0 (2)	−9.0 (2)	1	Moderate	Weak
**6**	24.34	−10.0 (12)	−9.5 (1)	−8.5 (7)	1	Strong	Strong
**7**	25.06	−9.2 (11)	−8.7 (5)	−8.2 (4)	1	Moderate	Strong
**8**	23.61	−10.2 (8)	−8.4 (2)	−8.9 (10)	1	Strong	Moderate
**9**	22.60	−10.2 (8)	−8.5 (2)	−8.9 (10)	1	Strong	Weak
**10**	15.69	−9.6(8)	−11.0 (7)	−9.2 (5)	5	Strong	Weak
**11**	15.88	−9.7 (8)	−10.7 (5)	−8.6 (7)	4	Strong	Weak
**12**	26.51	−9.5 (10)	−9.7 (4)	−8.7 (6)	2	Moderate	Moderate
**13**	5.25	−9.6 (12)	−9.1 (1)	−9.1 (7)	1	Moderate	Weak
**14**	16.63	−9.4 (9)	−9.5 (4)	−9.1 (7)	2	Moderate	Weak
**15**	26.51	−9.3 (11)	−8.3 (3)	−8.4 (6)	1	Moderate	Strong
**16**	104.59	−11.5 (13)	−9.4 (1)	−9.7 (6)	1	Strong	Strong
**17**	100.07	−8.7 (9)	−9.2 (3)	−9.0 (8)	5	Moderate	Weak
**18**	126.58	−10.5 (10)	−10.7 (7)	−9.9 (3)	4	Strong	Moderate
**19**	128.48	−10.0 (12)	−9.8 (3)	−9.4 (5)	1	Strong	Strong
**20**	106.19	−9.8 (10)	−8.8 (1)	−9.0 (9)	1	Moderate	Strong
**21**	95.56	−9.5 (14)	− (0)	−9.5 (6)	1	Moderate	Strong
**22**	2.93	−8.5 (13)	−9.2 (6)	−7.5 (1)	2	Moderate	Moderate
**23**	1.10	−9.8 (9)	−9.0 (7)	−8.6 (4)	1	Moderate	Strong
**24**	1.82	−8.7 (9)	−8.7 (5)	−8.5 (6)	1	Weak	Weak
**25**	5.93	−9.1 (12)	−9.5 (6)	−9.0 (2)	2	Moderate	Strong
**26**	8.77	−9.9 (12)	−8.9 (1)	−8.9 (7)	1	Moderate	Weak
**27**	3.20	−9.1 (10)	−10.0 (3)	−8.9 (7)	2	Strong	Strong
**28**	7.80	−10.2 (10)	−9.4 (6)	−9.0 (4)	1	Strong	Weak
**29**	7.44	−9.4 (13)	−9.0 (3)	−8.9 (4)	1	Moderate	Weak
**30**	7.29	−10.1 (10)	−10.0 (3)	−9.3 (7)	1	Strong	Moderate
**31**	4.80	−10.2 (4)	−10.2 (4)	−10.3 (12)	2	Strong	Strong
**32**	6.60	−9.8 (12)	−9.3 (6)	−8.9 (2)	1	Moderate	Weak

* CIc: Cross-interaction capability (Supplementary Information of [10]).

**Table 5:** Best coefficients of determination between molecular descriptors and experimental FAR values.

Compounds	EEig11x	X5	WTPT-2	GCUT_SLOGP_3	GCUT_SMR_3
**3–21**	0.84 ^1^	0.85 ^1^	0.78 ^2^	0.73	0.75
**22–32**	0.11	0.43	0.24	0.37	0.39

^1^ Compound **3** was found as an outlier for these descriptors; ^2^ Compound **17** was found as an outlier for this descriptor. EEig11x: Eigenvalue 11 from edge adj. Matrix weighted by edge degrees; X5: connectivity index of order 5; WTPT-2: weighted path descriptor described by Randic (molecular ID/number of atoms); GCUT_SLOGP_3: largest GCUT descriptor using atomic contribution to logP; GCUT_SMR_3: largest GCUT descriptor using atomic contribution to molar refractivity.

**Table 6:** Statistical data for all generated QSAR models.

	LR	MLPRegression	SMOReg
**R^2^**	0.937	0.994	0.933
**MAE**	6.941	2.609	6.453
**RMSE**	9.998	3.216	10.56
**q^2^**	0.887	0.932	0.894
**R^2^_pred_**	0.763	0.827	0.709

LR: linear regression; MLPRegression: multilayer perception regression; MAE: mean absolute error; RMSE: root mean squared error.

## Data Availability

The data presented in this study are available in article and supplementary material.

## Supplementary Materials

The following are available online at https://www.mdpi.com/1424-8247/14/9/862/s1, Figure S1: Flow cytometry data for parental (chemosensitive, PAR) mouse lymphoma cells, Figure S2: Flow cytometry data for multidrug resistant (MDR) mouse lymphoma cells, Figure S3: Flow cytometry data for verapamil (positive control) tested at 20 µM in multidrug resistant (MDR) mouse lymphoma cells, Figure S4: Flow cytometry data for 3-((2″-naphthalenylmethylene)hydrazineylidene) dregamine (compound **18**) tested at 0.2 µM in multidrug resistant (MDR) mouse lymphoma cells, Figure S5: Flow cytometry data for 3-((2″-naphthalenylmethylene)hydrazineylidene) dregamine (compound **18**) tested at 2 µM in multidrug resistant (MDR) mouse lymphoma cells, Figure S6: ^1^H NMR spectrum of compound **1** (300 MHz, CDCl_3_), Figure S7: ^13^C NMR spectrum of compound **1** (75 MHz, CDCl_3_), Figure S8: ^1^H NMR spectrum of compound **5** (300 MHz, MeOD + CDCl_3_), Figure S9: ^13^C NMR spectrum of compound **5** (75 MHz, MeOD + CDCl_3_), Figure S10: ^1^H NMR spectrum of compound **15** (300 MHz, MeOD), Figure S11: ^13^C NMR spectrum of compound **15** (75 MHz, MeOD), Figure S12: ^1^H NMR spectrum of compound **19** (300 MHz, CDCl_3_ + MeOD), Figure S13: ^13^C NMR spectrum of compound **19** (75 MHz, CDCl_3_ + MeOD), Figure S14: ^1^H NMR spectrum of compound **21** (300 MHz, MeOD), Figure S15: ^13^C NMR spectrum of compound **21** (75 MHz, MeOD), Figure S16: ^1^H NMR spectrum of compound **22** (300 MHz, CDCl_3_), Figure S17: ^13^C (APT) NMR spectrum of compound **22** (75 MHz, CDCl_3_), Figure S18: ^1^H NMR spectrum of compound **23** (300 MHz, CDCl_3_), Figure S19: ^13^C (APT) NMR spectrum of compound **23** (75 MHz, CDCl_3_), Figure S20: ^1^H NMR spectrum of compound **24** (300 MHz, CDCl_3_), Figure S21: ^13^C NMR spectrum of compound **24** (75 MHz, CDCl_3_), Figure S22: ^1^H NMR spectrum of compound **28** (75 MHz, CDCl_3_), Figure S23: ^1^H NMR spectrum of compound **28** (75 MHz, CDCl_3_ + D_2_O), Figure S24: COSY spectrum of compound **22** (300 MHz, CDCl_3_), Figure S25: COSY spectrum of compound **24** (300 MHz, CDCl_3_), Figure S26: HMBC spectrum of compound **25** (300 MHz, CDCl_3_), Figure S27: HMBC spectrum of compound **29** (300 MHz, CDCl_3_), Figure S28: Representative plots of molecular descriptors (A) EEig11x, (B) X5, (C) WTPT-2, (D) GCUT_SLOGP_3, and (E) GCUT_SMR_3 versus experimental FAR values for the azines **3**–**19**. No correlation was found for semicarbazones set (**22**–**32**), and thus was not represented; Table S1: Multidrug resistance inhibitory activities of parental compound (**1**), and derivatives **3–32** on mouse T-lymphoma cells (L5178Y cells), Table S2: Calculated TPSA, HBD and HBA values for compounds **1, 3**–**32**, Table S3: Effect of compounds **1**, **3**–**32** in combination with doxorubicin on human *ABCB1*-gene transfected mouse T-lymphoma cells (MDR cells), Table S4: Molecular descriptors used for the univariate linear regression, Table S5: Molecular descriptors used for the multivariate linear regression model.
