# Supplementary material for: Exploring the Monoterpene Indole Alkaloid Scaffold for Reversing P-Glycoprotein-Mediated Multidrug Resistance in Cancer
Source: Pharmaceuticals (Basel). 2021 Aug 28;14(9):862. doi: 10.3390/ph14090862 (PMC8493801; doi:10.3390/ph14090862)
Supplement: Supplementary file 1 [file pharmaceuticals-14-00862-s001.zip › pharmaceuticals-1340404 Supplementary Materials.pdf]

Supporting Information

Exploring the monoterpene indole alkaloid scaffold for reversing P-glycoprotein-mediated multidrug resistance in cancer

David S. P. Cardoso[1], Nikoletta Szemerédi[2], Gabriella Spengler[2], Silva Mulhovo[3], Daniel J. V. A. dos Santos[1,4], and Maria-José U. Ferreira[1*]

[1]Research Institute for Medicines (iMed.ULisboa), Faculty of Pharmacy, Universidade de Lisboa, Av. Prof. Gama Pinto, 1649-003 Lisbon, Portugal.

[2] Department of Medical Microbiology, Albert Szent-Györgyi Health Center and Faculty of Medicine, University of Szeged, Semmelweis utca 6, 6725 Szeged, Hungary.

[3]Centro de Estudos Moçambicanos e de Etnociências (CEMEC), Faculdade de Ciências Naturais e Matemática, Universidade Pedagógica Campus de Lhanguene, Av. de Moçambique, 21402161 Maputo, Moçambique.

[4]LAQV@REQUIMTE/Department of Chemistry and Biochemistry, Faculty of Sciences, University of Porto, Rua do Campo Alegre, 4169-007 Porto, Portugal.

Corresponding Author

* Phone: +351 217946475. Fax: +351 217946470. E-mail: mjuferreira@ff.ulisboa.pt.

Table of Contents:

I)	Rhodamine-123 Accumulation Assays	Page S3
II)	Flow Cytometry Data	Page S7
III)	TPSA and hydrogen-bond descriptors	Page S12
IV)	Combinational chemotherapy results	Page S13
V)	NMR spectra	Page S14
VI)	Computational studies	Page S48

I) Rhodamine-123 Accumulation Assays (Compounds 1, 3-32)

Table S1. Multidrug resistance inhibitory activities of parental compound (**1**), and derivatives **3-32** on mouse T-lymphoma cells (L5178Y cells).

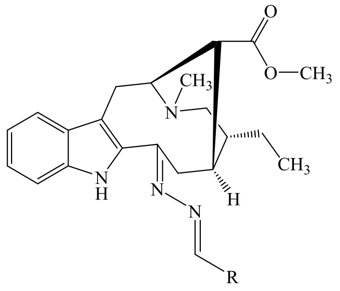

3-21

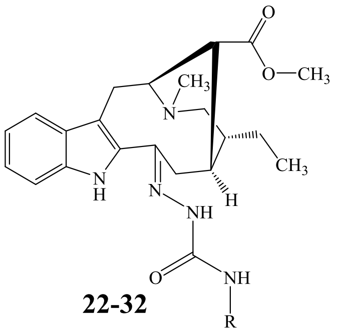

22-32

**Compound**	**R**	**Conc (μM)**	**FAR[a]**	**FSC[b]**	**SSC[c]**	**FL-1[d]**
PAR cells	**-**	-	-	1846	568	99.3
MDR cells	**-**	-	-	2083	1051	0.40
**1**	**Dregamine**	0.2	1.10	2368	1374	2.140
		2	1.03	2366	1390	2.010
**3**	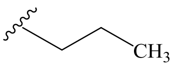	0.2	0.81	2394	1427	1.570
		2	1.22	2340	1389	2.380
**4**	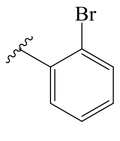	0.2	4.99	2325	1366	9.700
		2	33.9	2313	1367	[illegible]6.000
**5**	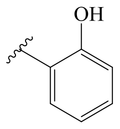	0.2	3.16	2151	797	13.1
		2	22.72	2096	800	94.3
**6**	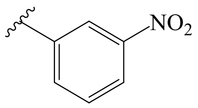	0.2	5.78	2155	798	24.0
		2	24.34	2102	799	101
**7**	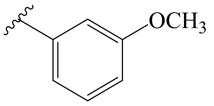	0.2	7.54	2134	798	31.3
		2	25.06	2078	847	104
**8**	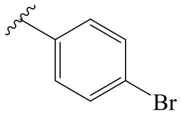	0.2	6.82	2155	807	28.3
		2	23.61	2115	844	98.0
**9**	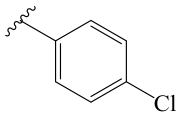	0.2	6.55	2216	847	27.2
		2	22.60	2153	803	93.8
**10**		0.2	1.70	2223	831	7.05

	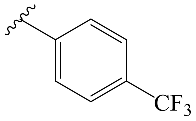	2	15.69	2208	844	65.1
**11**	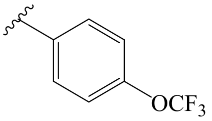	0.2	2.70	2223	852	11.2
		2	15.88	2166	840	65.9
**12**	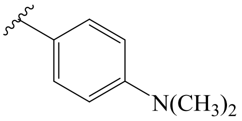	0.2	11.57	2198	847	48.0
		2	26.51	2196	830	110
**13**	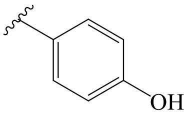	0.2	1.31	2190	823	5.42
		2	5.25	2195	864	21.8
**14**	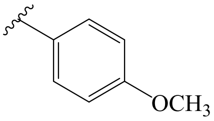	0.2	2.37	2186	822	9.83
		2	16.63	2131	835	69.0
**15**	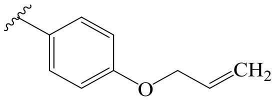	0.2	7.08	2196	831	29.4
		2	26.51	2084	875	110
**16**	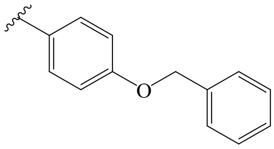	0.2	3.50	2184	1035	2.40
		2	104.59	2190	1035	71.8
**17**	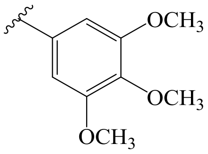	0.2	11.99	2203	1028	8.23
		2	100.07	2174	1021	68.7
**18**	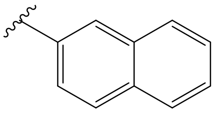	0.2	30.74	2215	1036	21.1
		2	126.58	2177	1059	86.9
**19**	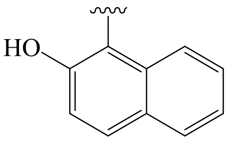	0.2	16.31	2197	1047	11.2
		2	128.48	2164	1056	88.2
**20**	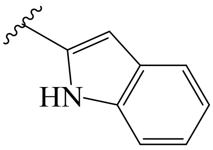	0.2	6.07	2200	1055	4.17
		2	106.19	2174	1067	72.9
**21**		0.2	3.00	2180	1058	2.06

	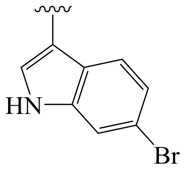	2	95.56	2179	1033	65.6
**22**	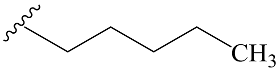	0.2	1.36	2163	1038	0.93
		2	2.93	2149	1044	2.01
**23**	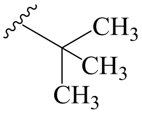	0.2	0.96	2158	1018	0.66
		2	1.10	2108	1070	0.76
**24**	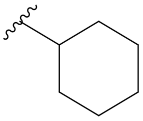	0.2	1.06	2176	1040	0.73
		2	1.82	2170	1044	1.25
**25**	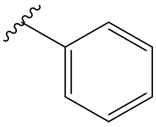	0.2	1.26	2162	1057	0.87
		2	5.93	2134	1045	4.07
**26**	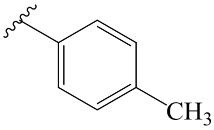	0.2	1.00	2331	1365	8.01
		2	8.77	2251	1401	70.5
**27**	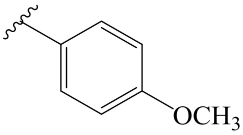	0.2	0.81	2235	1407	6.51
		2	3.20	2291	1440	25.7
**28**	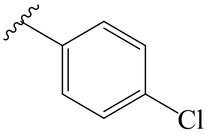	0.2	0.79	2266	1441	6.39
		2	7.80	2256	1463	62.7
**29**		0.2	1.16	2254	1459	9.34
	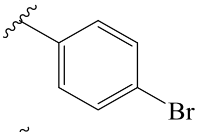	2	7.44	2245	1474	59.8
**30**	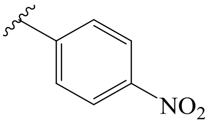	0.2	1.06	2255	1436	8.53
		2	7.29	2223	1478	58.6
**31**	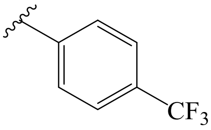	0.2	0.73	2243	1467	5.88
		2	4.80	2231	1503	38.6

**32**	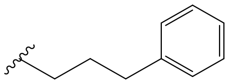	0.2	1.41	2227	1414	11.3
		2	6.60	2254	1454	53.1

[a]FAR (fluorescence activity ratio) values were determined by using the equation shown in the section 4.4.3. Verapamil 20 µM (positive control) FAR = 6.34; DMSO 2% (negative control) FAR = 0.79; [b]FSC: Forward scatter count of cells in the sample; [c]SSC: Side scatter count of cells in the sample; [d]FL-1: Mean fluorescence intensity of the cells.

II) Flow cytometry data

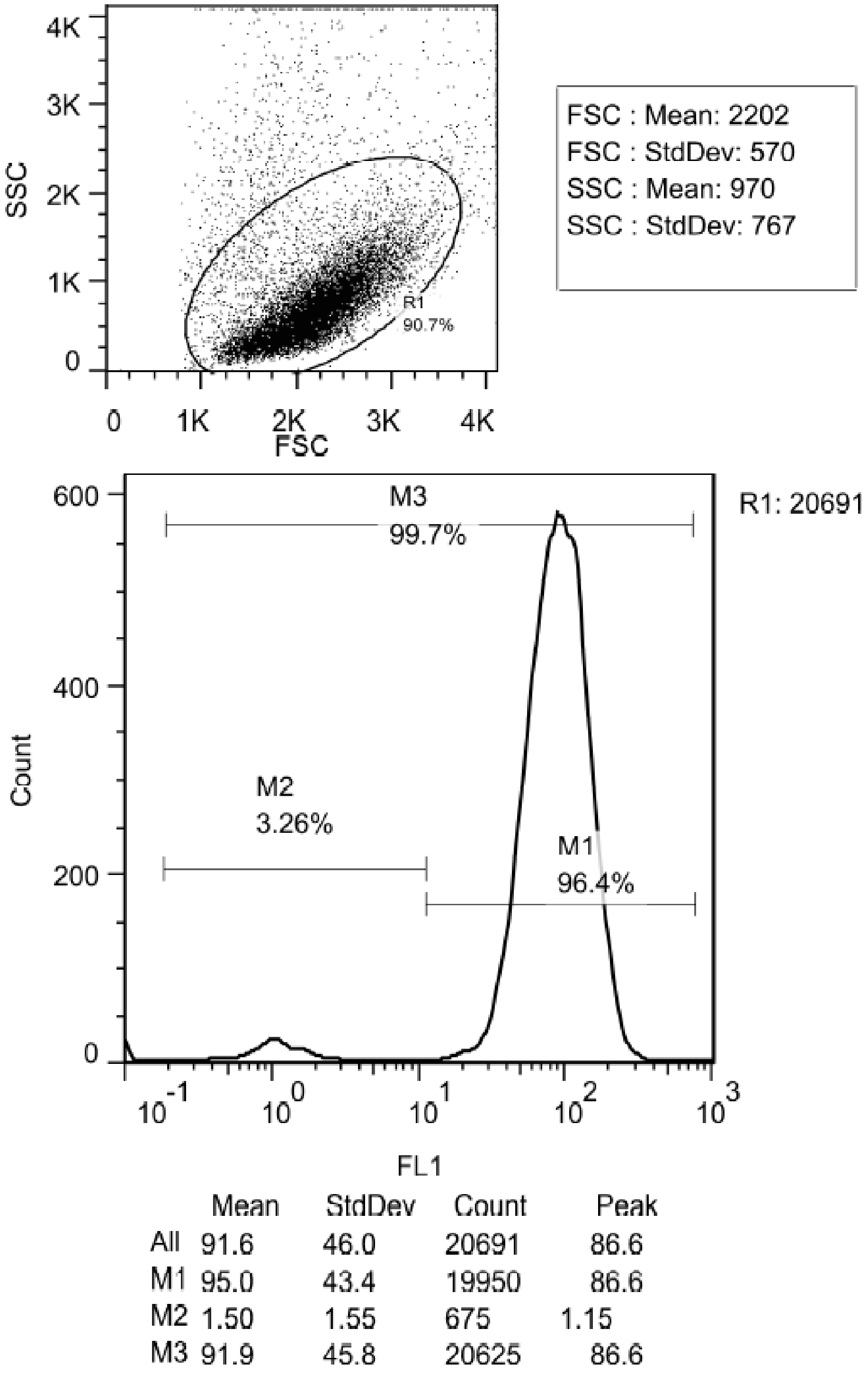

Figure S1. Flow cytometry data for parental (chemosensitive, PAR) mouse lymphoma cells.

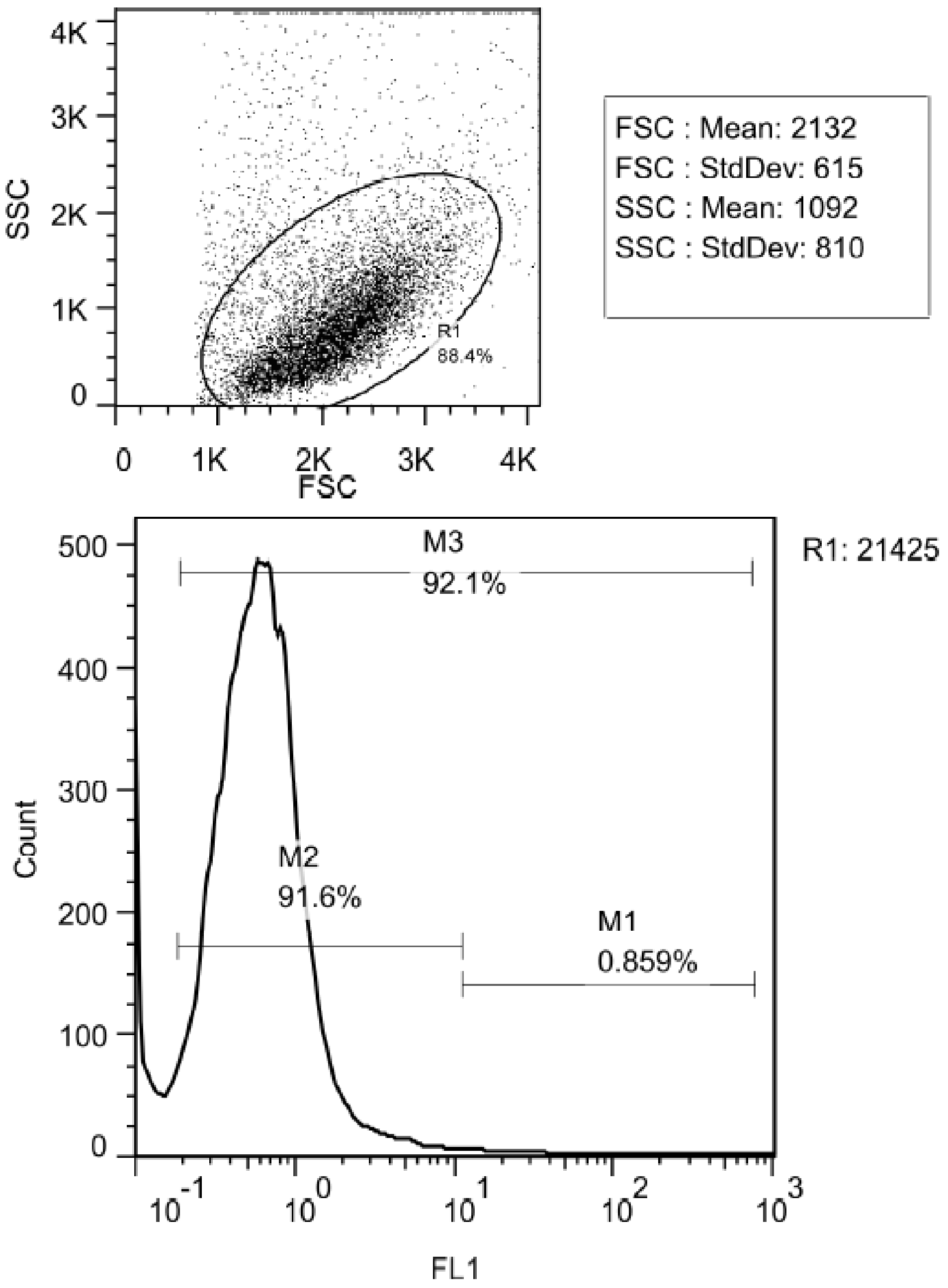

	Mean	StdDev	Count	Peak
All	1.04	4.71	21425	23.7
M1	36.9	34.9	184	23.7
M2	0.782	0.833	19627	0.698
M3	1.12	4.90	19732	0.698

Figure S2. Flow cytometry data for multidrug resistant (MDR) mouse lymphoma cells.

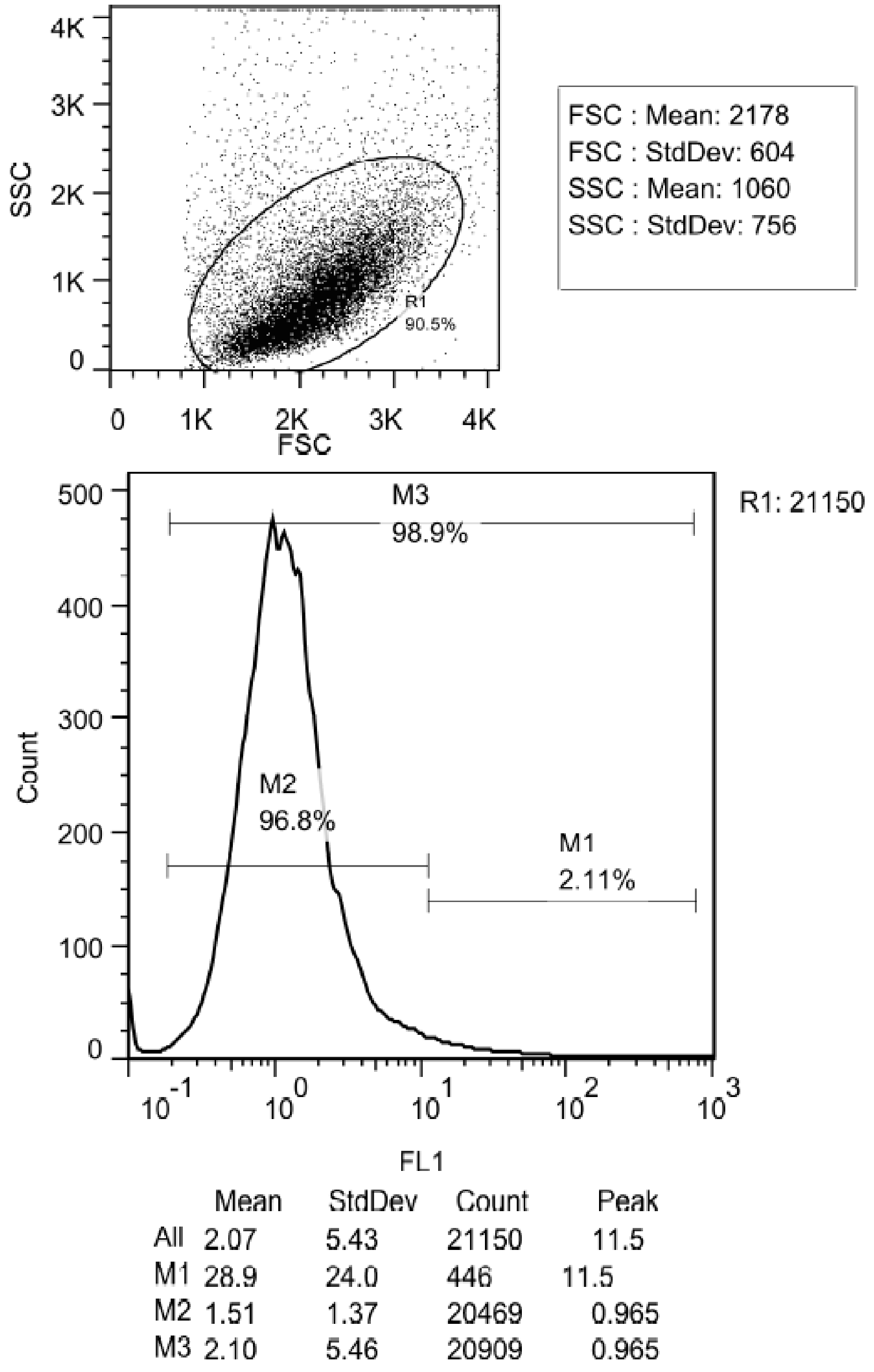

Figure S3. Flow cytometry data for verapamil (positive control) tested at 20 µM in multidrug resistant (MDR) mouse lymphoma cells.

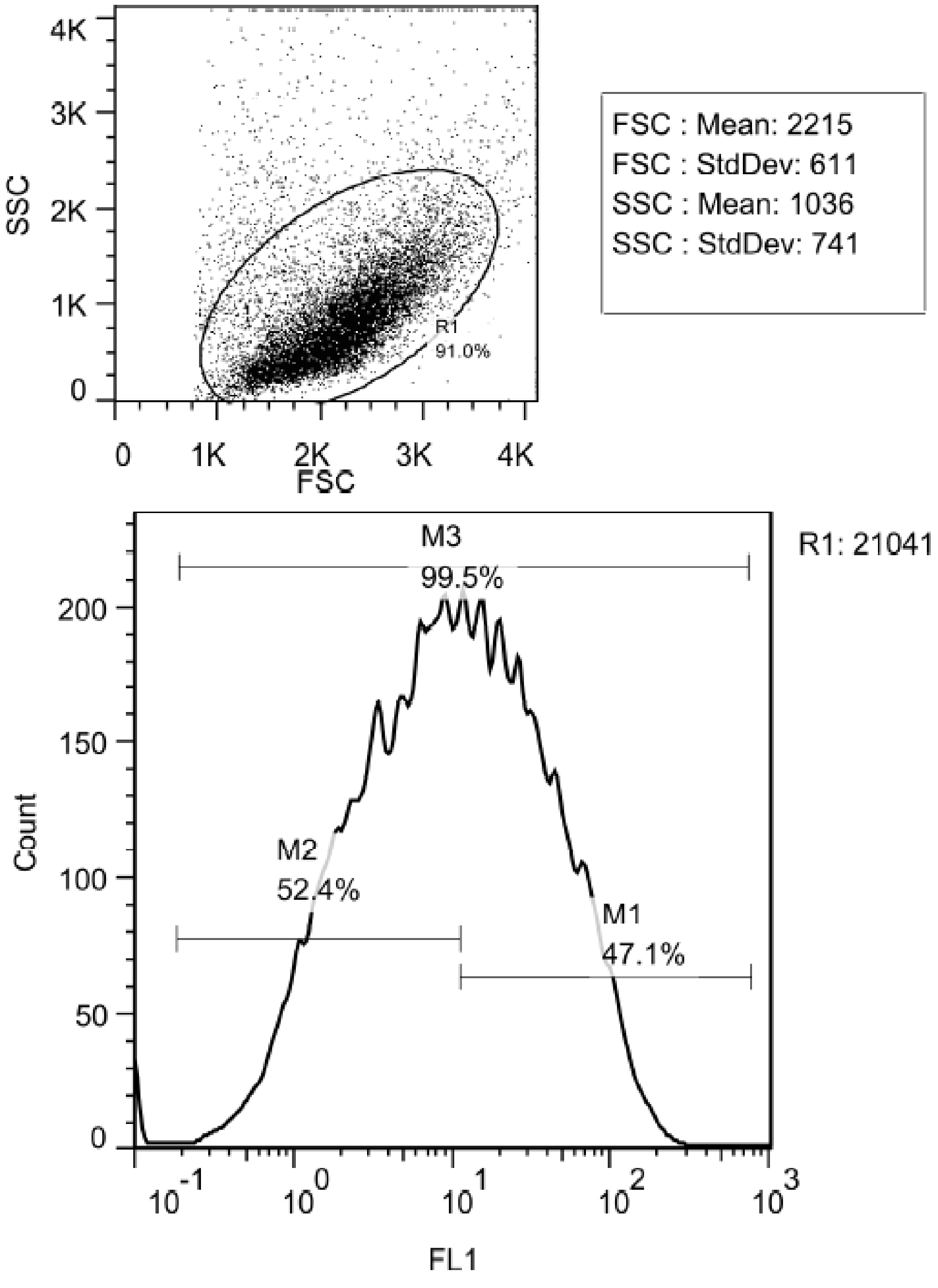

	Mean	StdDev	Count	Peak
All	21.1	28.8	21041	11.5
M1	39.8	33.2	9908	11.5
M2	4.56	2.98	11033	6.26
M3	21.2	28.9	20940	11.5

Figure S4. Flow cytometry data for *3-[(2''-naphthalenylmethylene)hydrazineylidene] dregamine* (compound **18**) tested at 0.2 µM in multidrug resistant (MDR) mouse lymphoma cells.

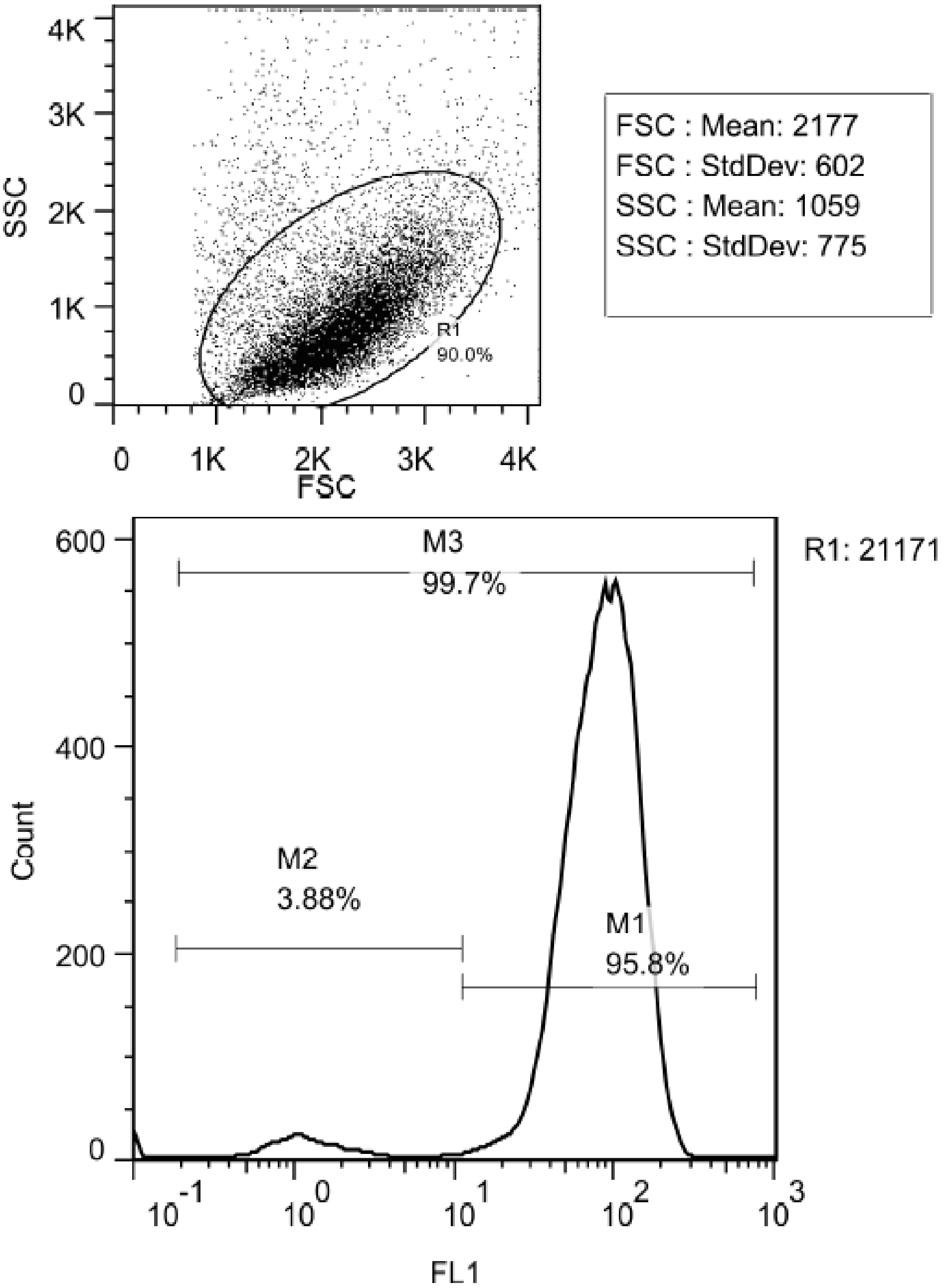

	Mean	StdDev	Count	Peak
All	86.9	45.2	21171	86.6
M1	90.7	42.4	20276	86.6
M2	1.76	1.83	822	1.15
M3	87.2	45.0	21098	86.6

Figure S5. Flow cytometry data for *3-[(2’’-naphthalenylmethylene)hydrazineylidene] dregamine* (compound **18**) tested at 2 μM in multidrug resistant (MDR) mouse lymphoma cells.

III) TPSA and hydrogen-bond descriptors

Table S2. Calculated TPSA, HBD and HBA values for compounds **1, 3-32**.

**Compound**	TPSA (Å^2)	HBD	HBA
**1**	62.4	1	3
**3**	70.1	1	4
**4**	70.1	1	4
**5**	90.3	2	5
**6**	115.9	1	4
**7**	79.3	1	5
**8**	70.1	1	4
**9**	70.1	1	4
**10**	70.1	1	4
**11**	79.3	1	5
**12**	73.3	1	4
**13**	90.3	2	5
**14**	79.3	1	5
**15**	79.3	1	5
**16**	79.3	1	5
**17**	97.7	1	7
**18**	70.1	1	4
**19**	90.3	2	5
**20**	85.8	2	4
**21**	85.8	2	4
**22**	98.8	3	4
**23**	98.8	3	4
**24**	98.8	3	4
**25**	98.8	3	4
**26**	98.8	3	4
**27**	108.1	3	5
**28**	98.8	3	4
**29**	98.8	3	4
**30**	144.6	3	4
**31**	98.8	3	4
**32**	98.8	3	4

TPSA (topological polar surface area), HBD (hydrogen-bond donor) and HBA (hydrogen-bond acceptor) values were obtained using MOE program software.

IV) Combinational chemotherapy results

Table S3. Effect of compounds **1**, **3-32** in combination with doxorubicin on human *ABCB1*-gene transfected mouse T-lymphoma cells (MDR cells)

Compounds	Best ratio*	CI at ED_{50}**	Interaction	SD +/-
1	1:8.0	0.756	Moderate synergism	0.053
3	1:87.7	0.305	Synergism	0.046
4	1:30.2	0.079	Very Strong Synergism	0.012
5	1:30.2	0.166	Strong Synergism	0.013
6	1:12.1	0.279	Strong Synergism	0.044
7	1:5.15	0.152	Strong Synergism	0.045
8	1:10.4	0.451	Synergism	0.057
9	1:36.6	0.194	Strong Synergism	0.021
10	1:32.5	0.272	Strong Synergism	0.023
11	1:46.8	0.601	Synergism	0.103
12	1:2.58	0.231	Strong Synergism	0.024
13	1:17.3	0.361	Synergism	0.027
14	1:48.1	0.402	Synergism	0.046
15	1:17.8	0.177	Strong Synergism	0.041
16	1:13.1	0.34	Synergism	0.036
17	1:44.0	0.309	Synergism	0.019
18	1:36.6	0.285	Strong Synergism	0.066
19	1:71.9	0.222	Strong Synergism	0.041
20	1:12.2	0.291	Strong Synergism	0.026
21	1:35.9	0.445	Synergism	0.051
22	1:9.54	0.219	Strong Synergism	0.024
23	1:13.7	0.163	Strong Synergism	0.021
24	1:25.4	0.301	Synergism	0.035
25	1:7.50	0.345	Synergism	0.084
26	1:5.72	0.36	Synergism	0.102
27	1:4.43	0.225	Strong Synergism	0.018
28	1:29.0	0.158	Strong Synergism	0.02
29	1:20.6	0.173	Strong Synergism	0.022
30	1:5.15	0.423	Synergism	0.035
31	1:18.0	0.296	Strong Synergism	0.023
32	1:4.96	0.384	Synergism	0.035

V) NMR Spectra

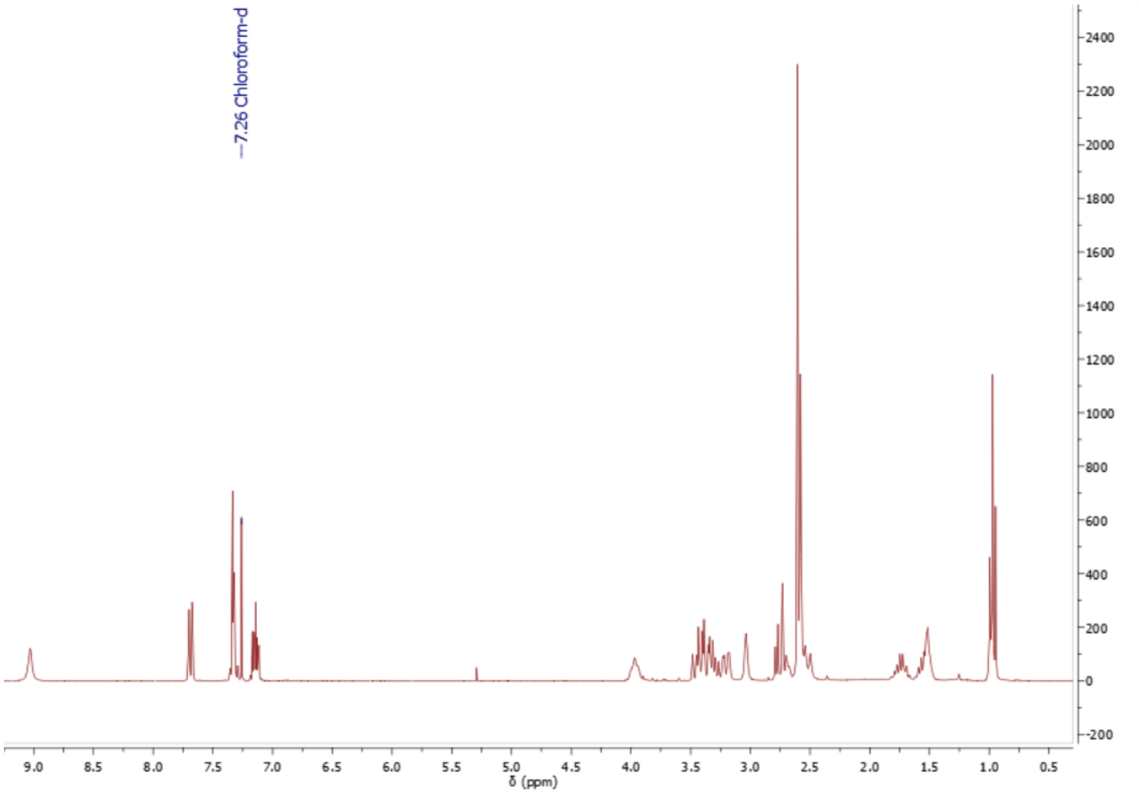

Figure S6. ^{1}H NMR spectrum of compound **1** (300 MHz, $CDCl_3$).

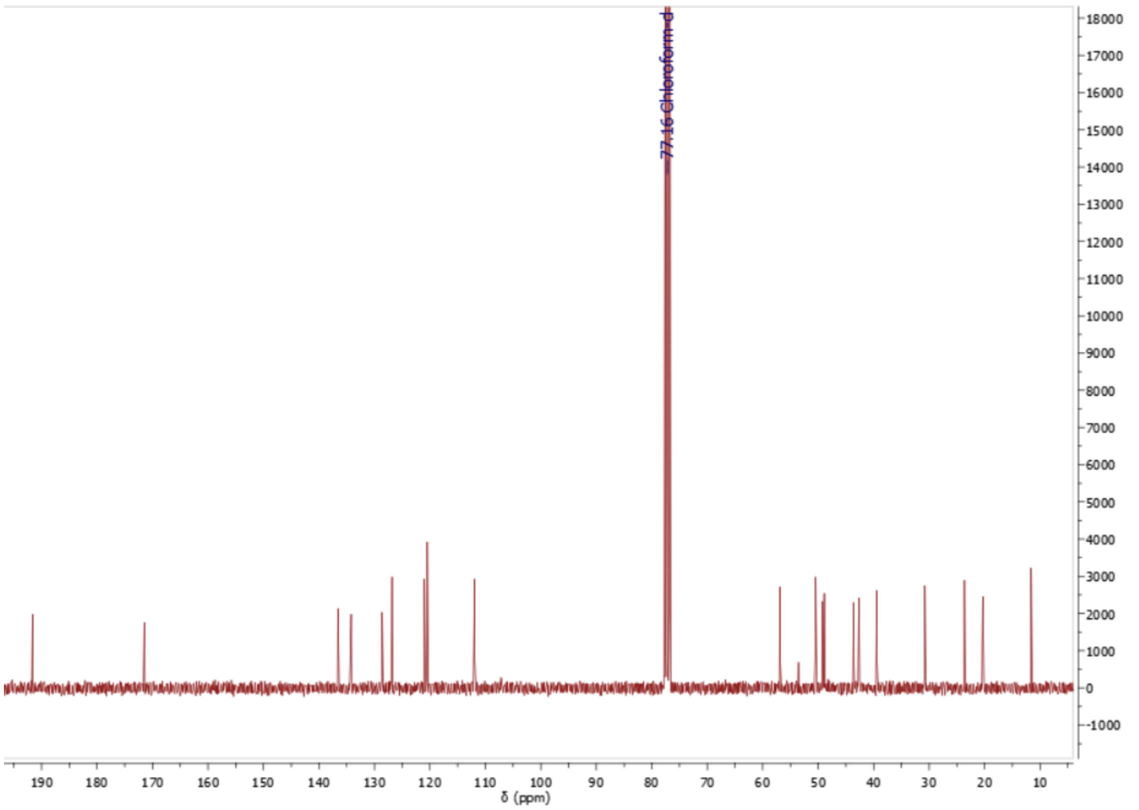

Figure S7. ^{13}C NMR spectrum of compound **1** (75 MHz, $CDCl_3$).

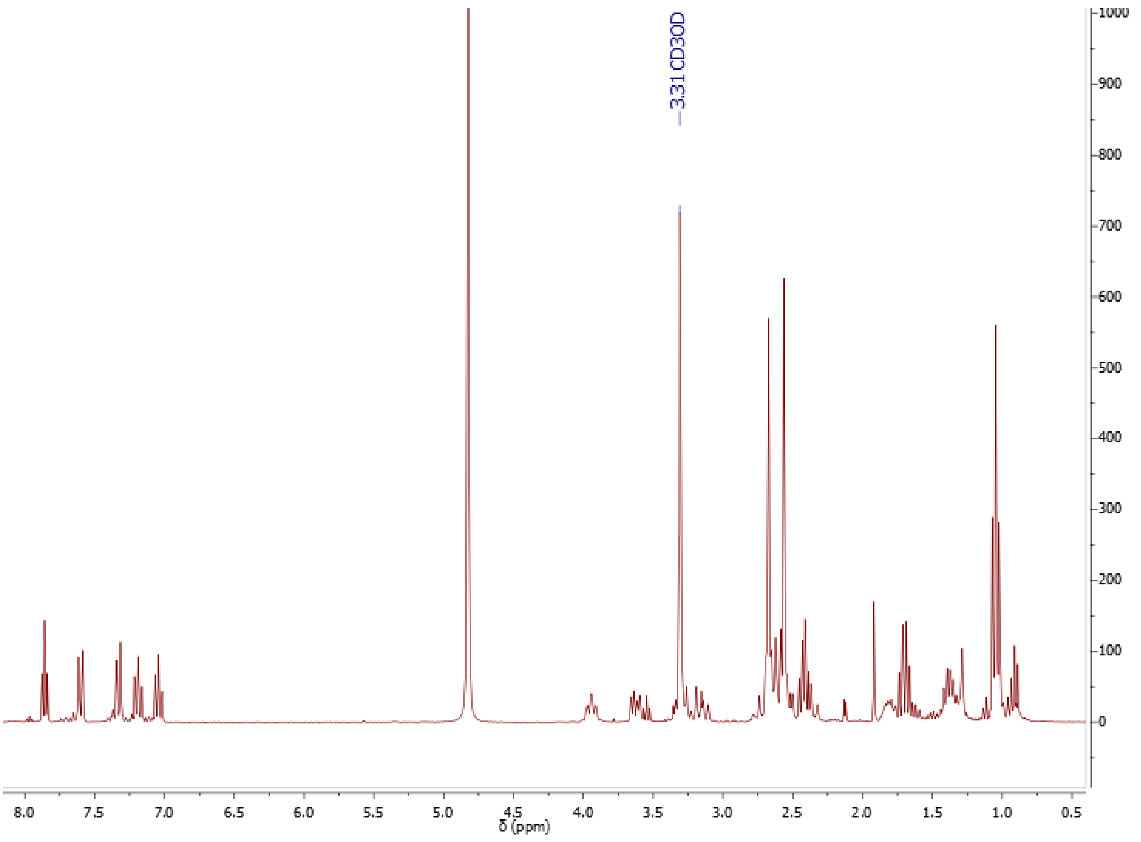

Figure S8. ^{1}H NMR spectrum of compound **3** (300 MHz, MeOD).

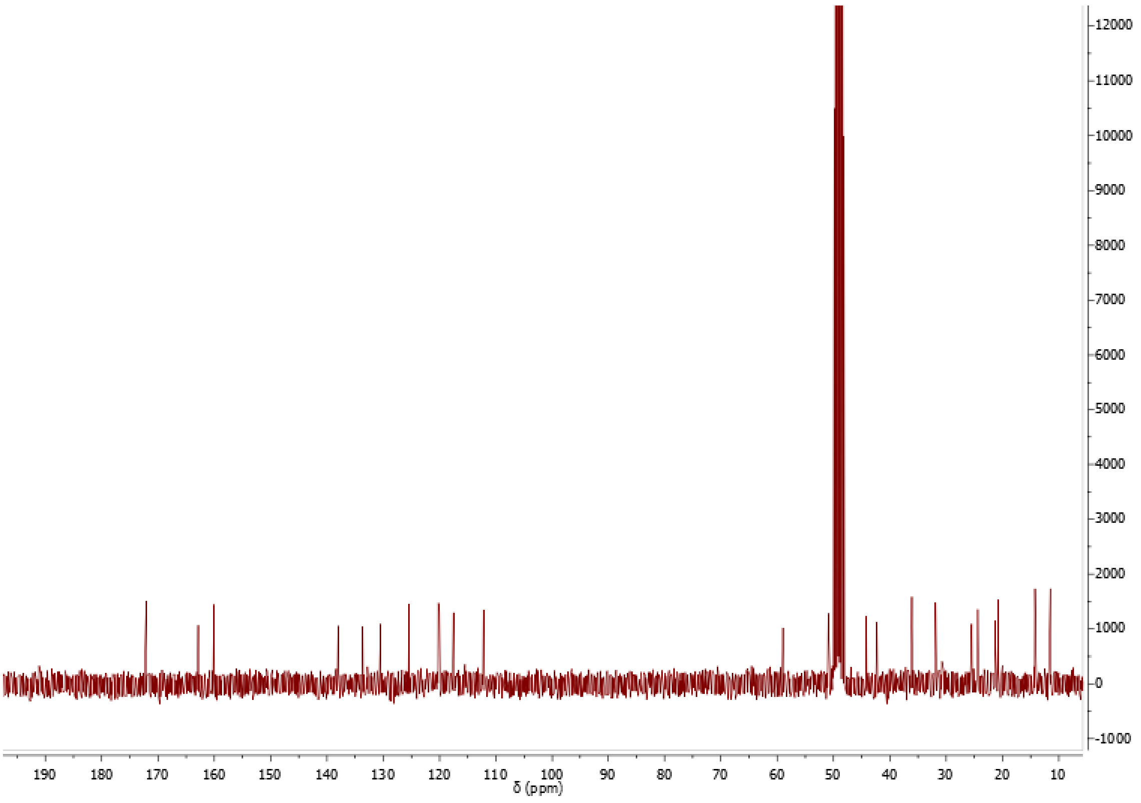

Figure S9. ^{13}C NMR spectrum of compound **3** (75 MHz, MeOD).

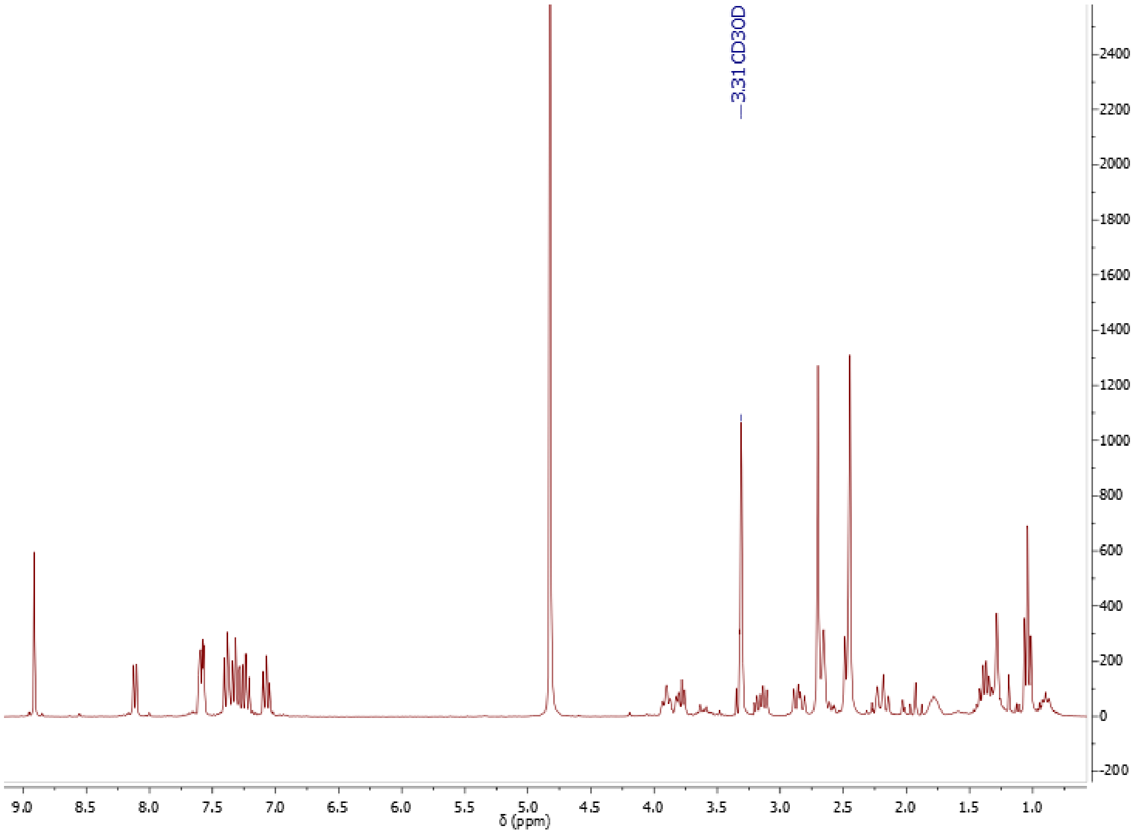

Figure S10. ^{1}H NMR spectrum of compound **4** (300 MHz, MeOD).

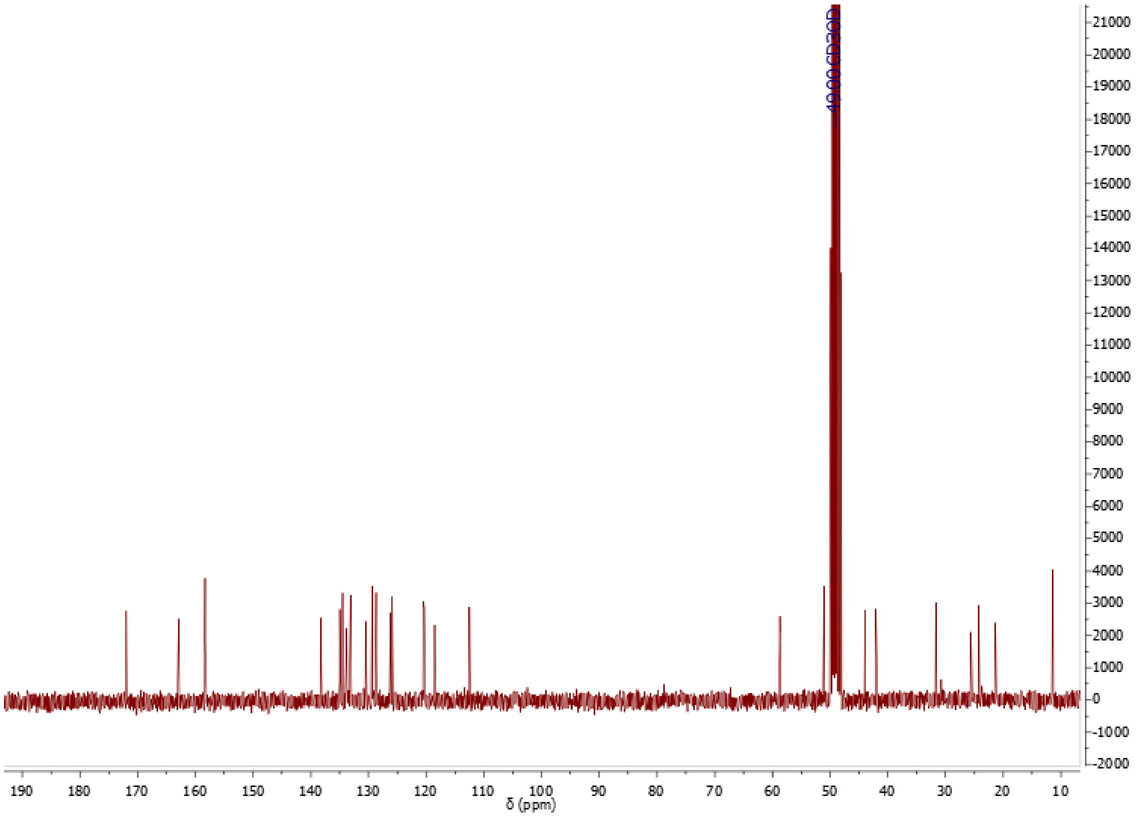

Figure S11. ^{13}C NMR spectrum of compound **4** (75 MHz, MeOD).

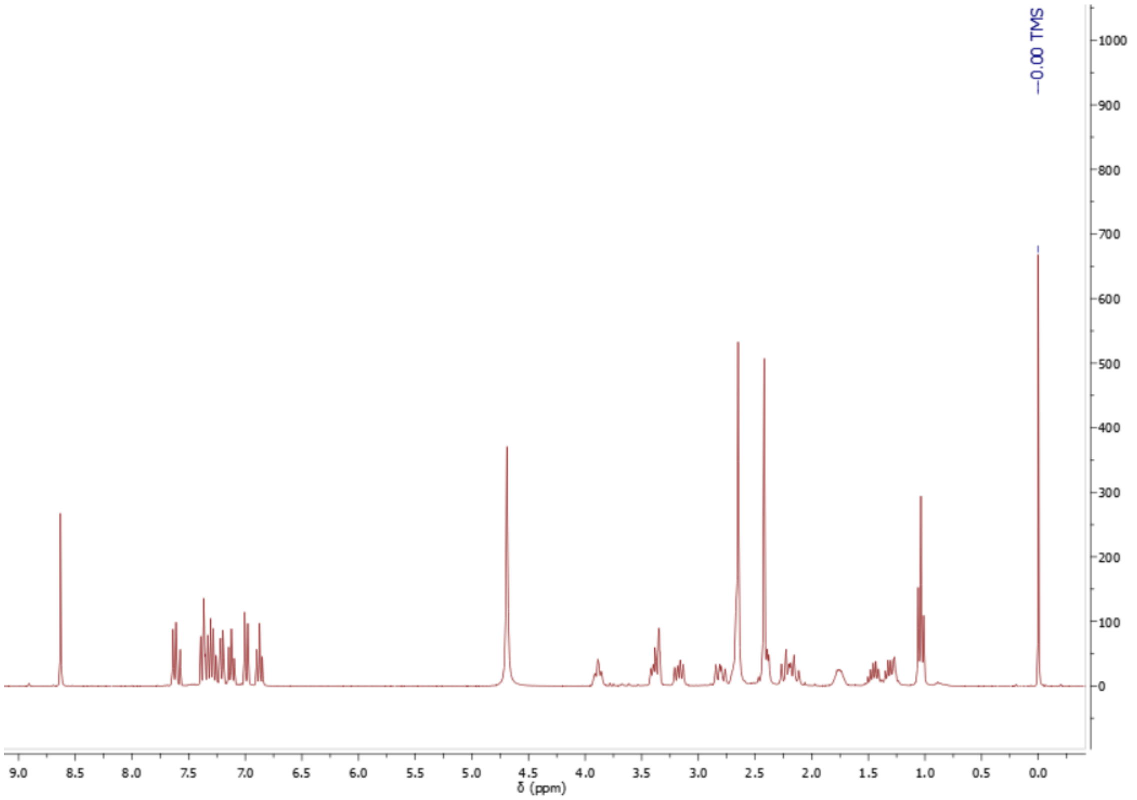

Figure S12. ^{1}H NMR spectrum of compound **5** (300 MHz, MeOD + $CDCl_3$).

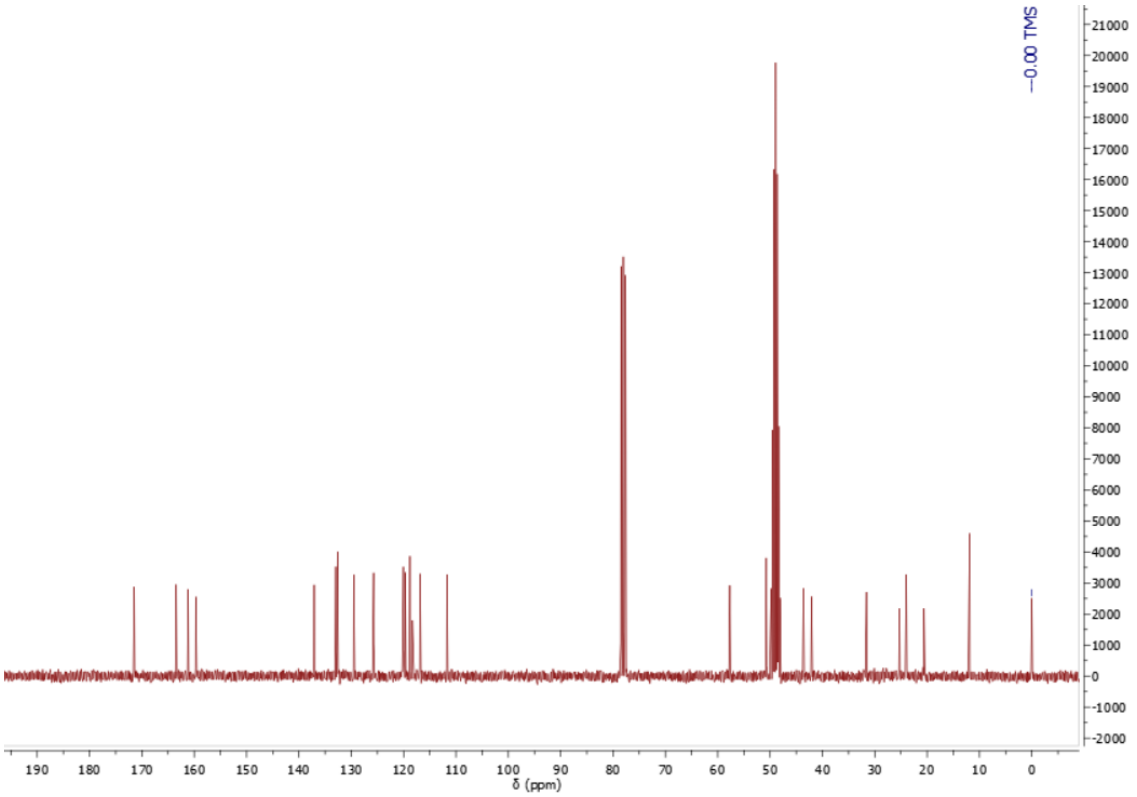

Figure S13. ^{13}C NMR spectrum of compound **5** (75 MHz, MeOD + $CDCl_3$).

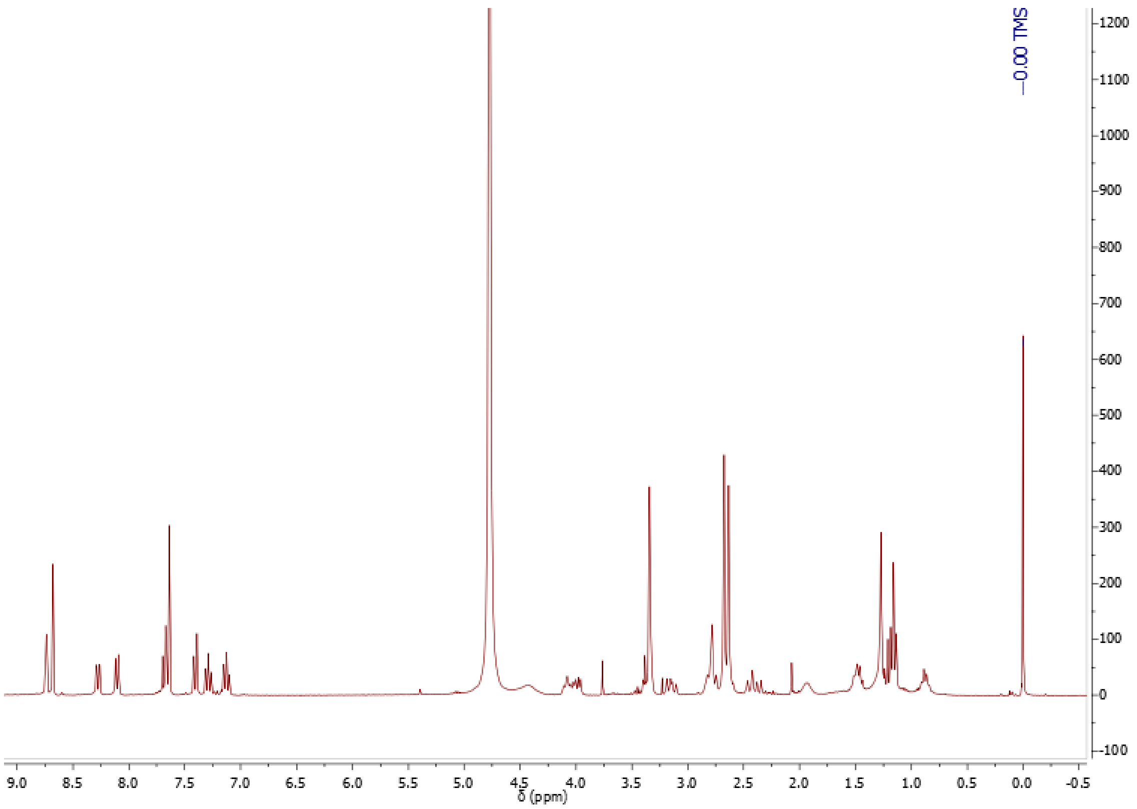

Figure S14. ^{1}H NMR spectrum of compound **6** (300 MHz, MeOD + $CDCl_3$).

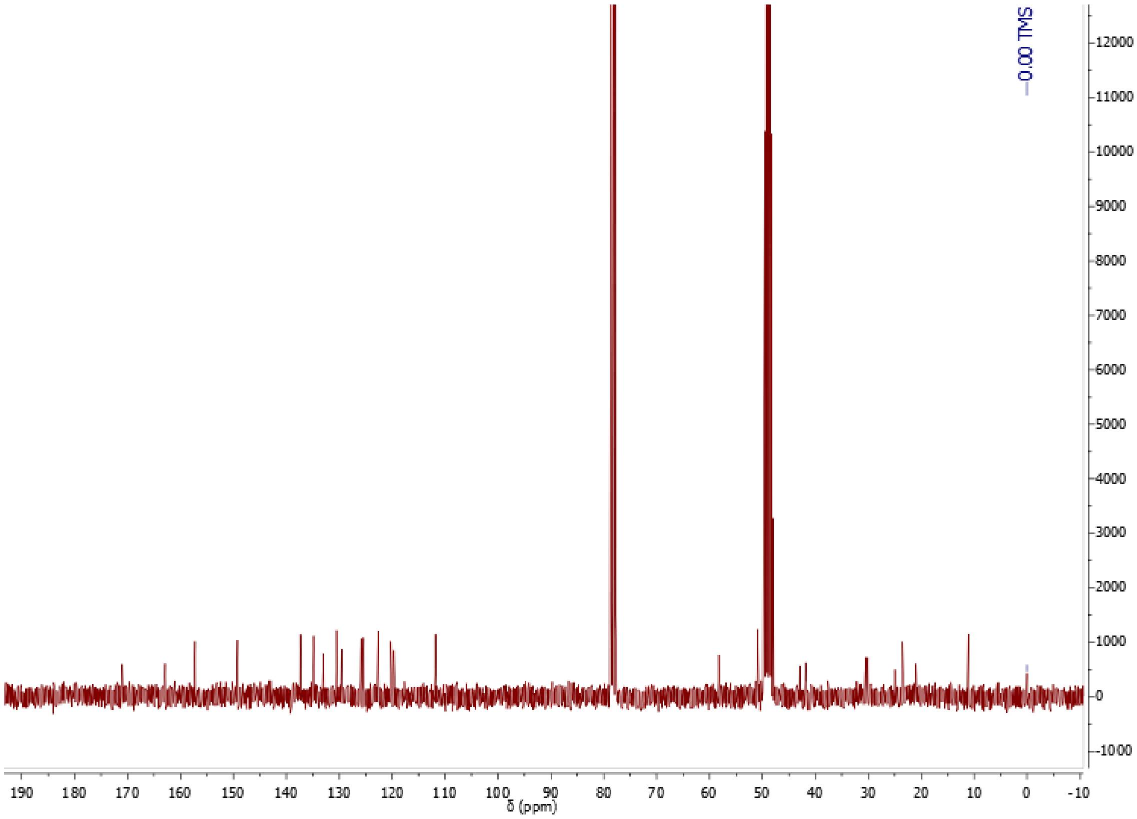

Figure S15. ^{13}C NMR spectrum of compound **6** (75 MHz, MeOD + $CDCl_3$).

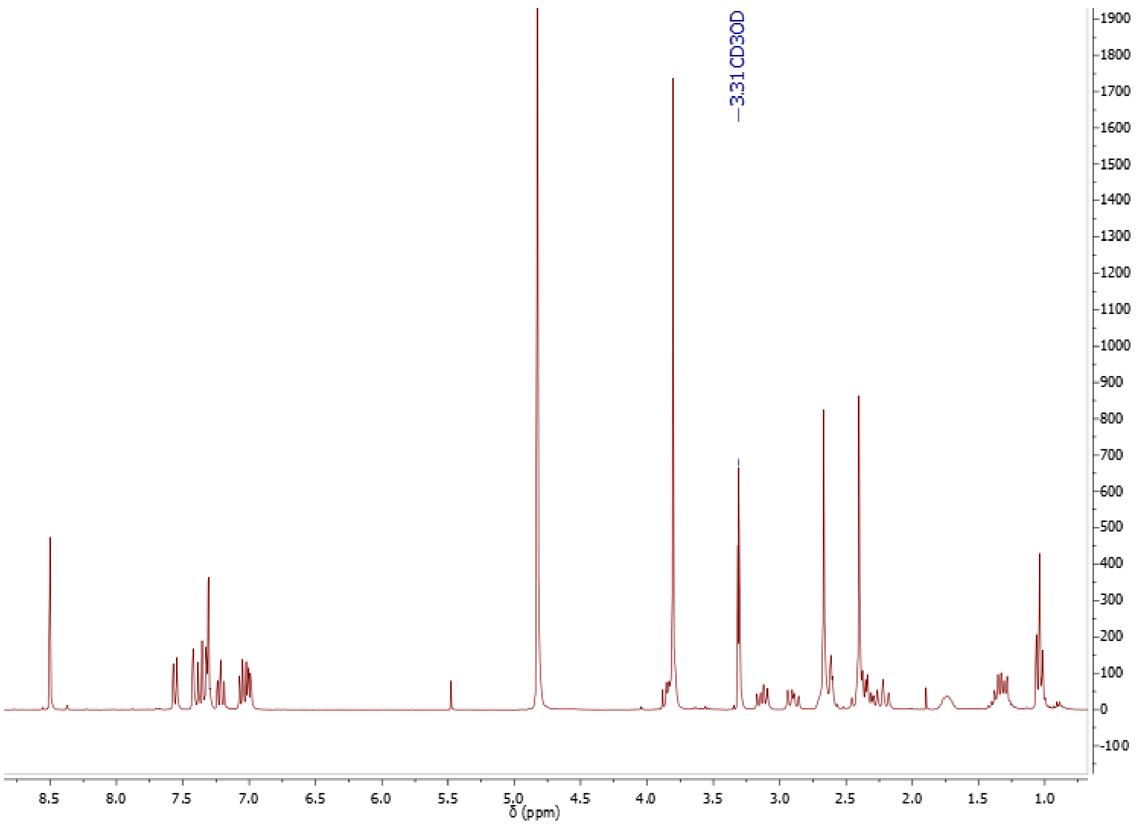

Figure S16. ^{1}H NMR spectrum of compound **7** (300 MHz, MeOD).

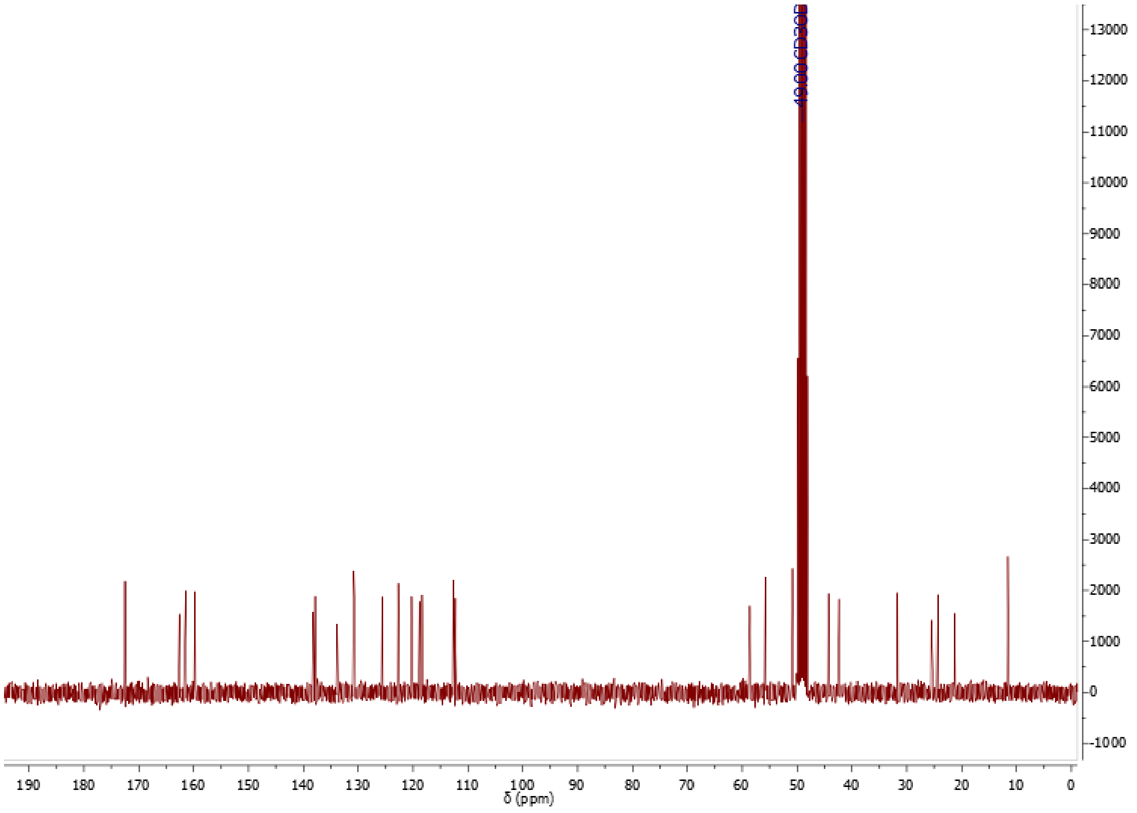

Figure S17. ^{13}C NMR spectrum of compound **7** (75 MHz, MeOD).

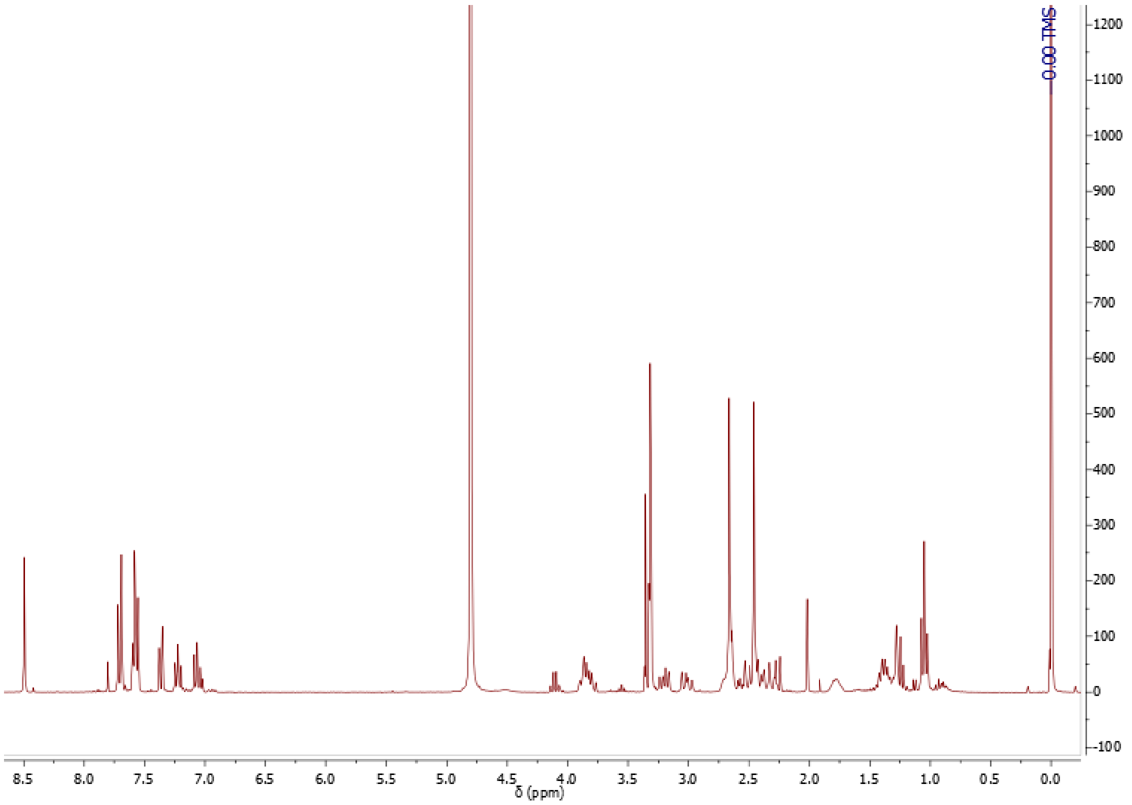

Figure S18. ^{1}H NMR spectrum of compound **8** (300 MHz, MeOD + $CDCl_3$).

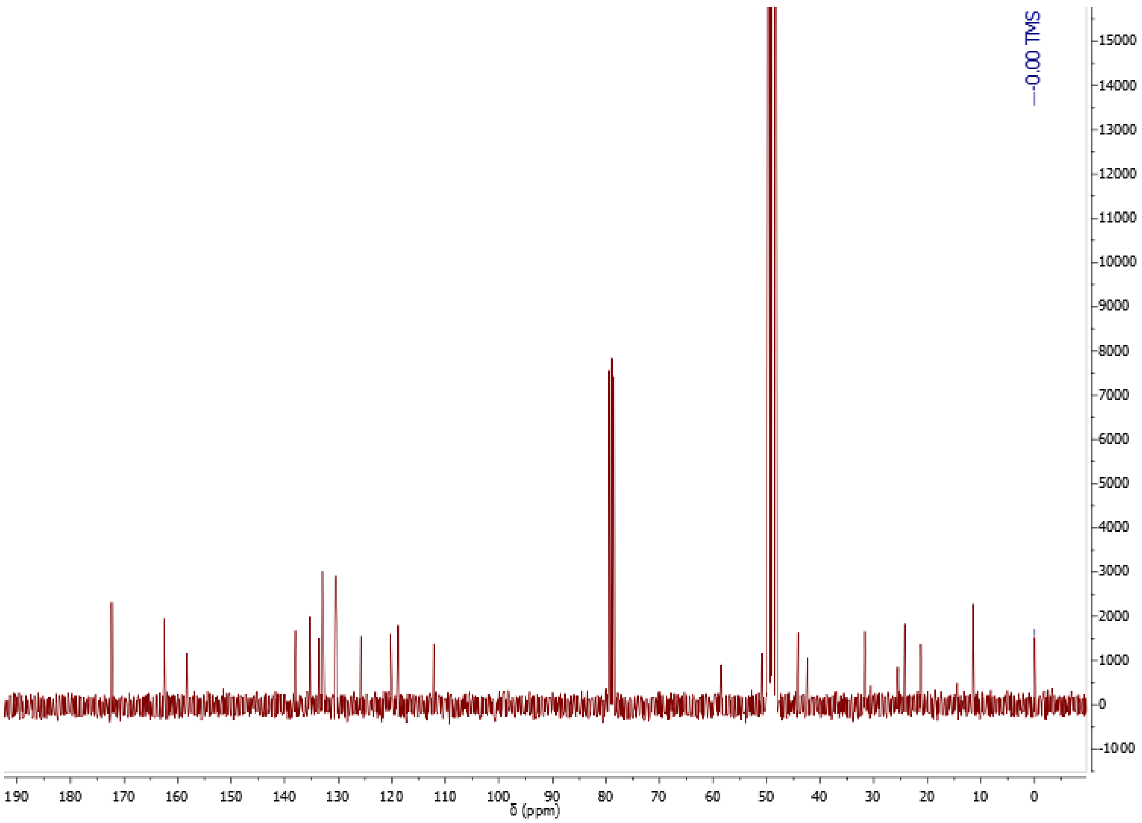

Figure S19. ^{13}C NMR spectrum of compound **8** (75 MHz, MeOD + $CDCl_3$).

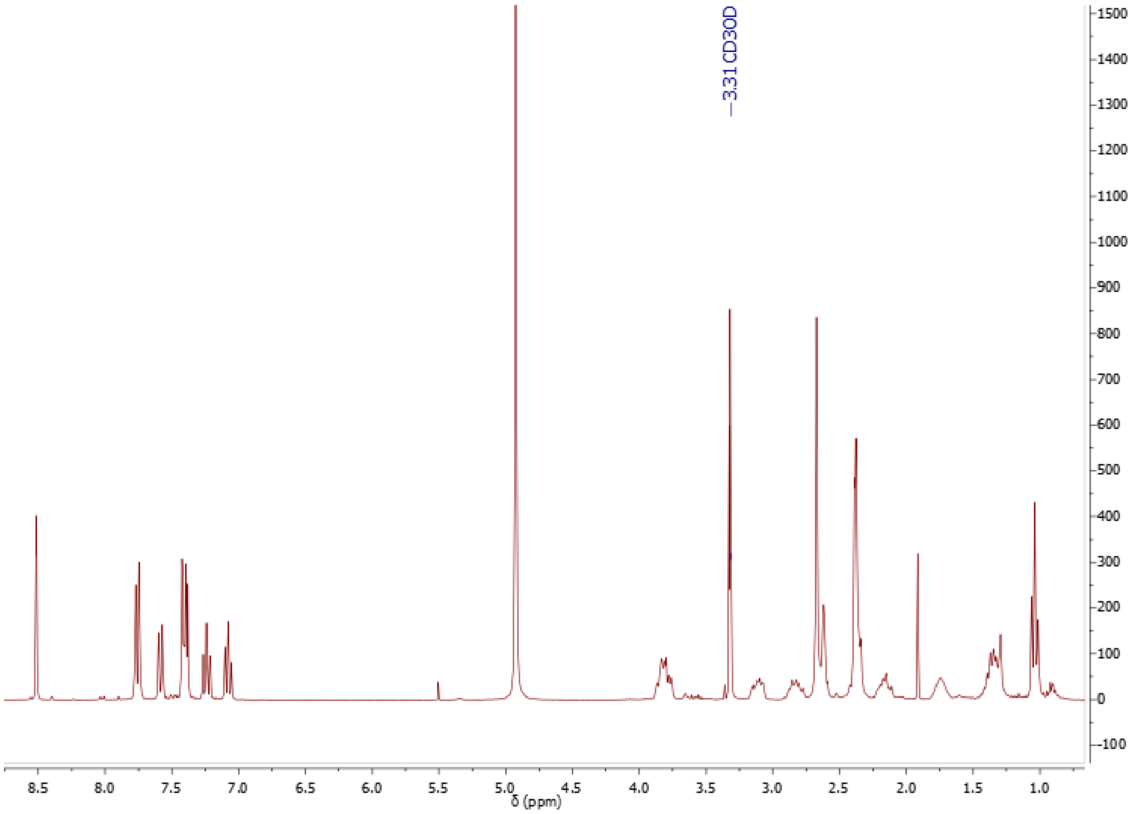

Figure S20. ^{1}H NMR spectrum of compound **9** (300 MHz, MeOD).

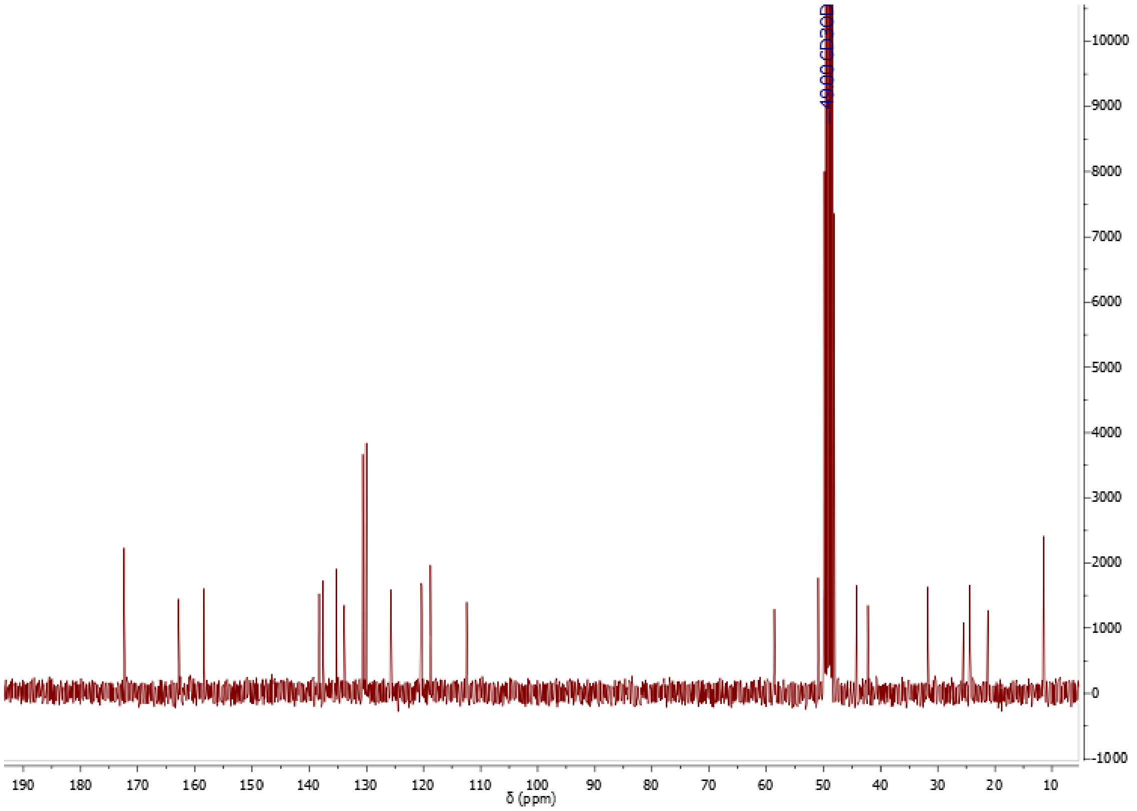

Figure S21. ^{13}C NMR spectrum of compound **9** (75 MHz, MeOD).

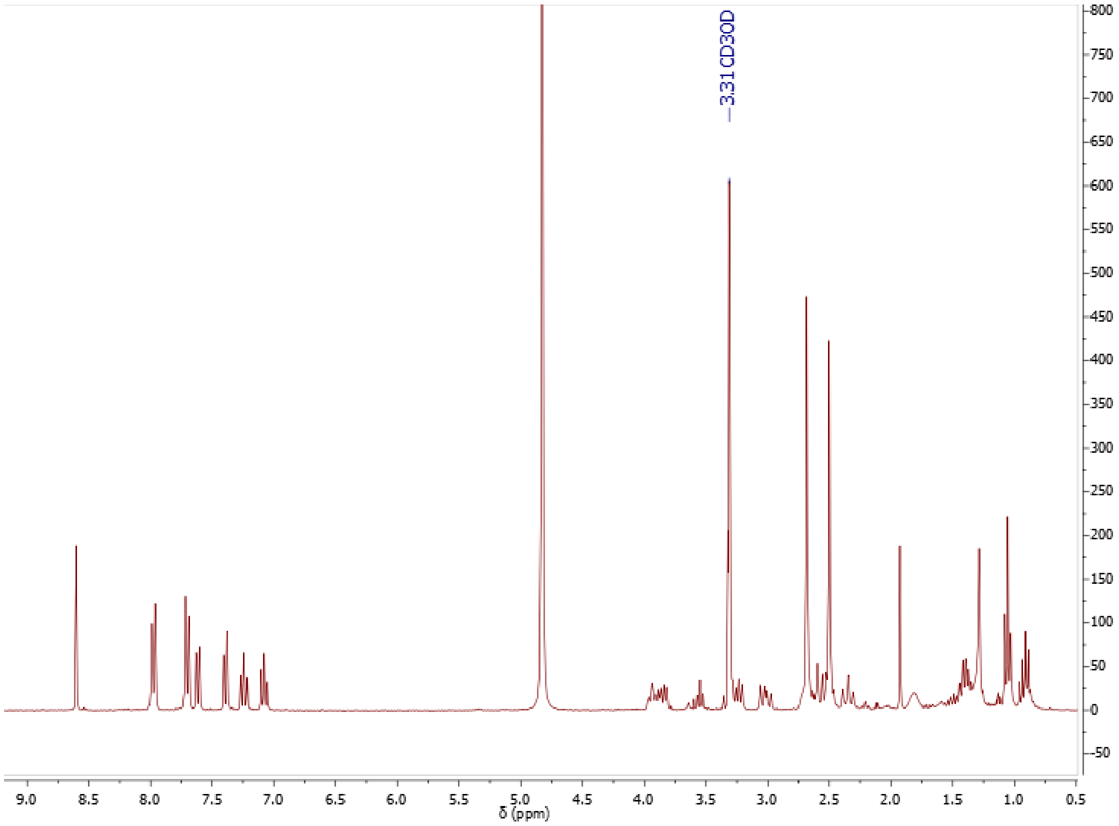

Figure S22. ^{1}H NMR spectrum of compound **10** (300 MHz, MeOD).

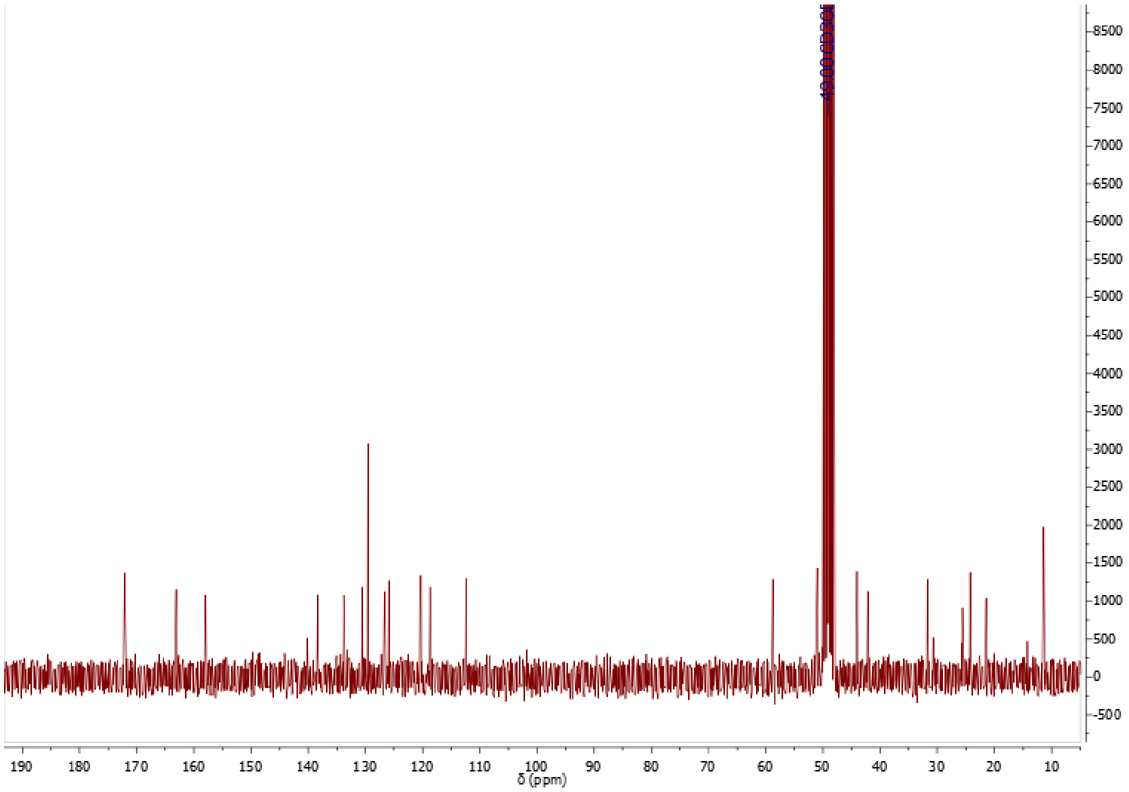

Figure S23. ^{13}C NMR spectrum of compound **10** (75 MHz, MeOD).

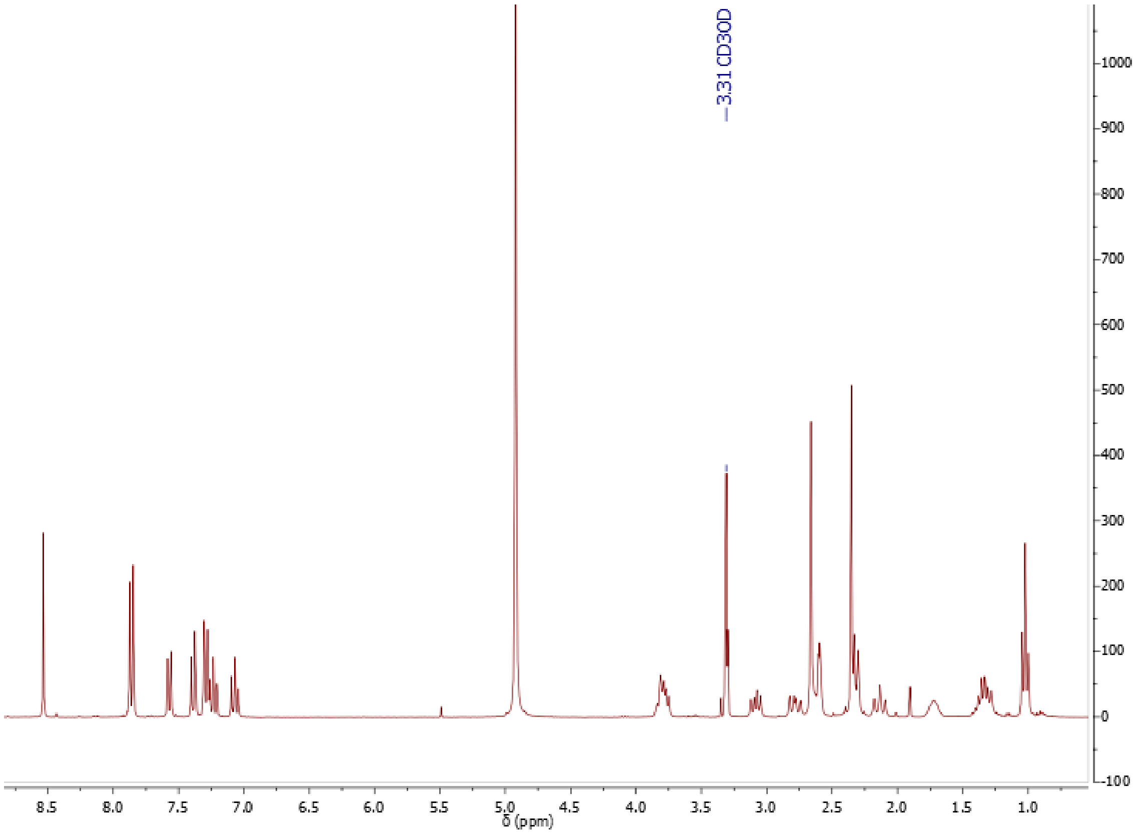

Figure S24. ^{1}H NMR spectrum of compound **11** (300 MHz, MeOD).

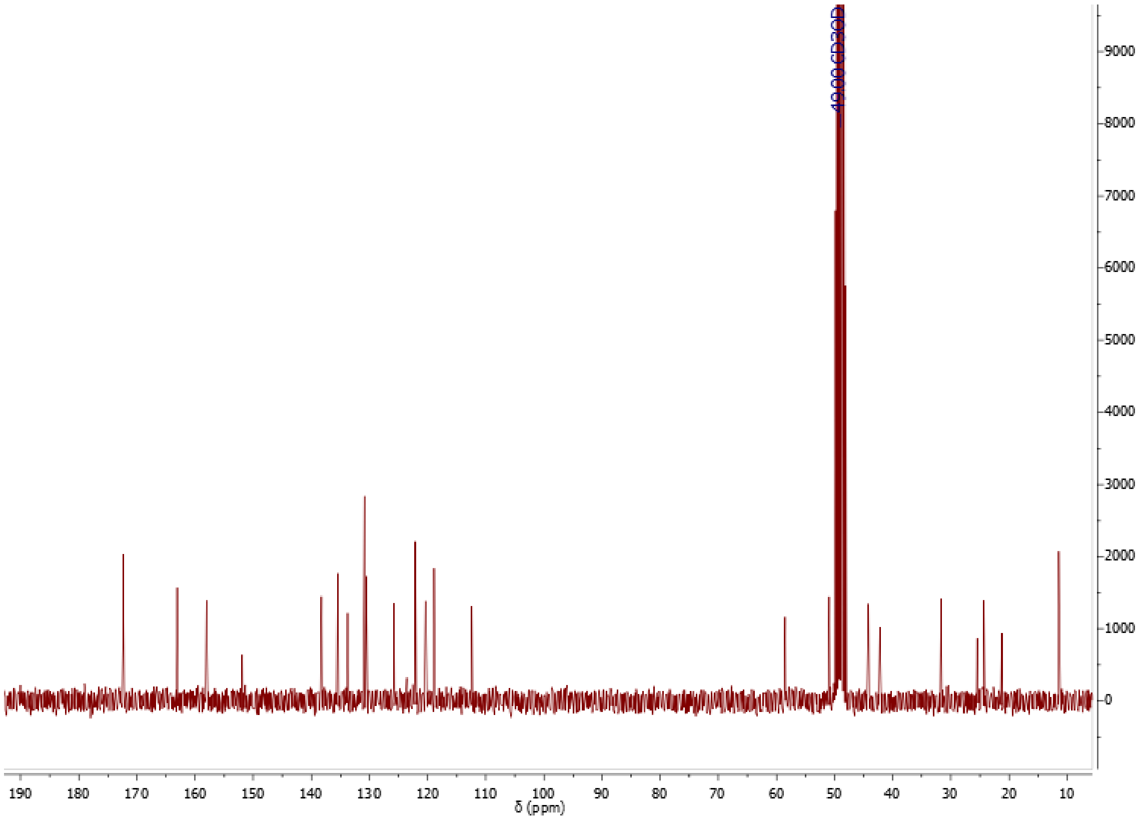

Figure S25. ^{13}C NMR spectrum of compound **11** (75 MHz, MeOD).

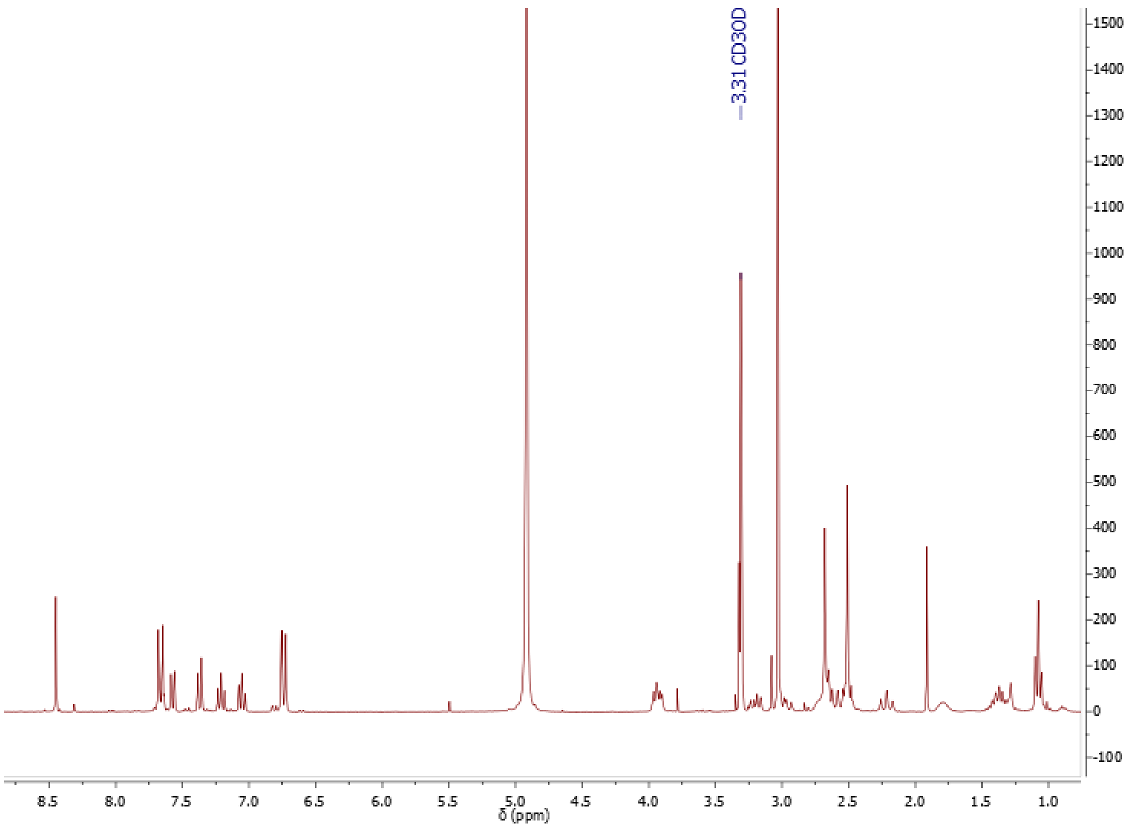

Figure S26. ^{1}H NMR spectrum of compound **12** (300 MHz, MeOD).

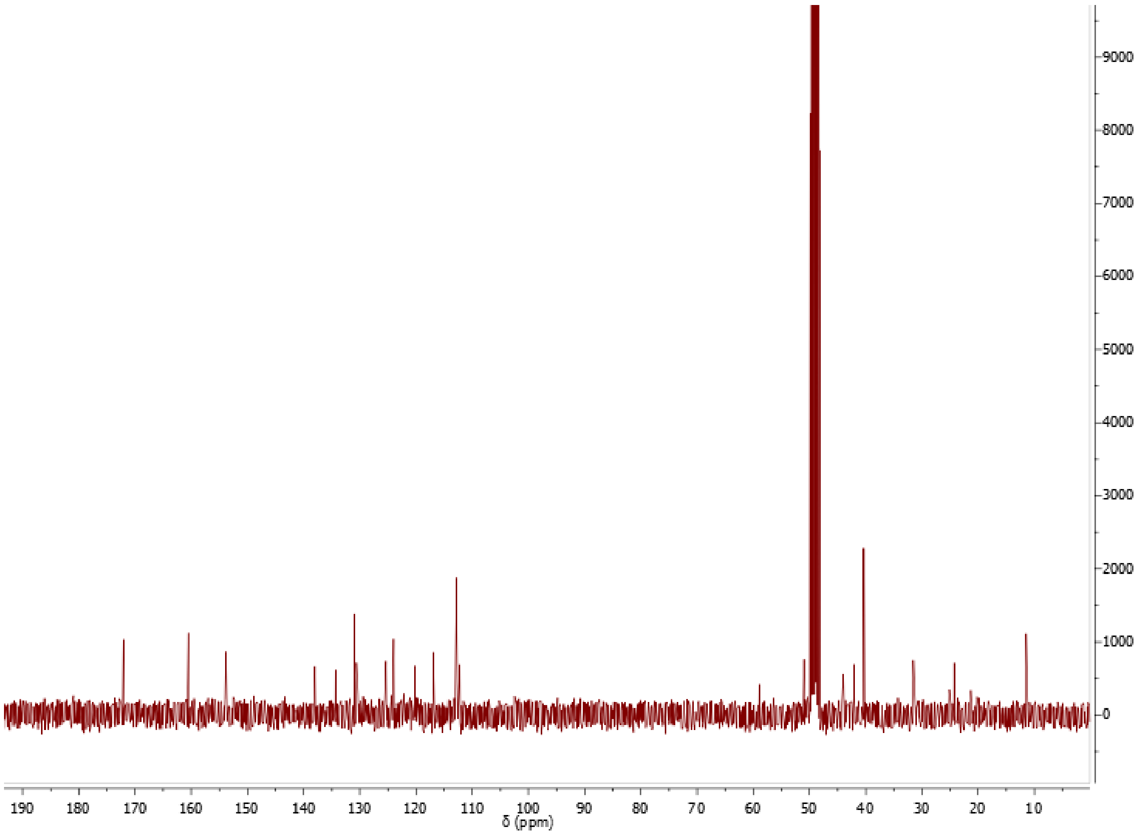

Figure S27. ^{13}C NMR spectrum of compound **12** (75 MHz, MeOD).

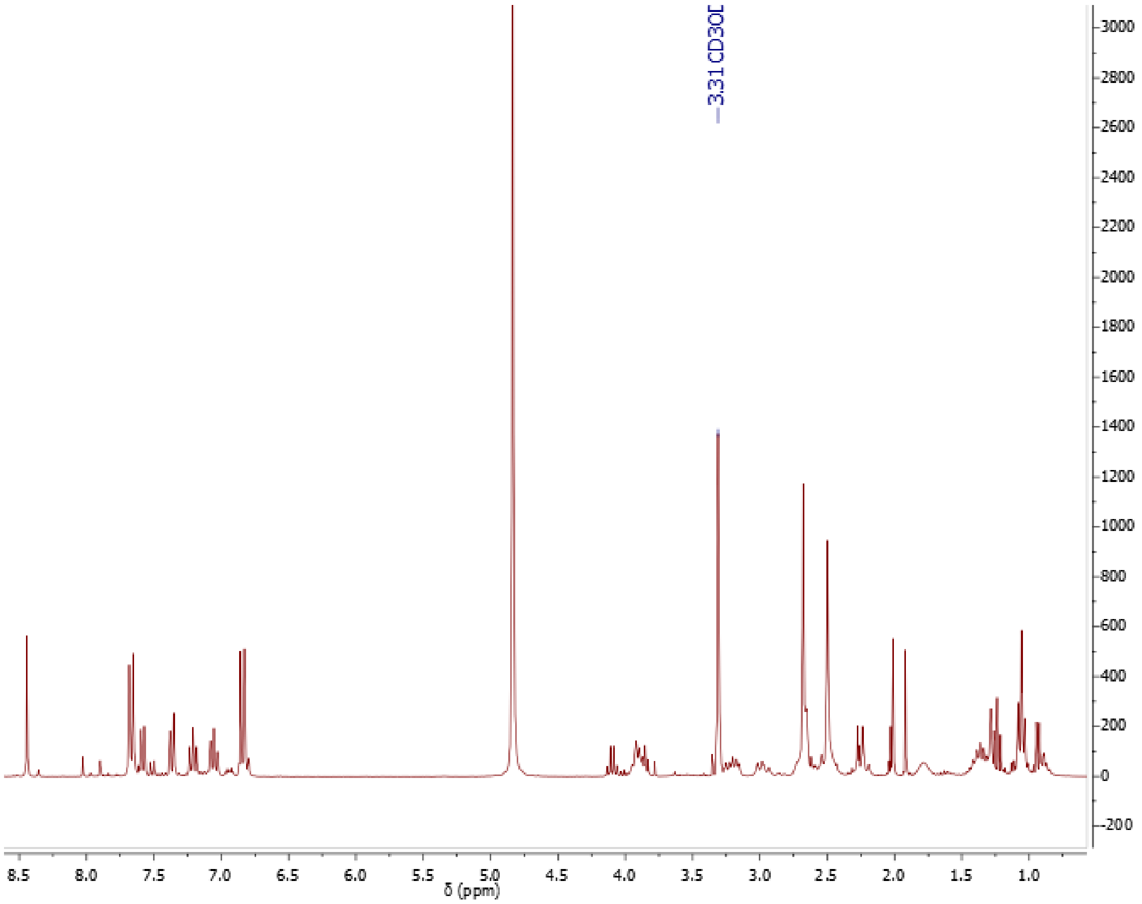

Figure S28. ^{1}H NMR spectrum of compound **13** (300 MHz, MeOD).

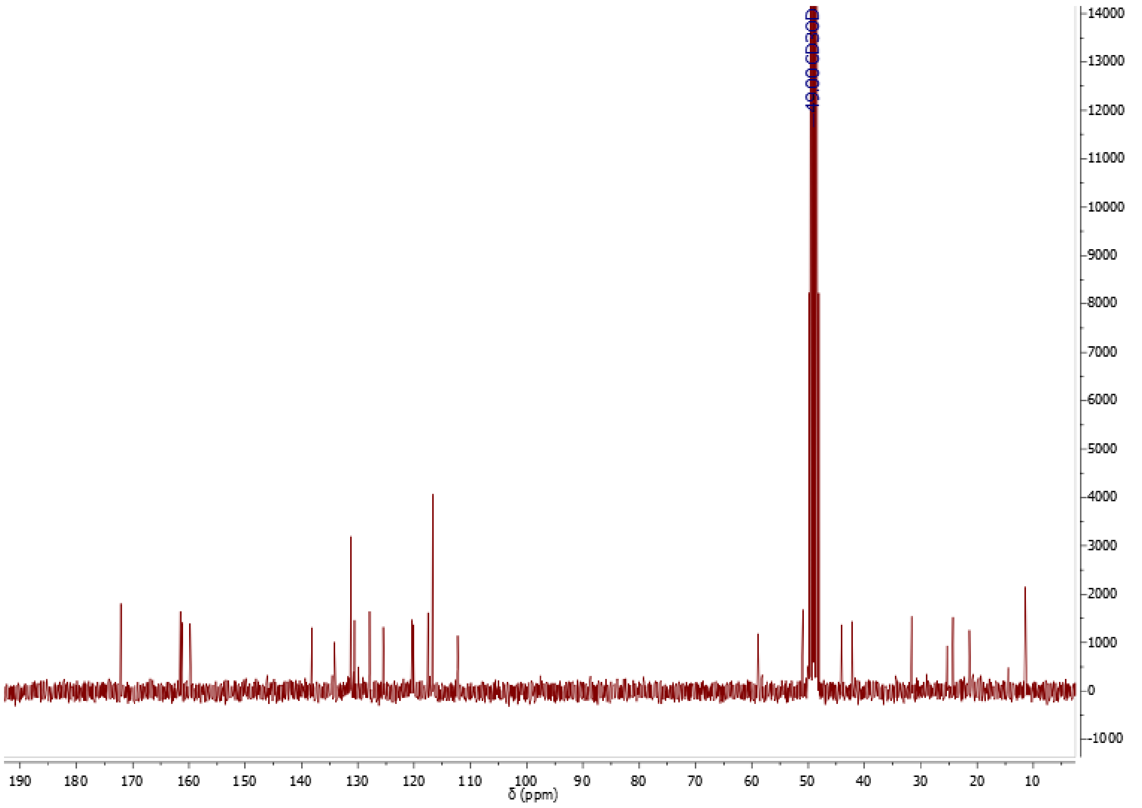

Figure S29. ^{13}C NMR spectrum of compound **13** (75 MHz, MeOD).

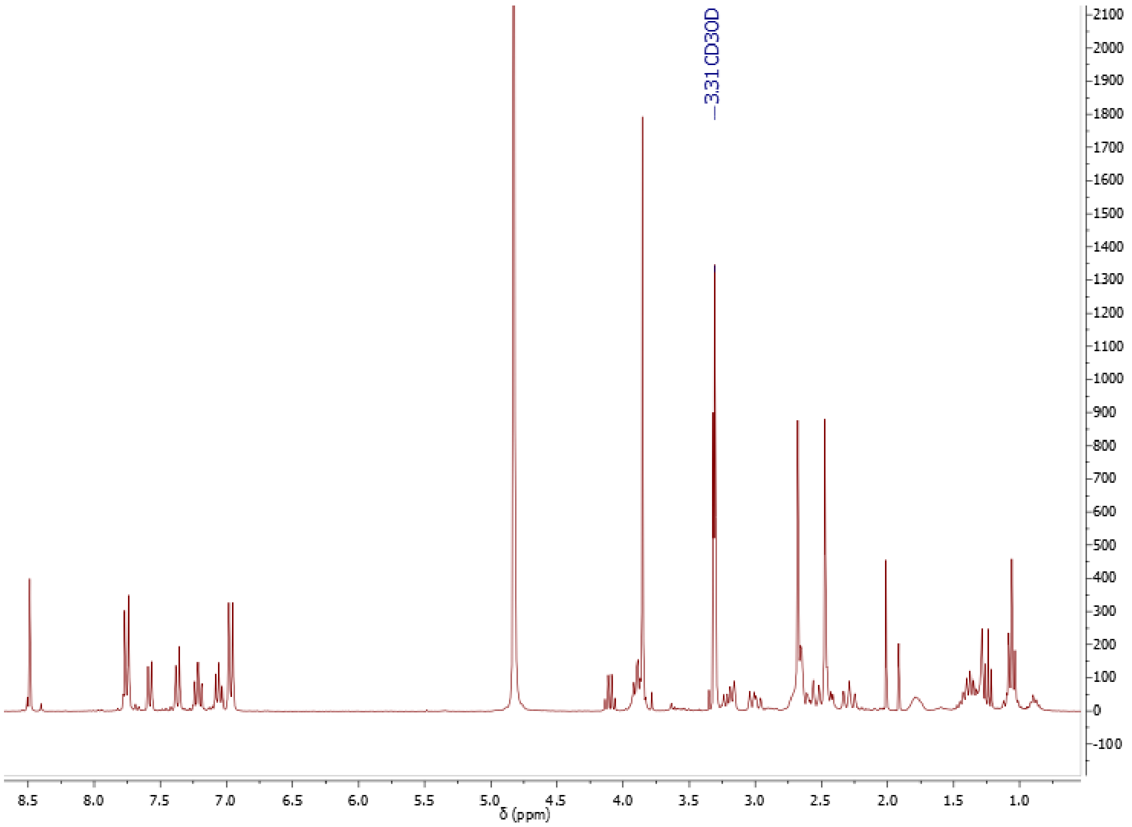

Figure S30. ^{1}H NMR spectrum of compound **14** (300 MHz, MeOD).

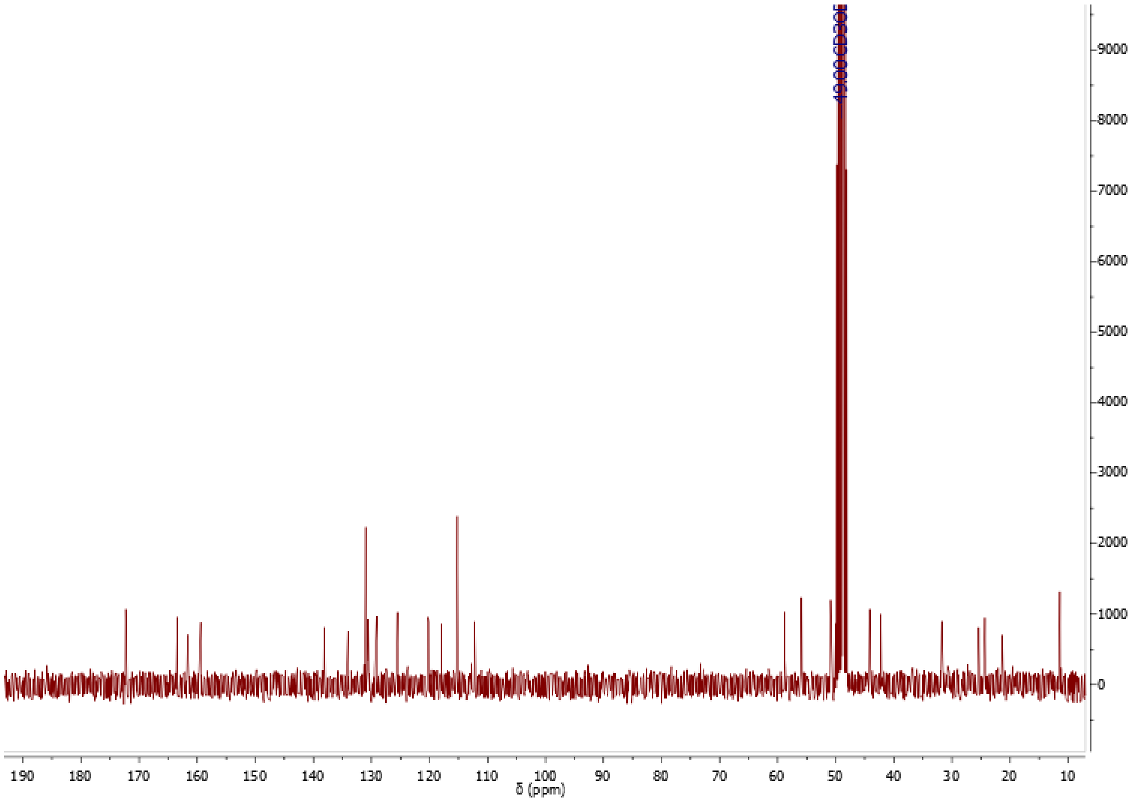

Figure S31. ^{13}C NMR spectrum of compound **14** (75 MHz, MeOD).

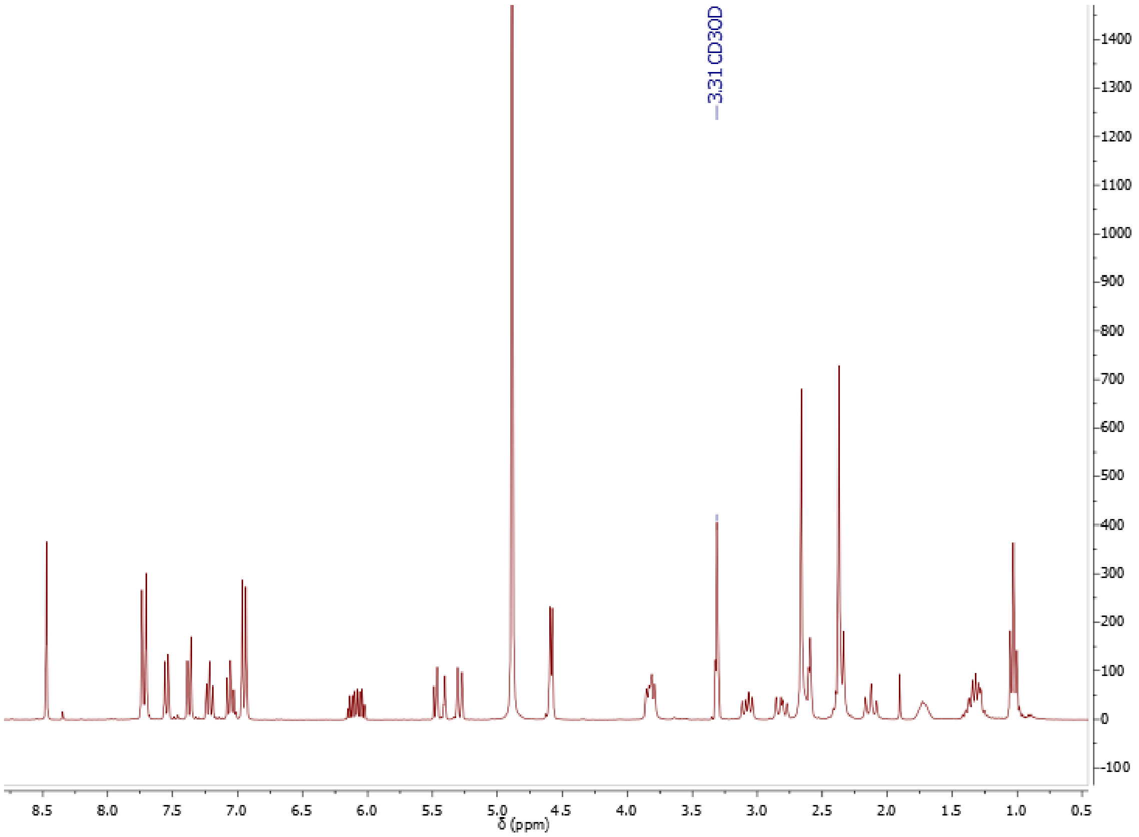

Figure S32. ^{1}H NMR spectrum of compound **15** (300 MHz, MeOD).

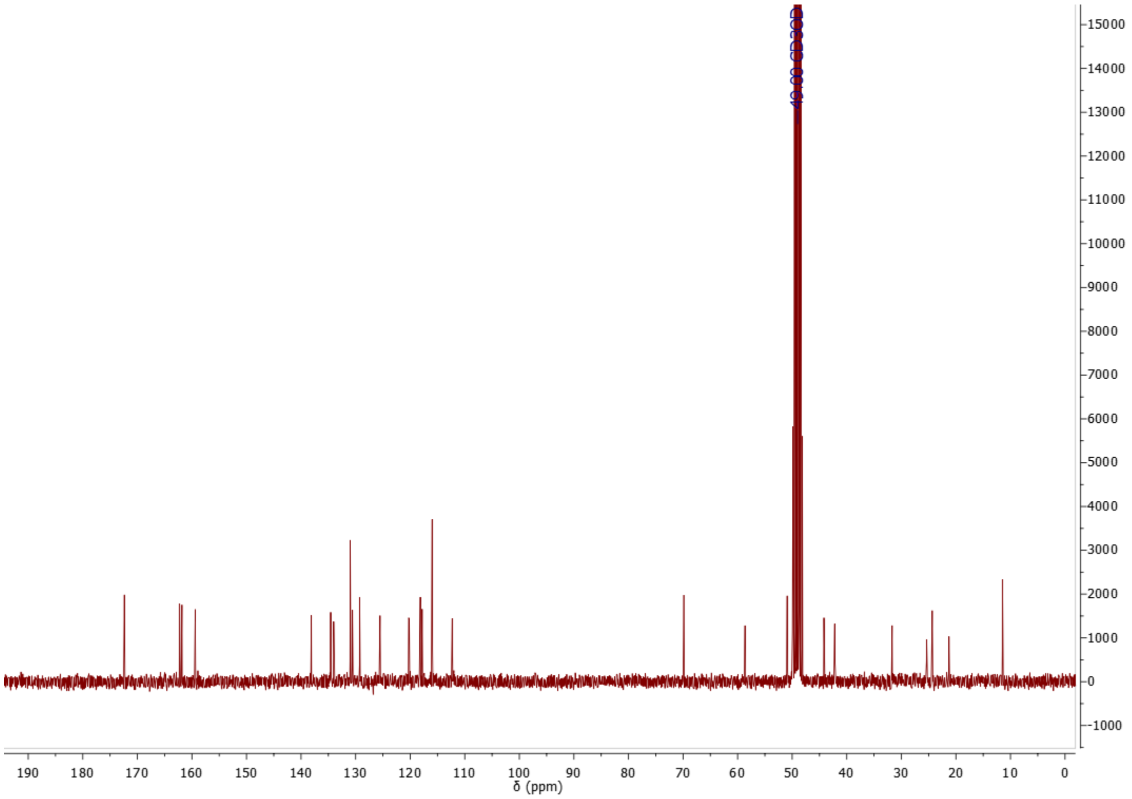

Figure S33. ^{13}C NMR spectrum of compound **15** (75 MHz, MeOD).

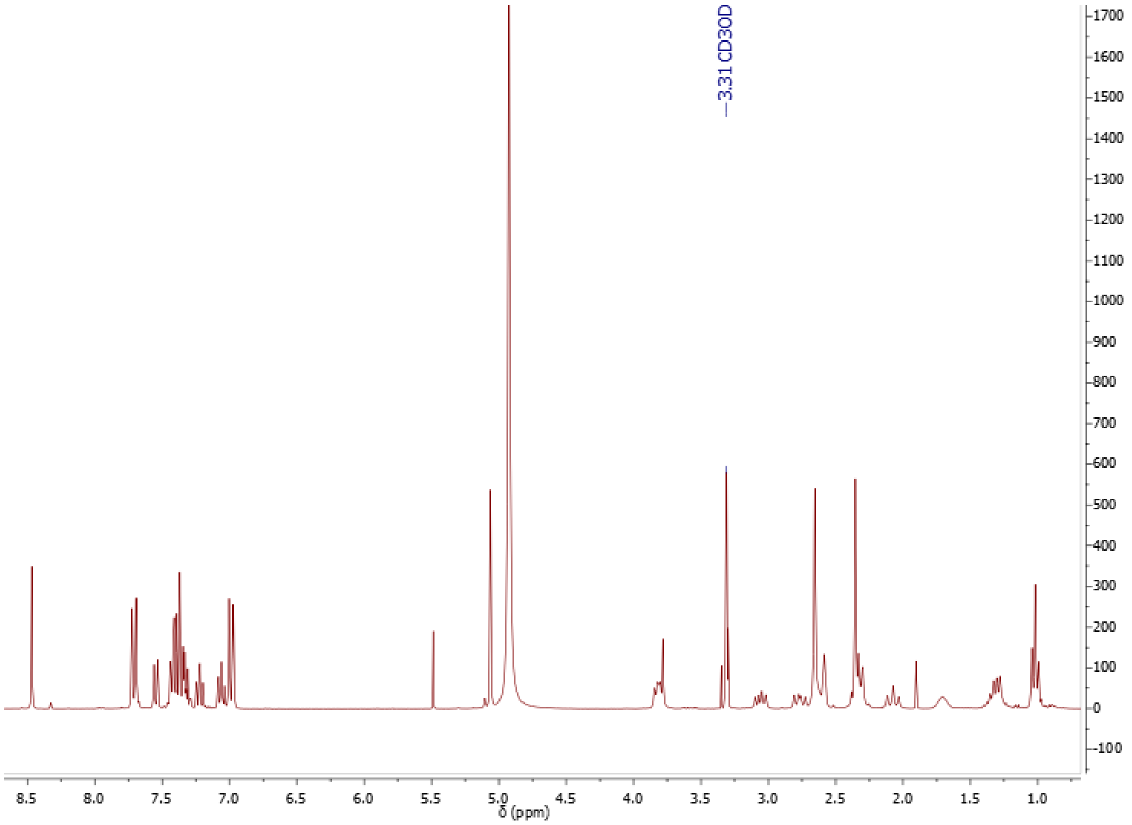

Figure S34. ^{1}H NMR spectrum of compound **16** (300 MHz, MeOD).

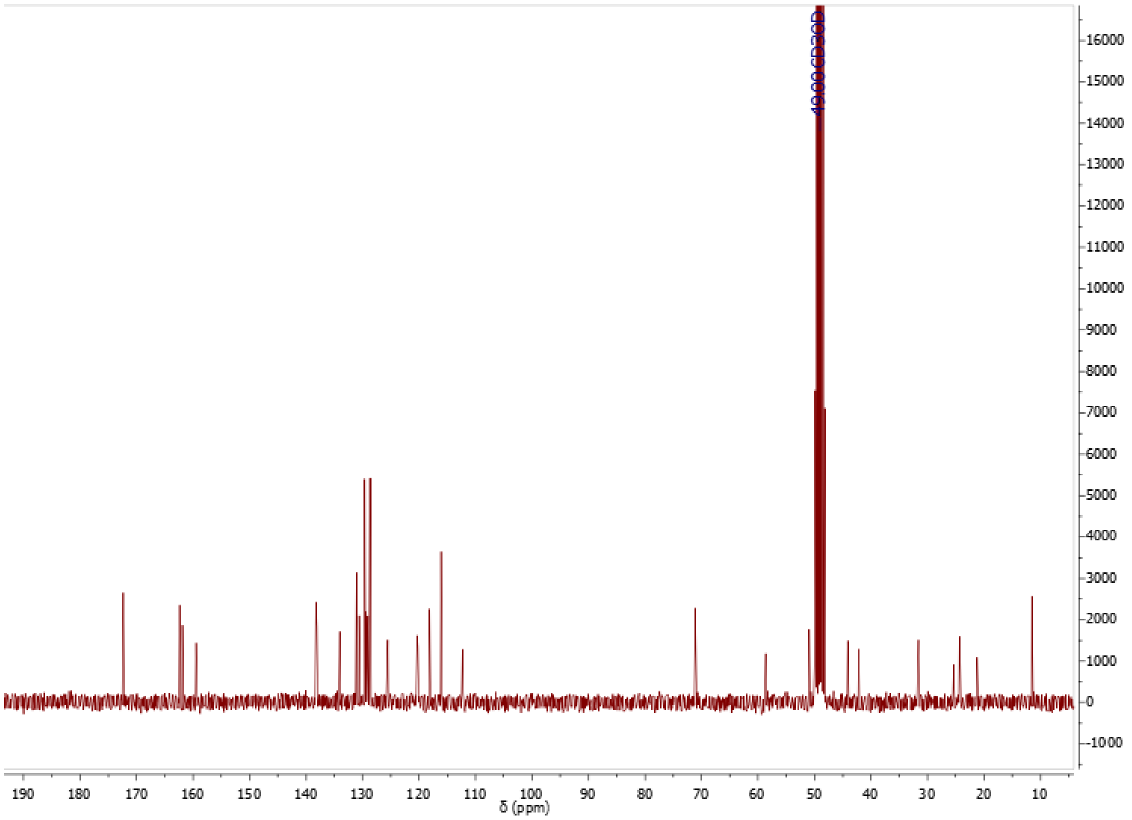

Figure S35. ^{13}C NMR spectrum of compound **16** (75 MHz, MeOD).

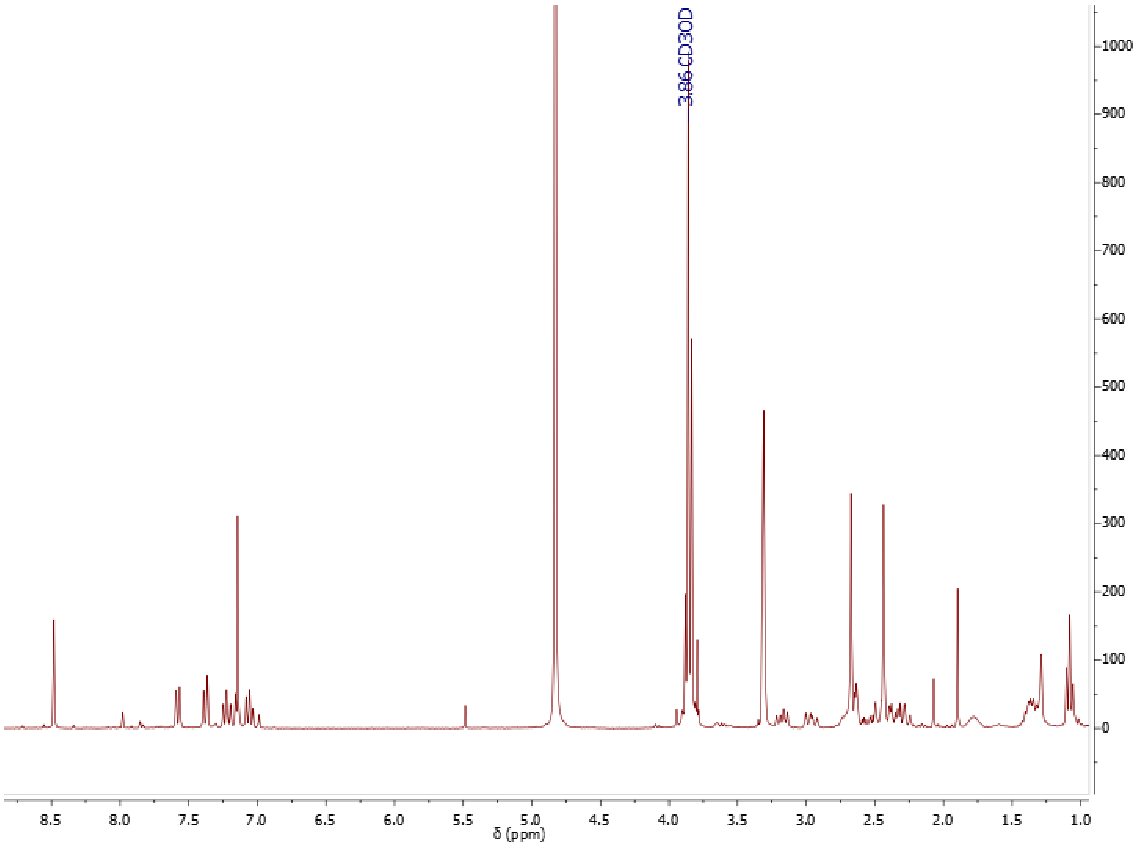

Figure S36. ^{1}H NMR spectrum of compound **17** (300 MHz, MeOD).

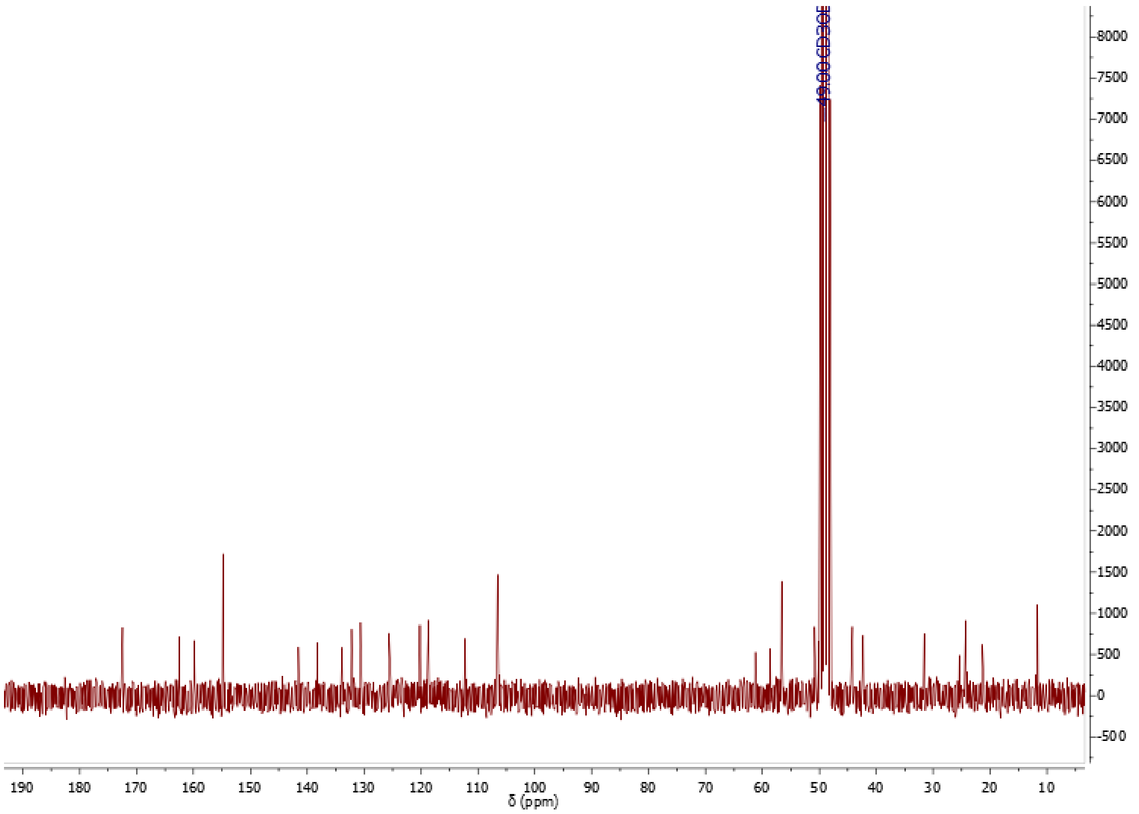

Figure S37. ^{13}C NMR spectrum of compound **17** (75 MHz, MeOD).

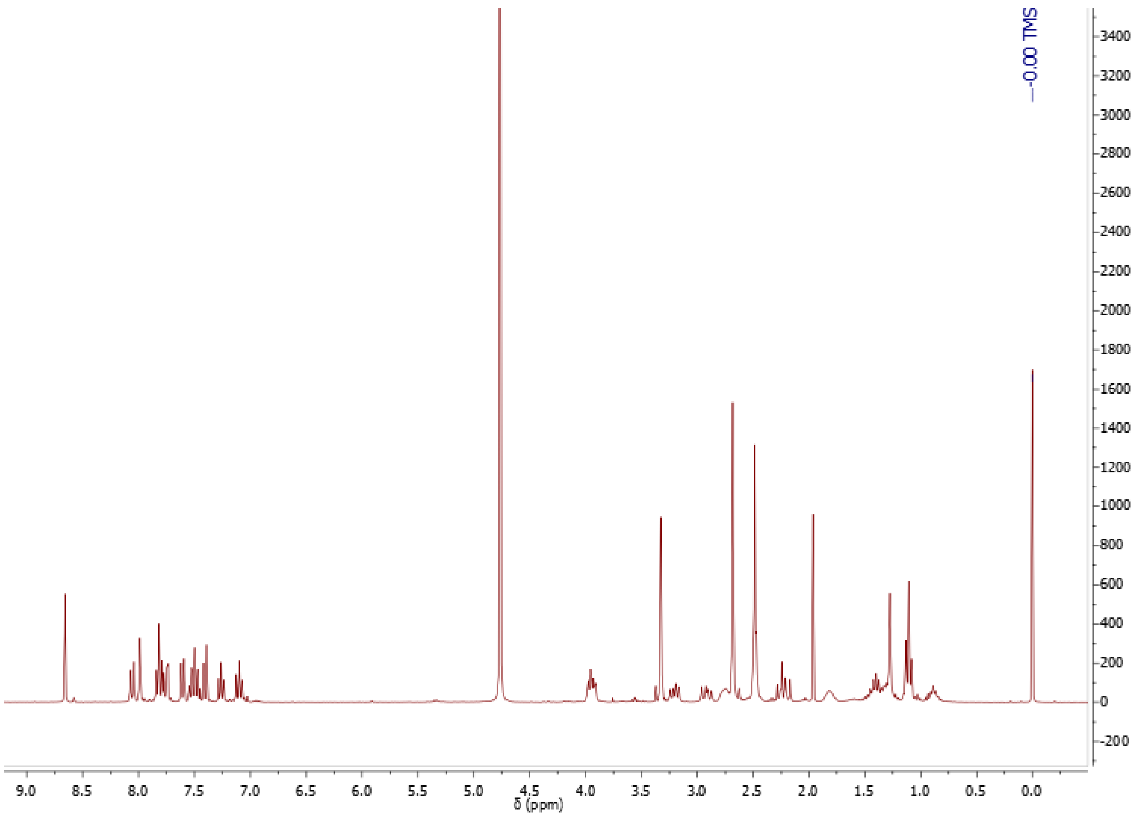

Figure S38. ^{1}H NMR spectrum of compound **18** (300 MHz, MeOD + $CDCl_3$).

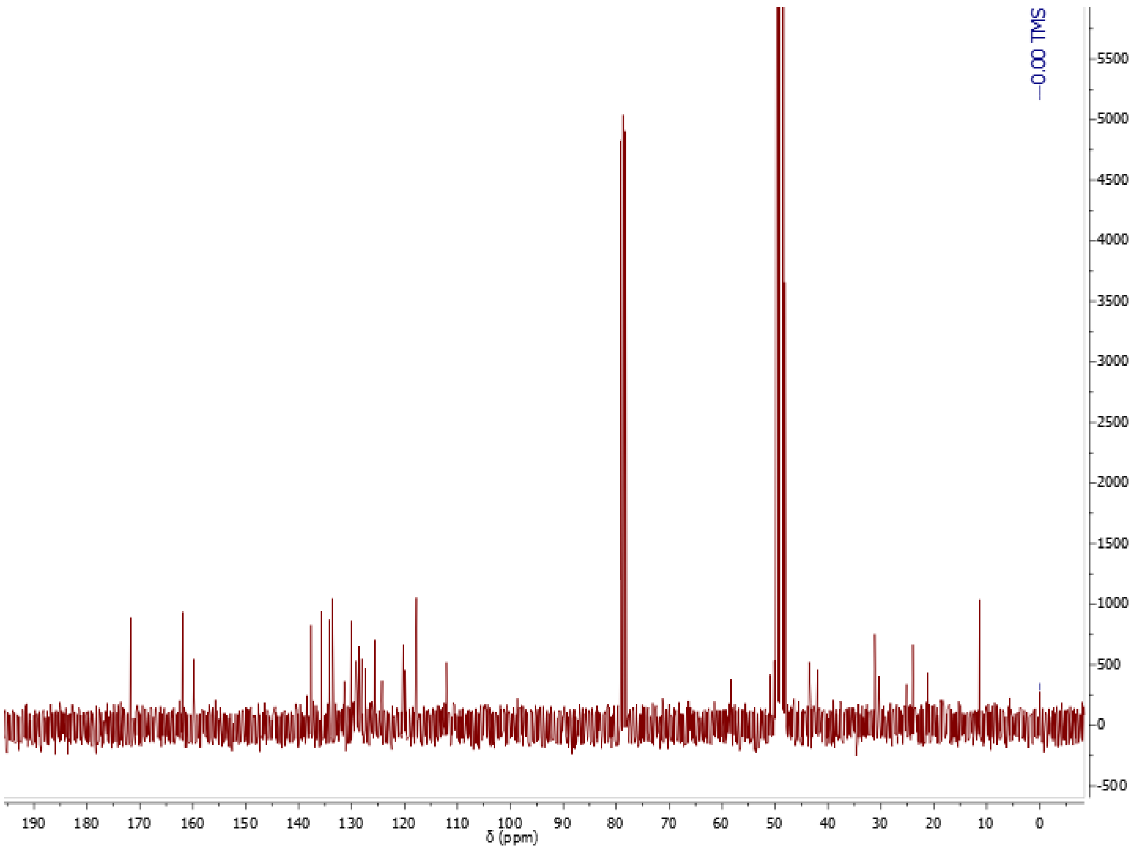

Figure S39. ^{13}C NMR spectrum of compound **18** (75 MHz, MeOD + $CDCl_3$).

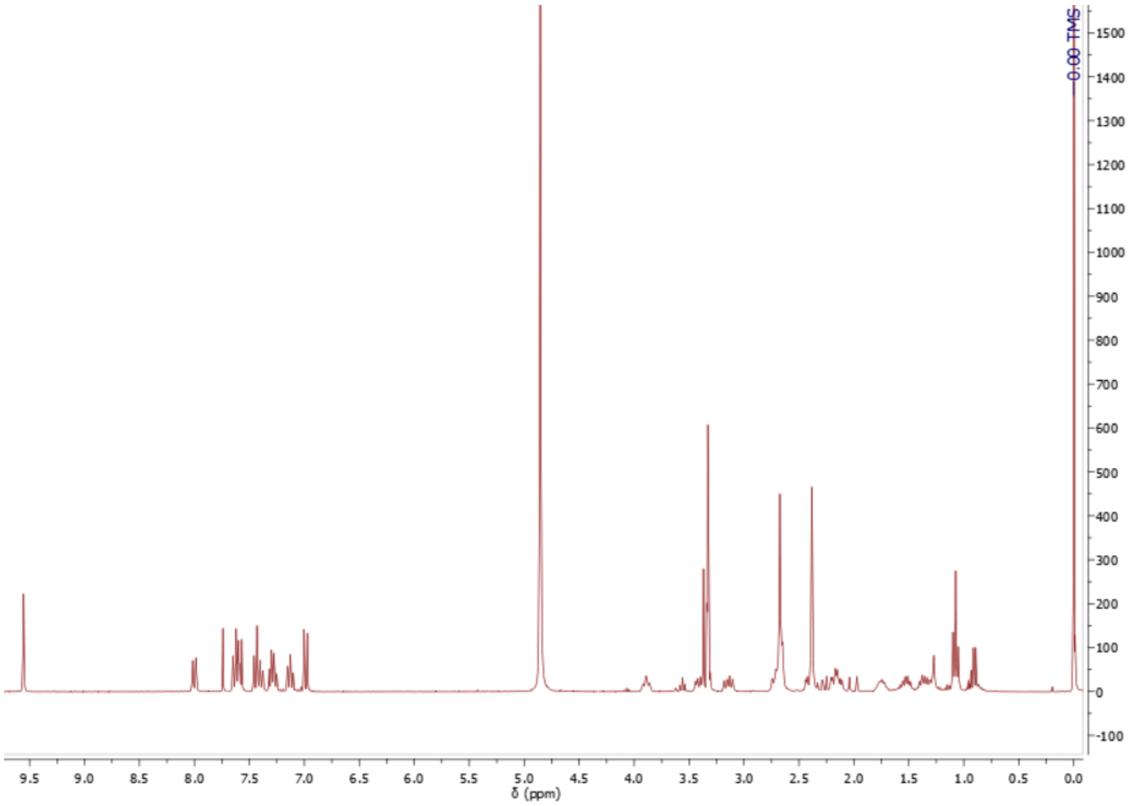

Figure S40. ^{1}H NMR spectrum of compound **19** (300 MHz, $CDCl_3$ + MeOD).

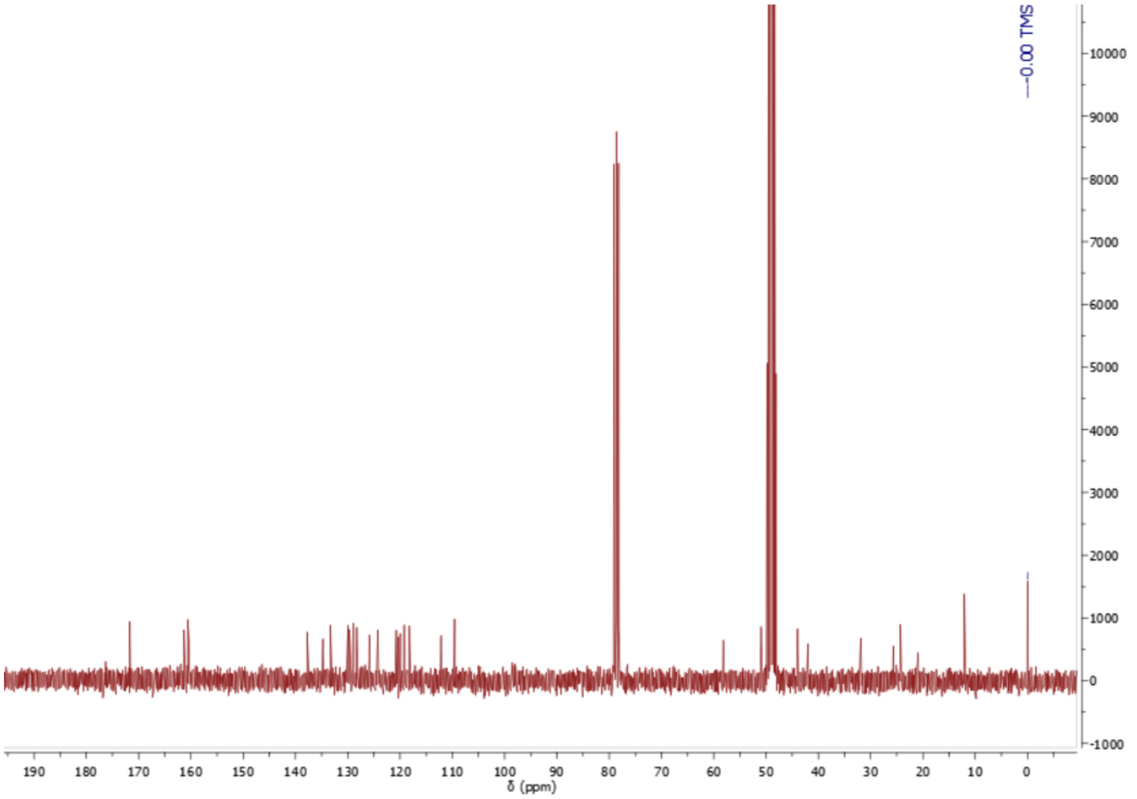

Figure S41. ^{13}C NMR spectrum of compound **19** (75 MHz, $CDCl_3$ + MeOD).

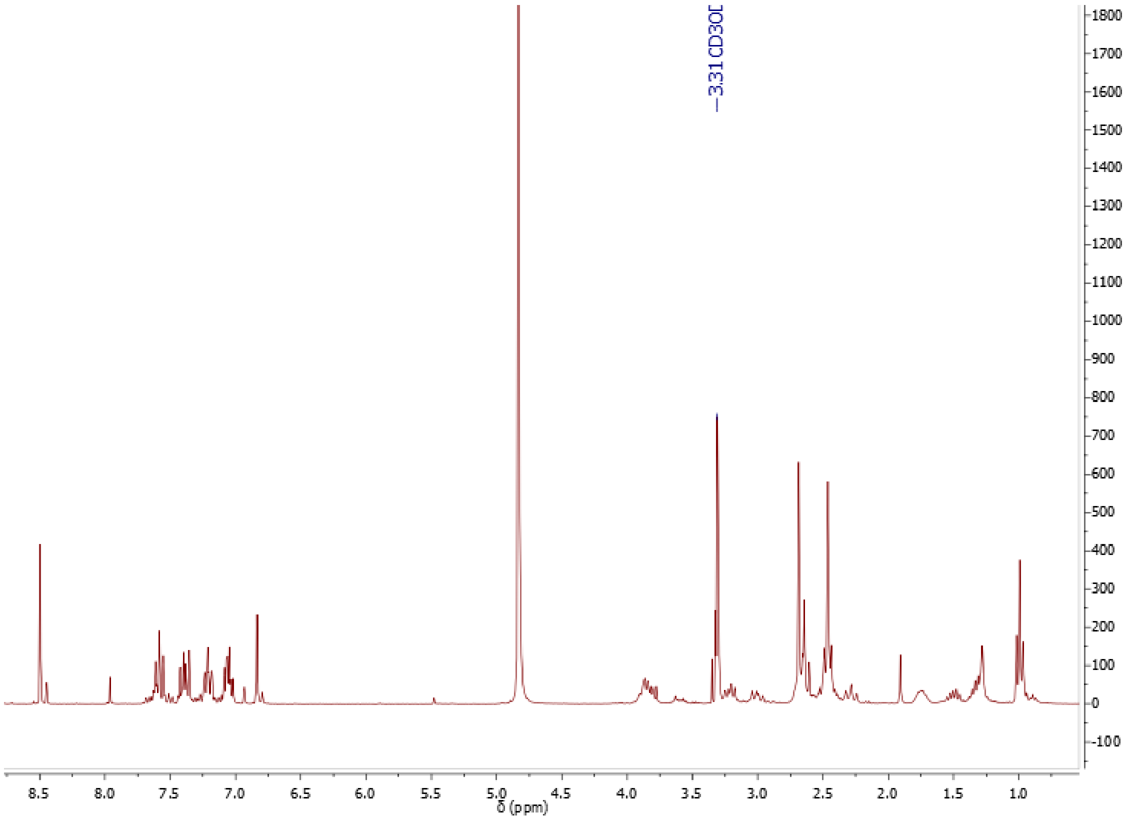

Figure S42. ^{1}H NMR spectrum of compound **20** (300 MHz, MeOD).

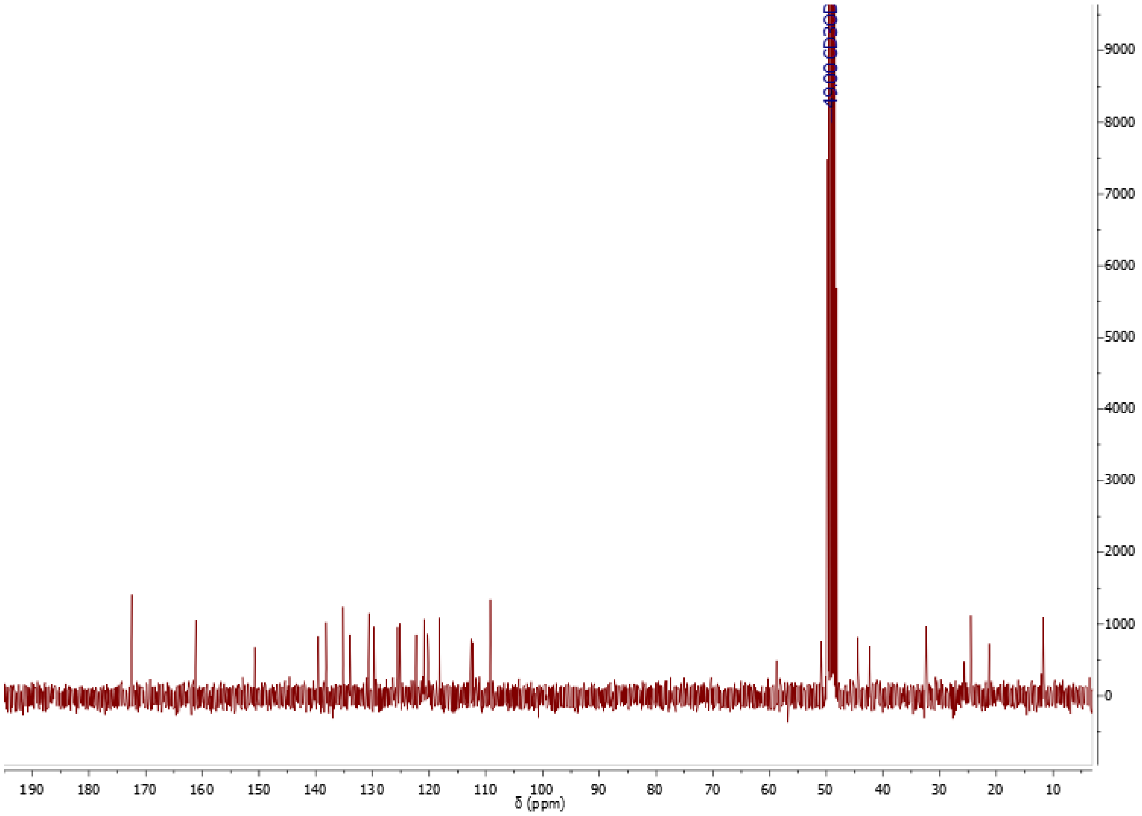

Figure S43. ^{13}C NMR spectrum of compound **20** (75 MHz, MeOD).

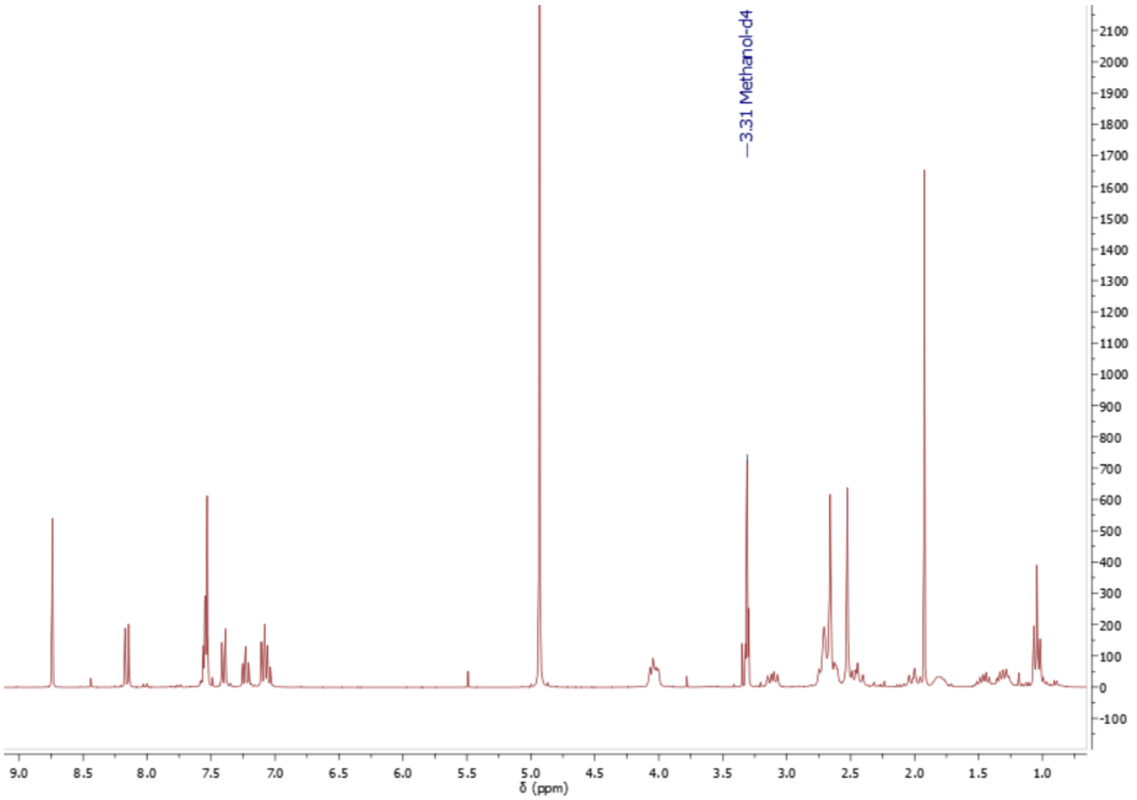

Figure S44. ^{1}H NMR spectrum of compound **21** (300 MHz, MeOD).

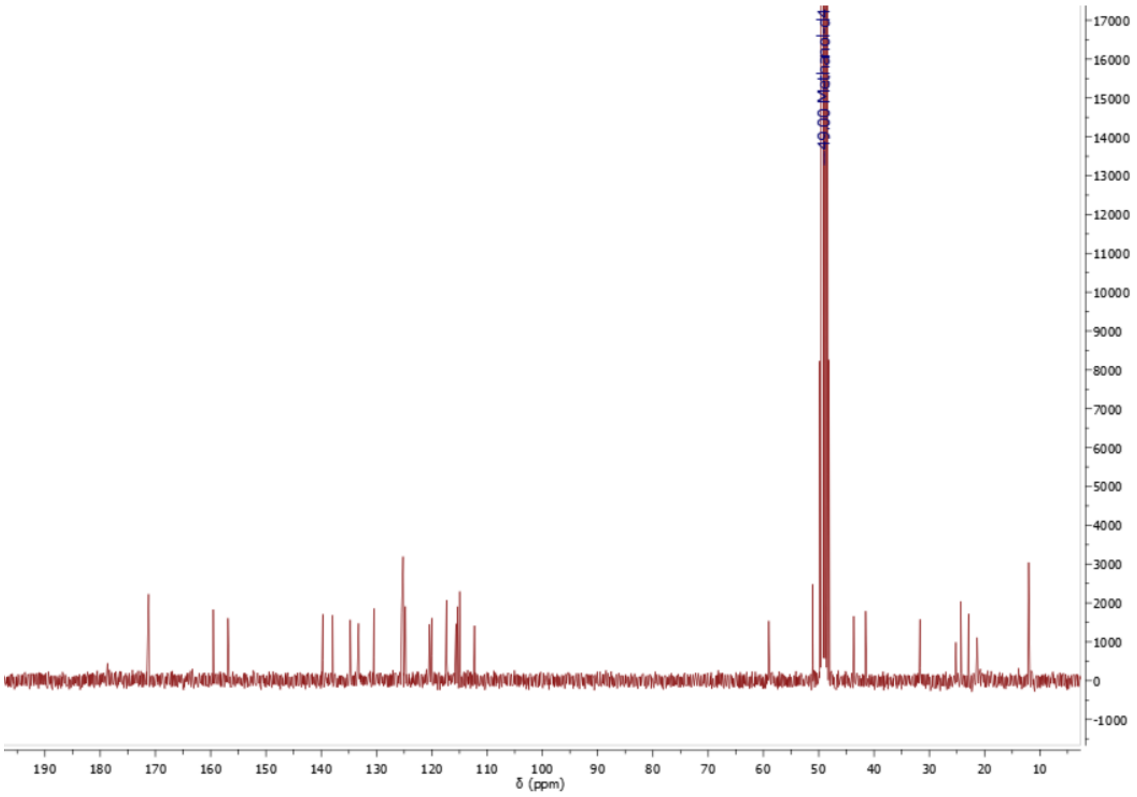

Figure S45. ^{13}C NMR spectrum of compound **21** (75 MHz, MeOD).

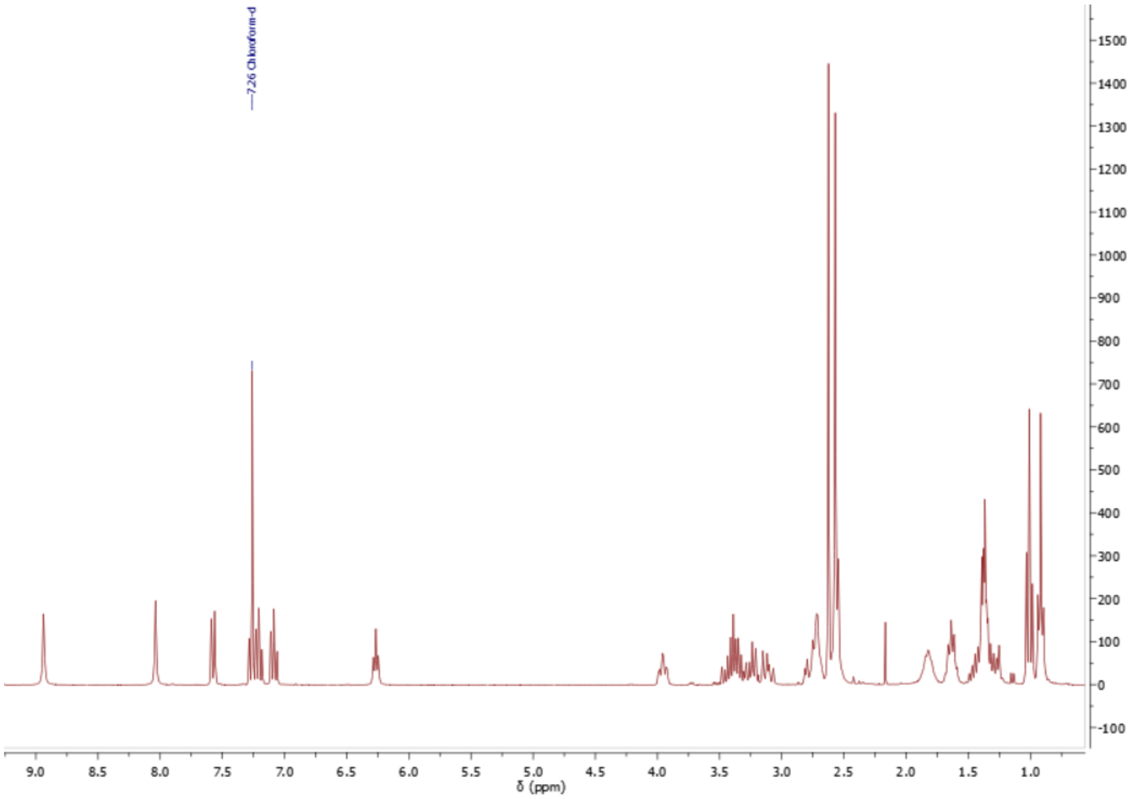

Figure S46. ^{1}H NMR spectrum of compound **22** (300 MHz, $CDCl_3$).

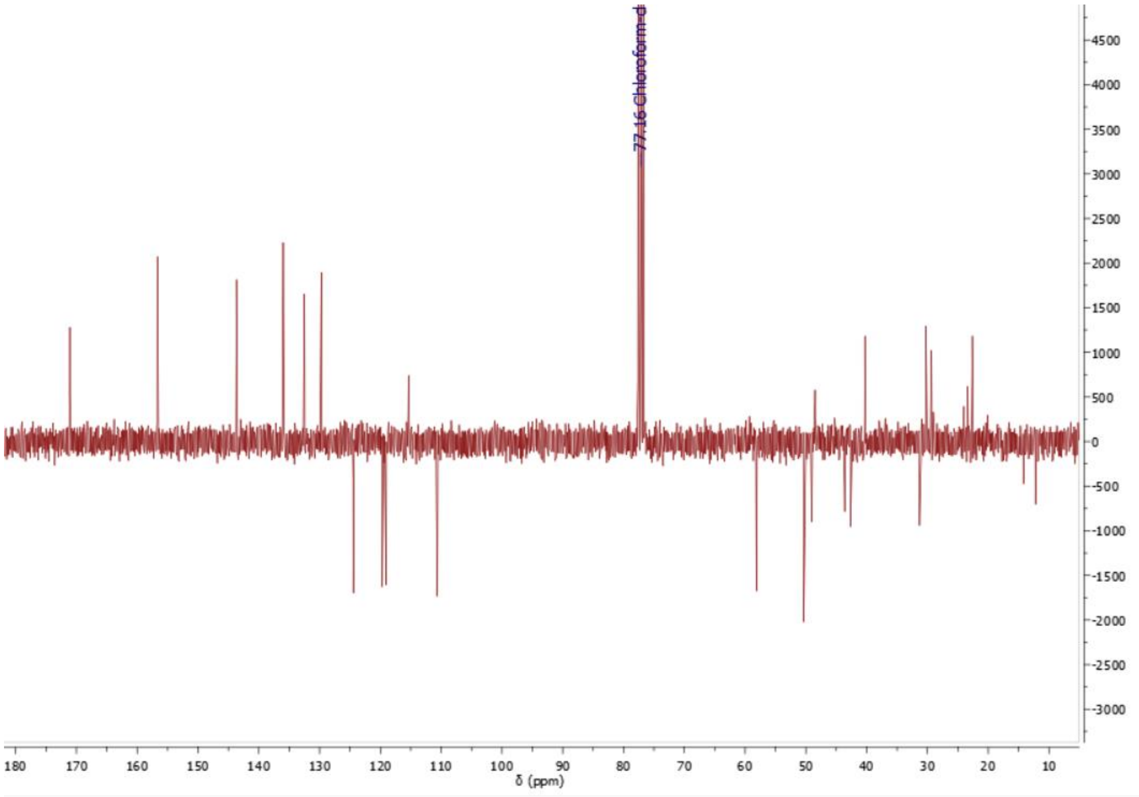

Figure S47. ^{13}C (APT) NMR spectrum of compound **22** (75 MHz, $CDCl_3$).

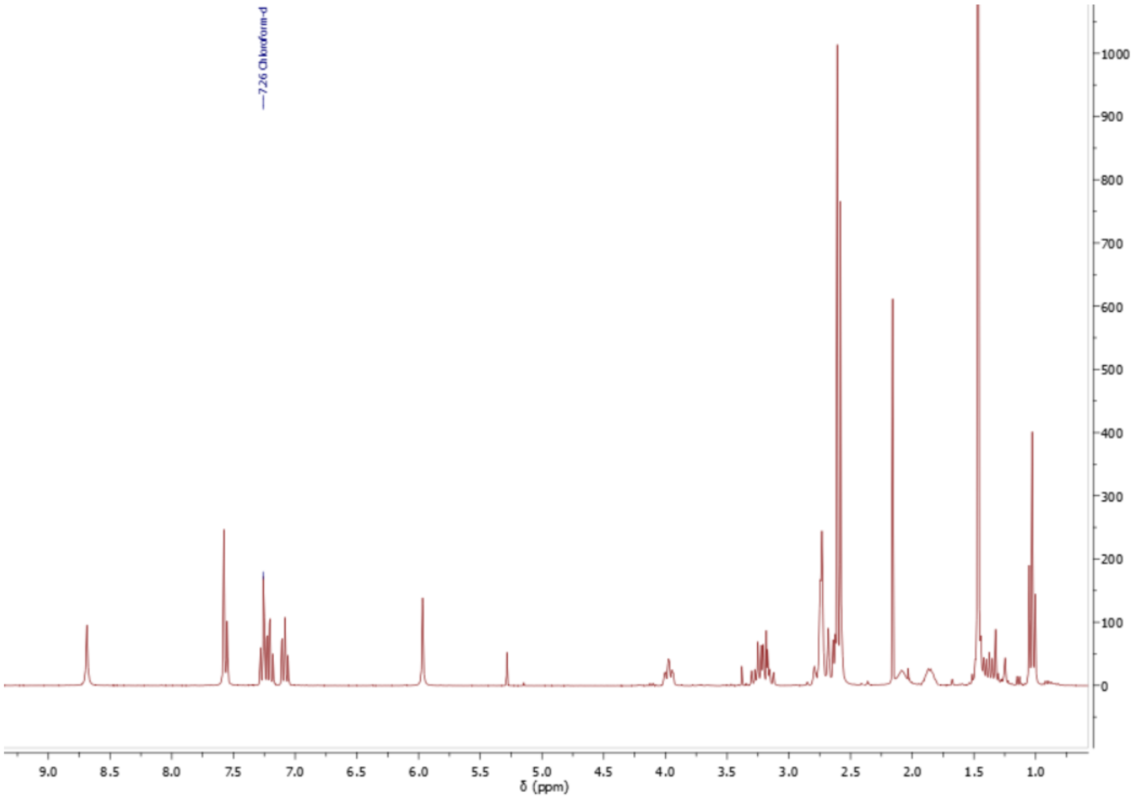

Figure S48. ^{1}H NMR spectrum of compound **23** (300 MHz, $CDCl_3$).

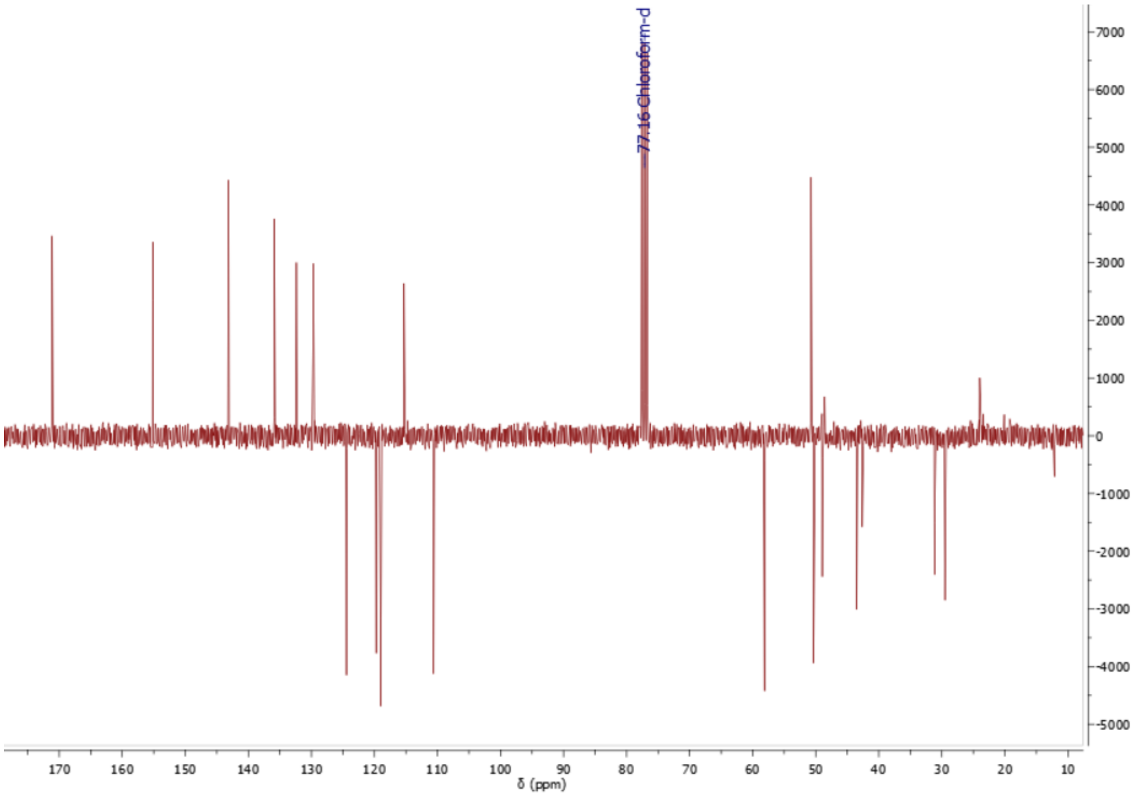

Figure S49. ^{13}C (APT) NMR spectrum of compound **23** (75 MHz, $CDCl_3$).

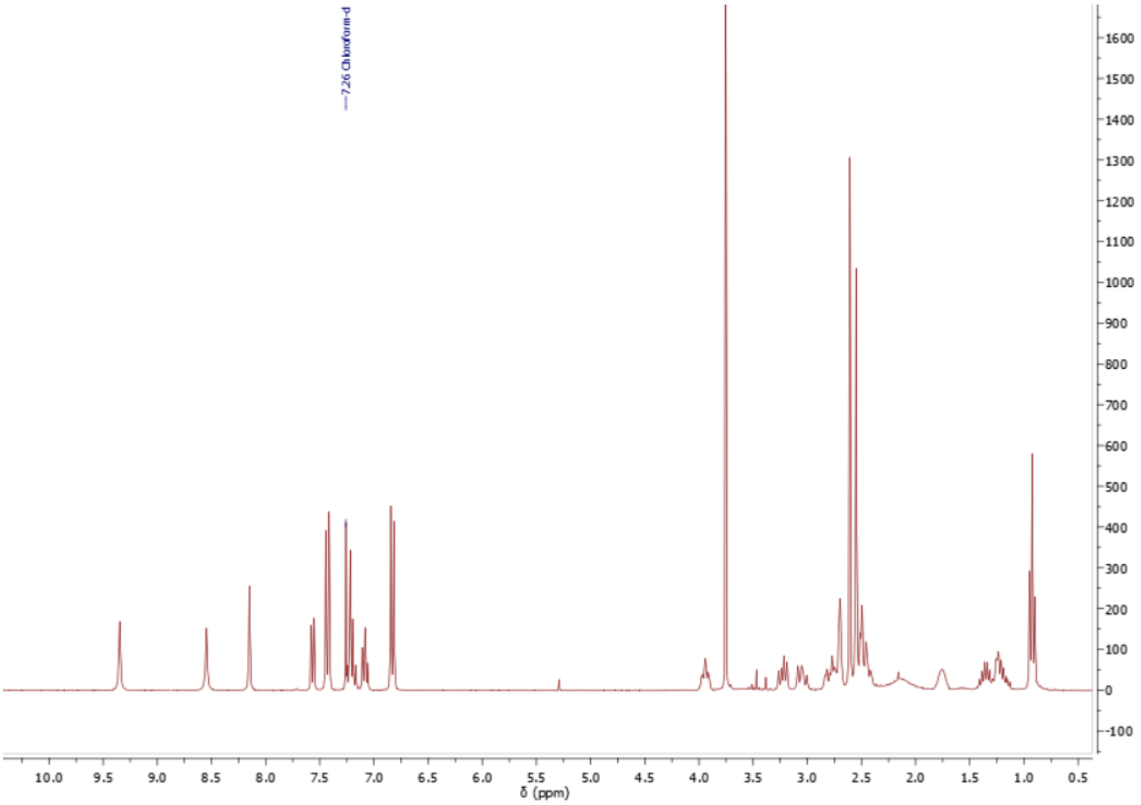

Figure S50. ^{1}H NMR spectrum of compound **24** (300 MHz, $CDCl_3$).

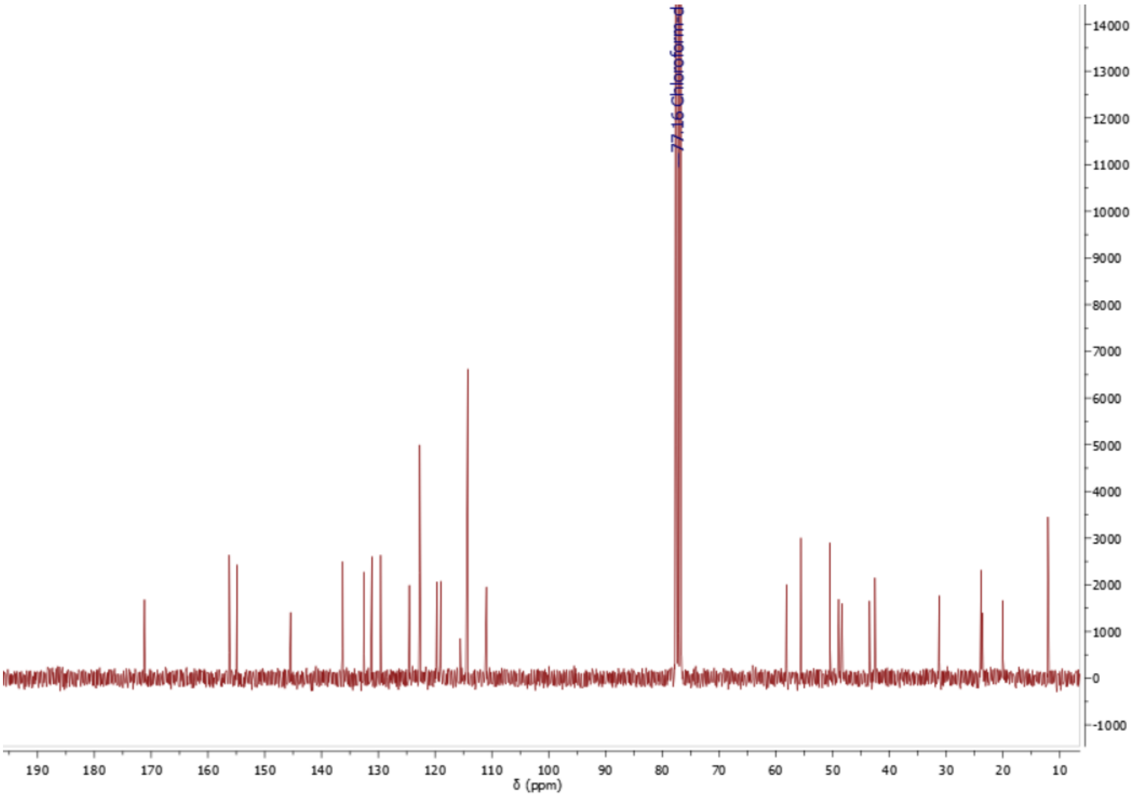

Figure S51. ^{13}C NMR spectrum of compound **24** (75 MHz, $CDCl_3$).

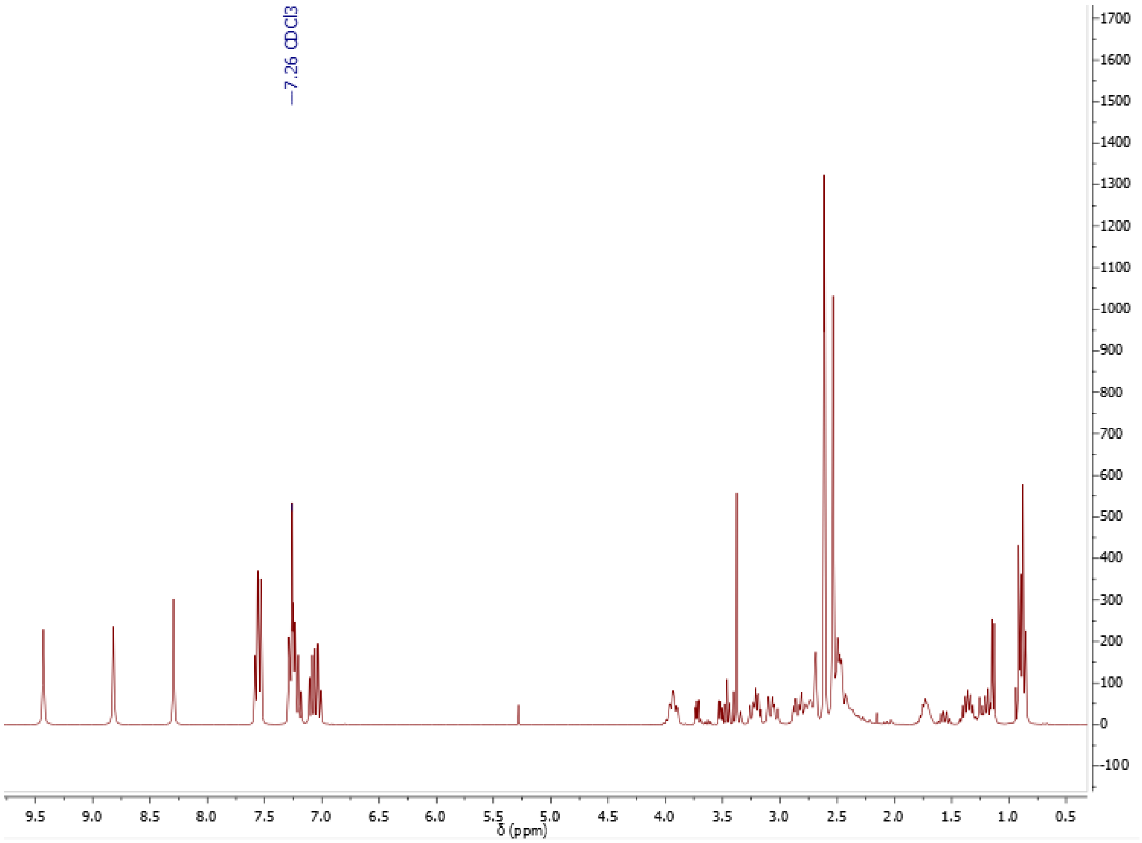

Figure S52. ^{1}H NMR spectrum of compound **25** (300 MHz, $CDCl_3$).

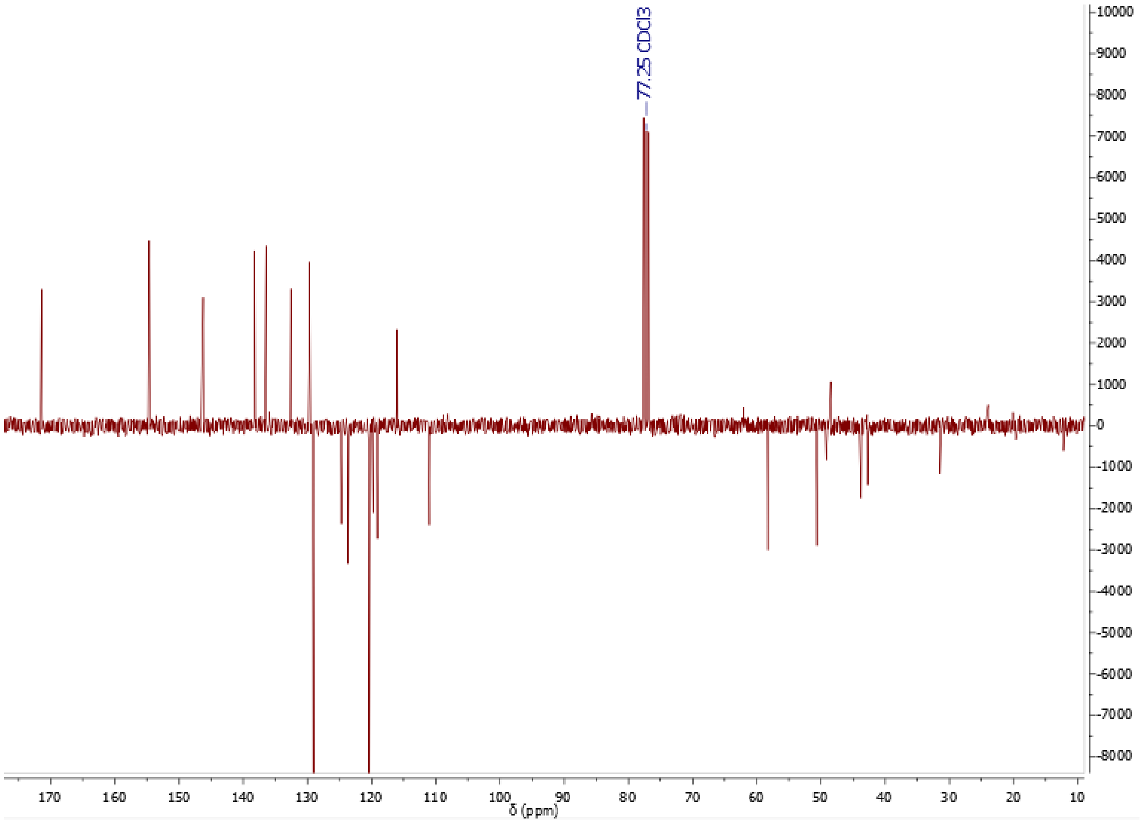

Figure S53. ^{13}C (APT) NMR spectrum of compound **25** (75 MHz, $CDCl_3$).

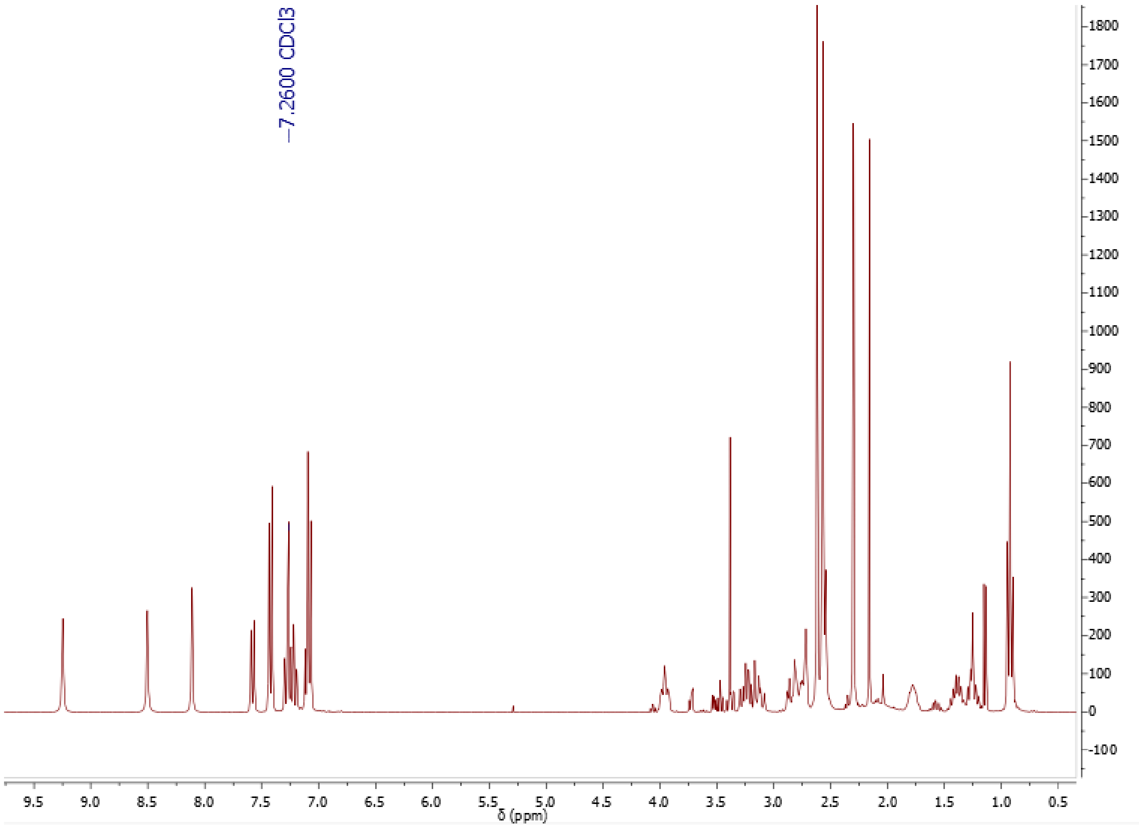

Figure S54. ^{1}H NMR spectrum of compound **26** (300 MHz, $CDCl_3$).

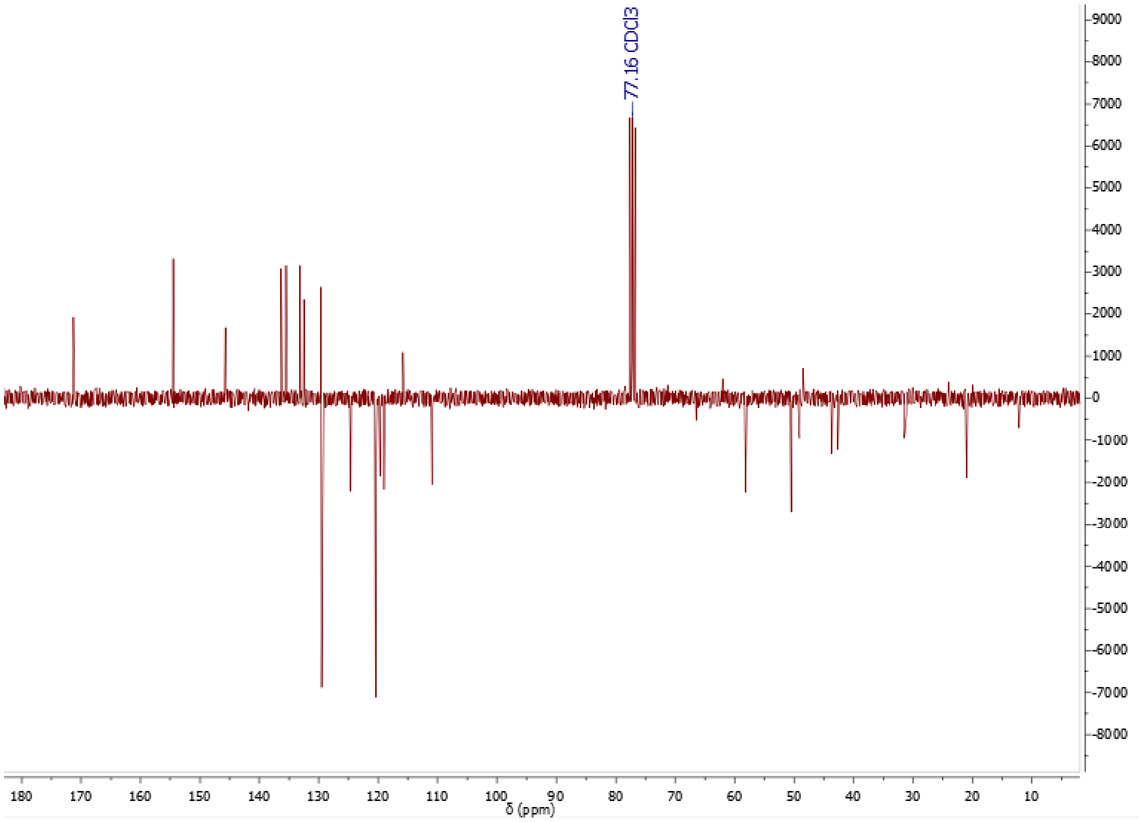

Figure S55. ^{13}C (APT) NMR spectrum of compound **26** (75 MHz, $CDCl_3$).

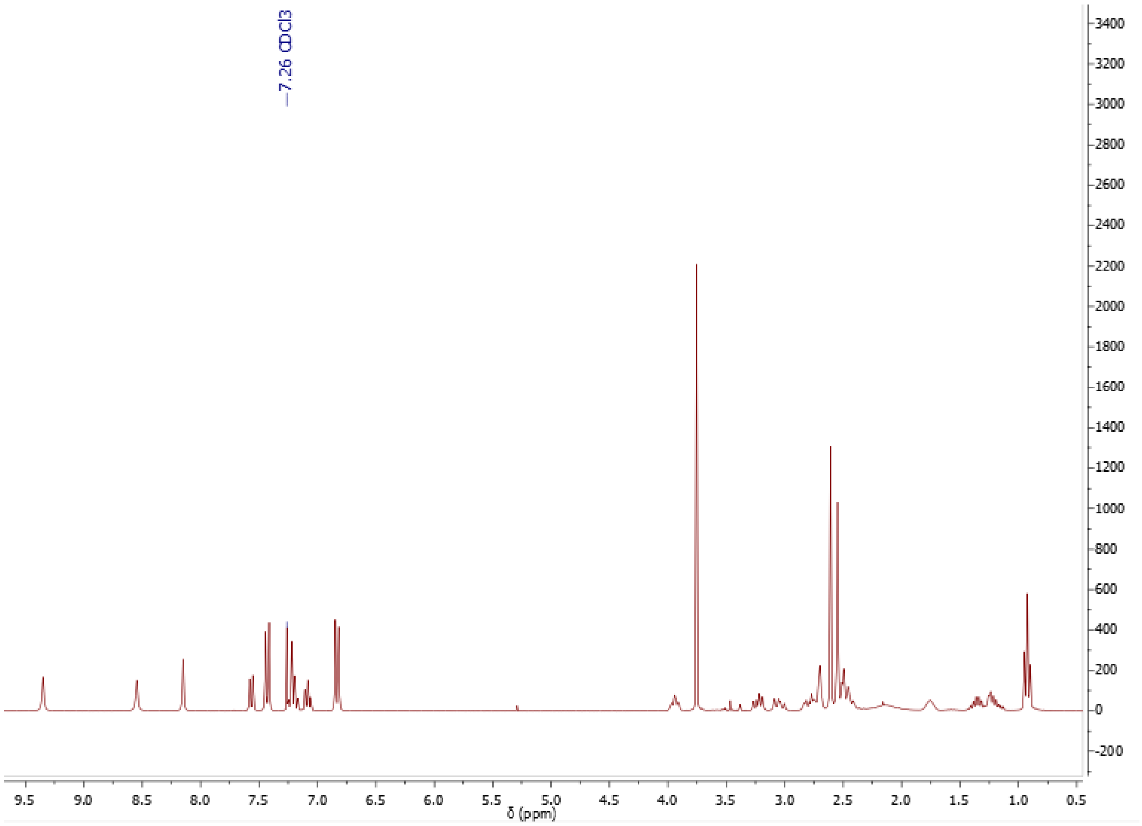

Figure S56. ^{1}H NMR spectrum of compound **27** (300 MHz, $CDCl_3$).

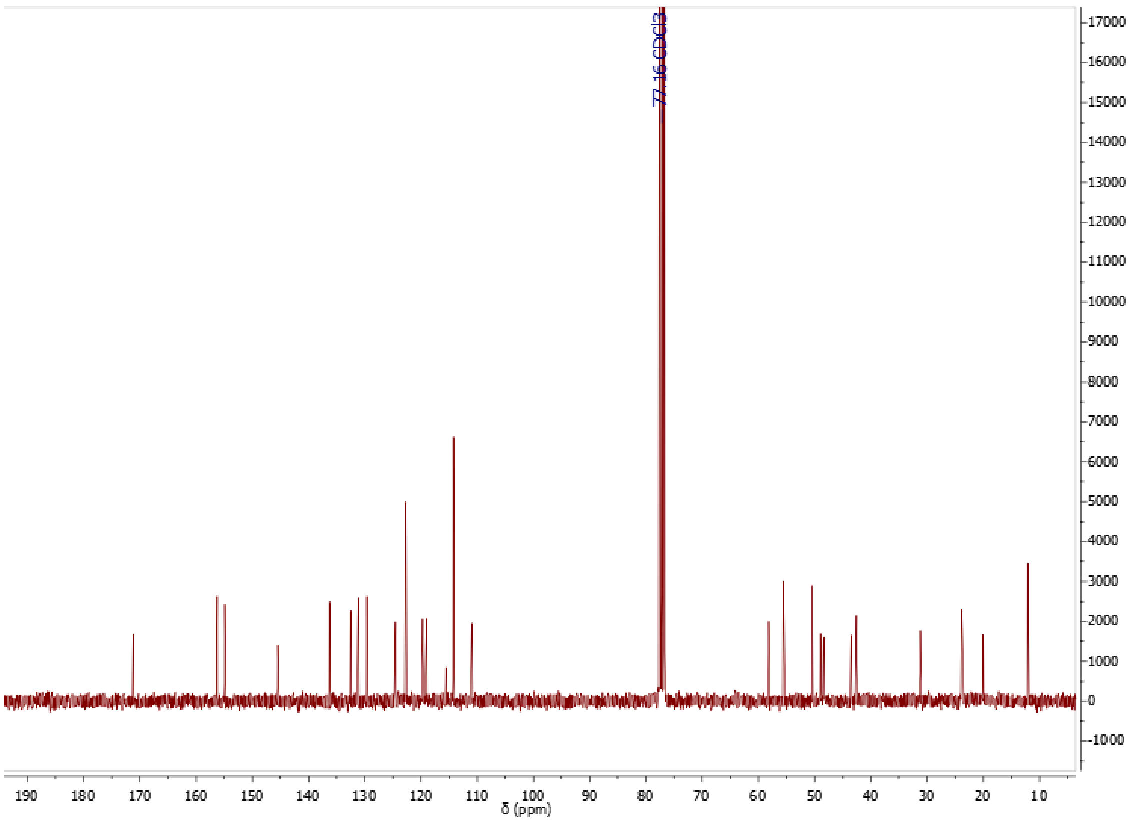

Figure S57. ^{13}C NMR spectrum of compound **27** (75 MHz, $CDCl_3$).

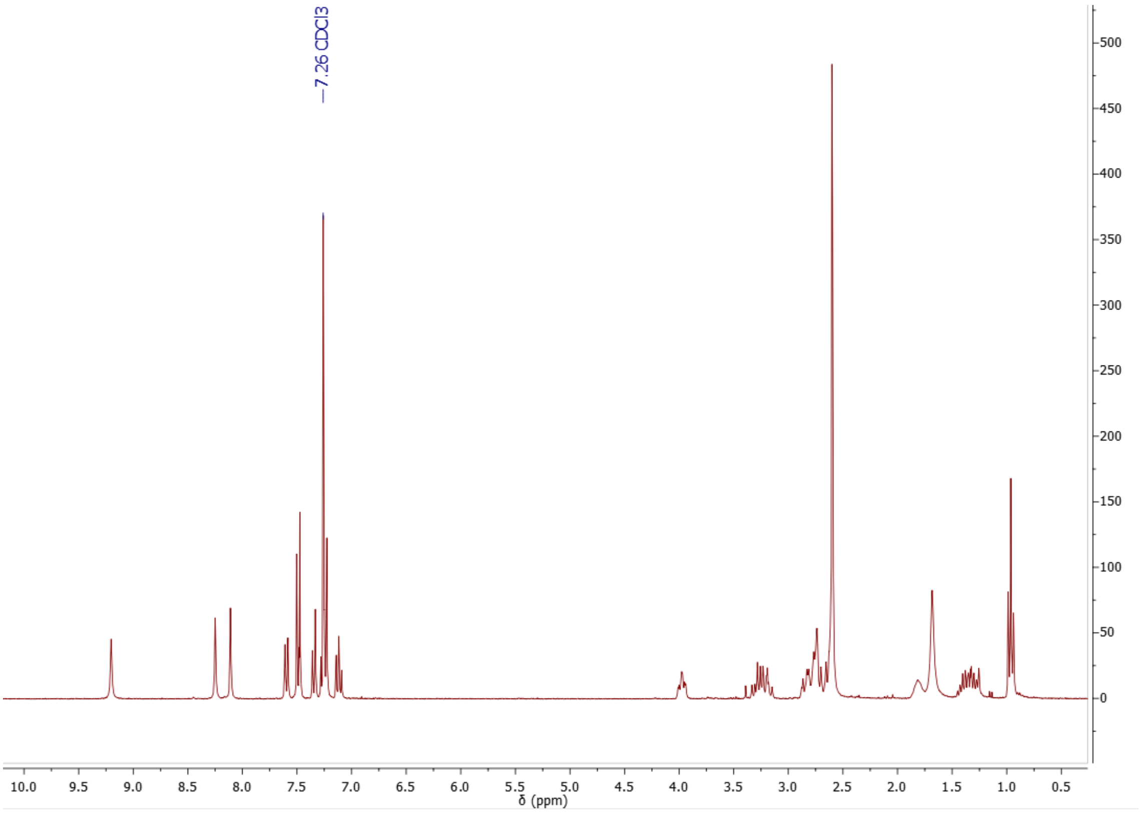

Figure S58. ^{1}H NMR spectrum of compound **28** (300 MHz, $CDCl_3$).

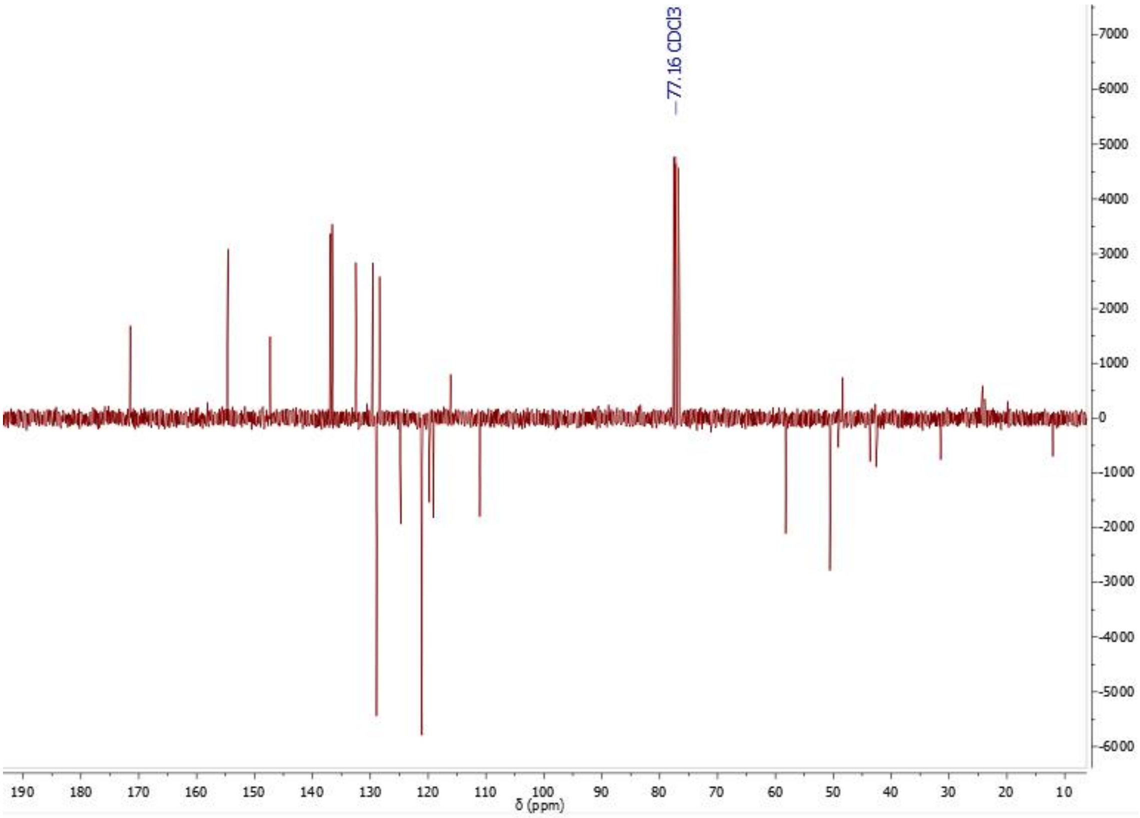

Figure S59. ^{13}C (APT) NMR spectrum of compound **28** (75 MHz, $CDCl_3$).

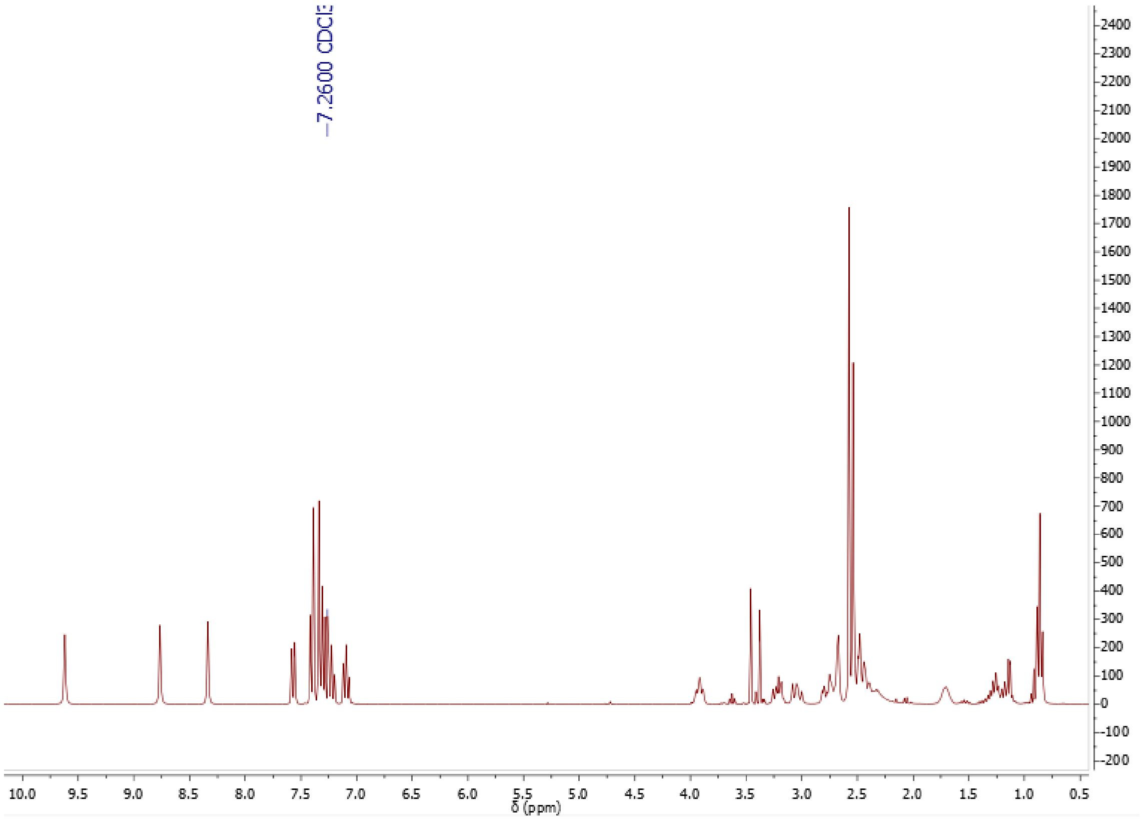

Figure S60. ^{1}H NMR spectrum of compound **29** (300 MHz, $CDCl_3$).

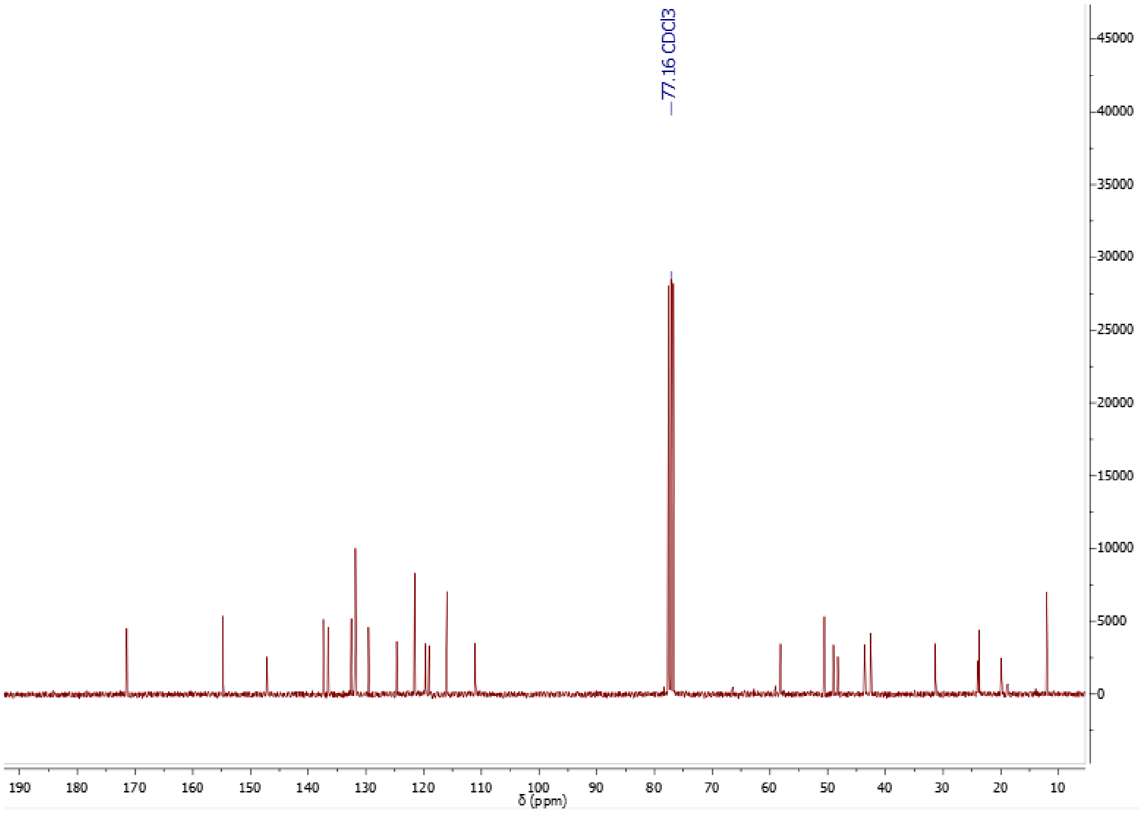

Figure S61. ^{13}C NMR spectrum of compound **29** (75 MHz, $CDCl_3$).

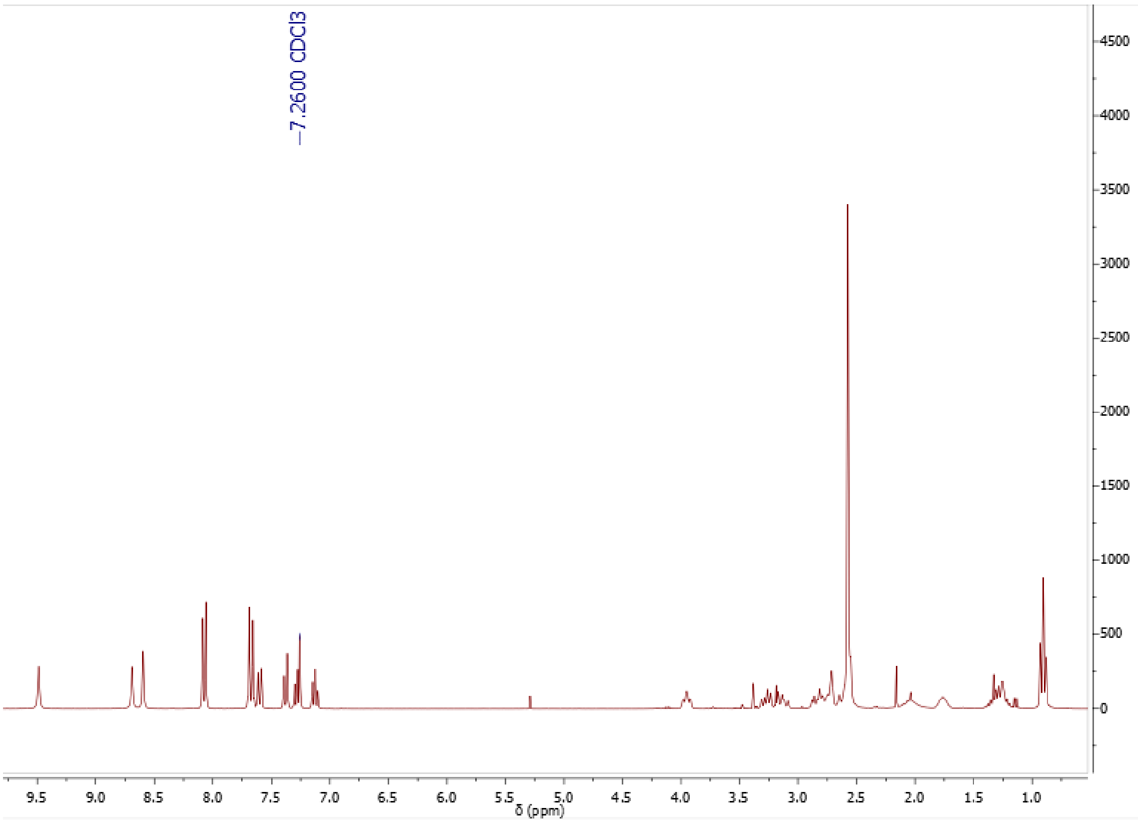

Figure S62. ^{1}H NMR spectrum of compound **30** (300 MHz, $CDCl_3$).

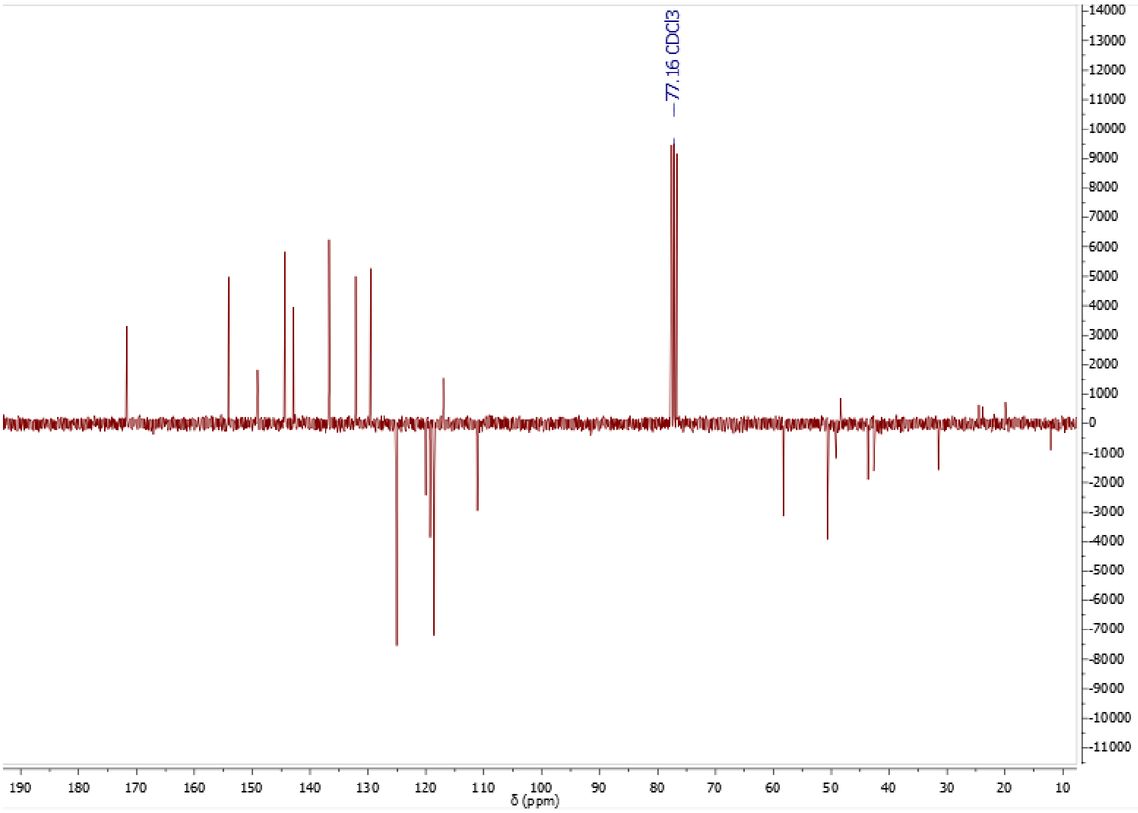

Figure S63. ^{13}C (APT) NMR spectrum of compound **30** (75 MHz, $CDCl_3$).

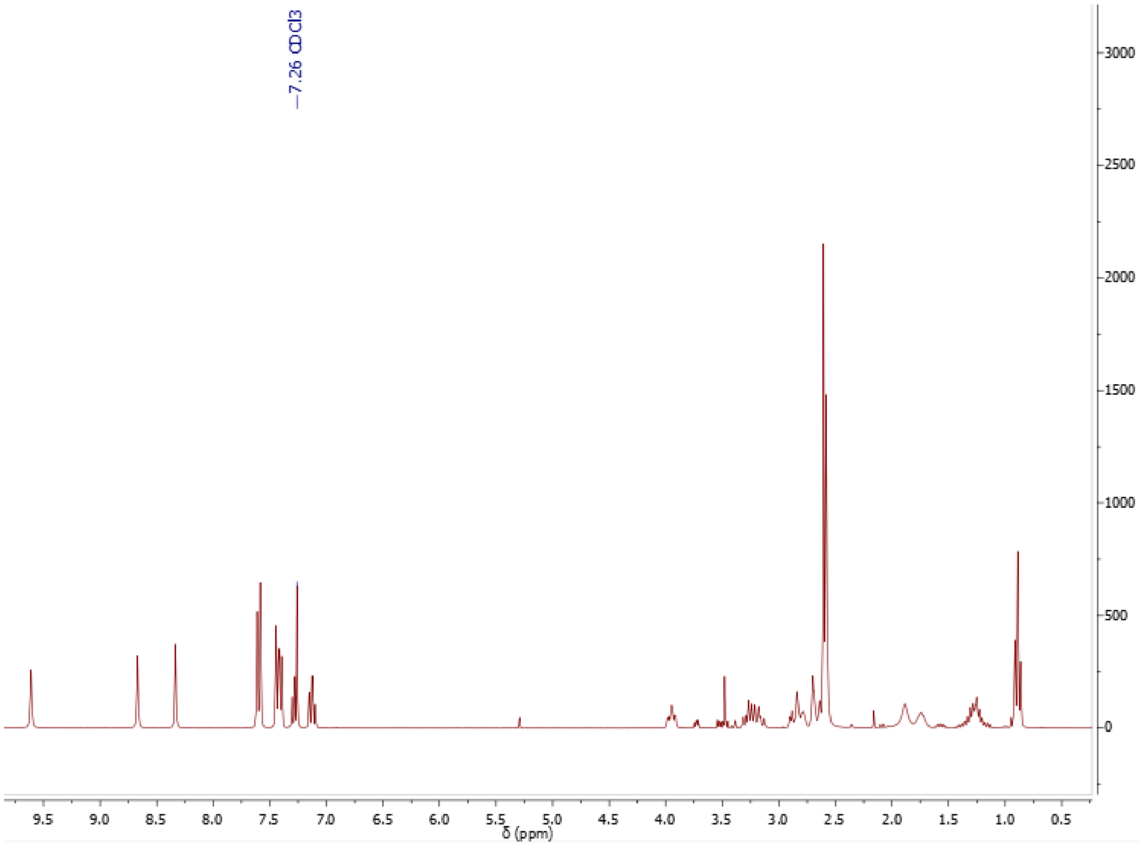

Figure S64. ^{1}H NMR spectrum of compound **31** (300 MHz, $CDCl_3$).

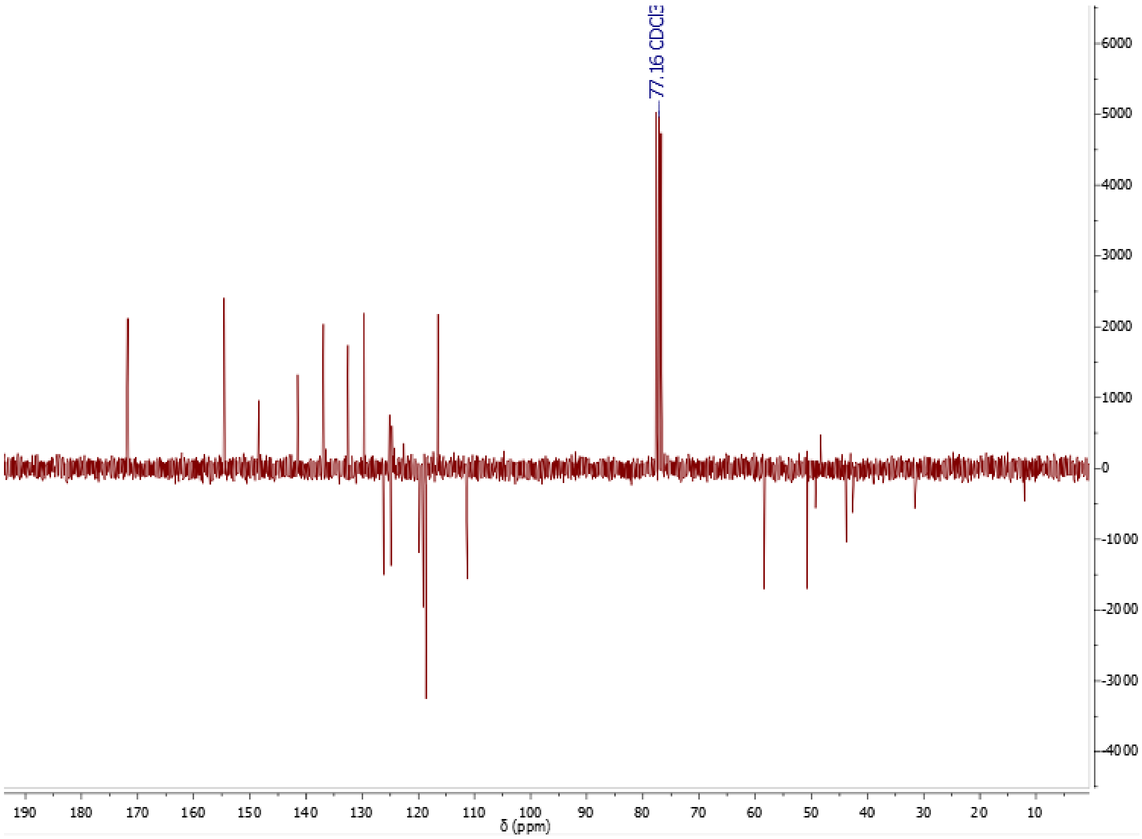

Figure S65. ^{13}C (APT) NMR spectrum of compound **31** (75 MHz, $CDCl_3$).

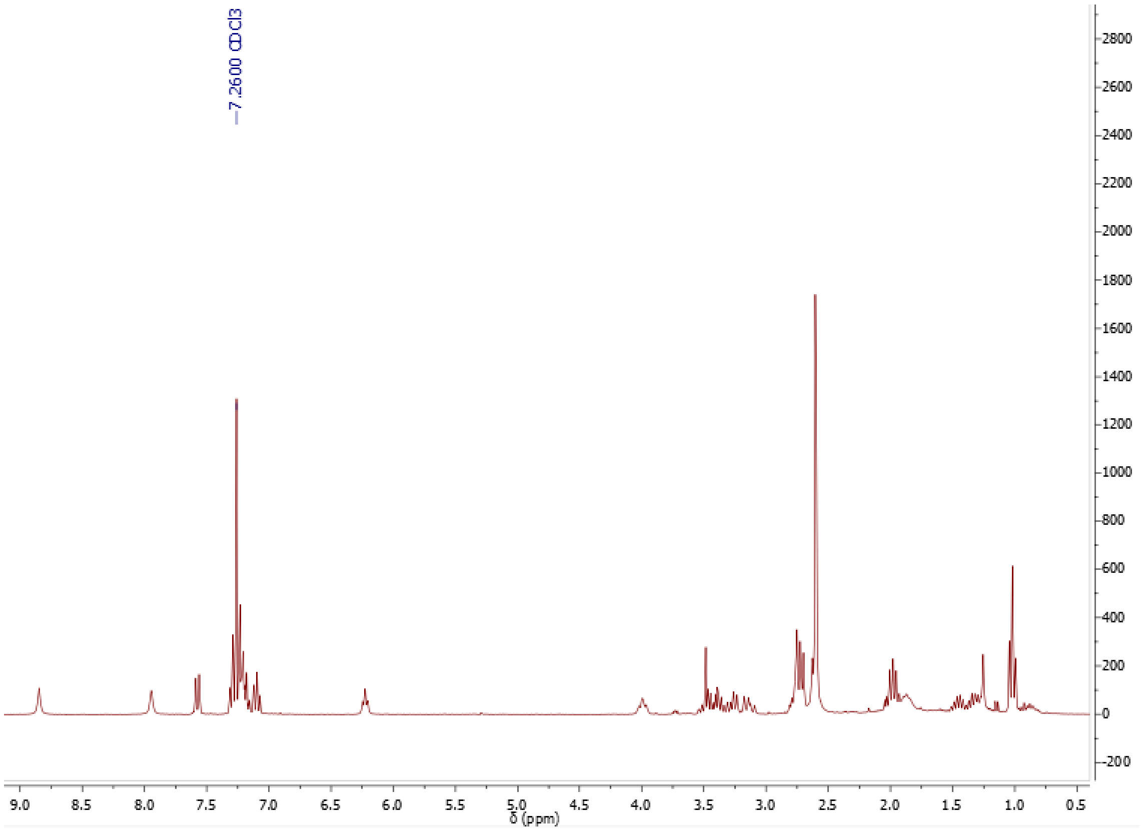

Figure S66. ^{1}H NMR spectrum of compound **32** (300 MHz, $CDCl_3$).

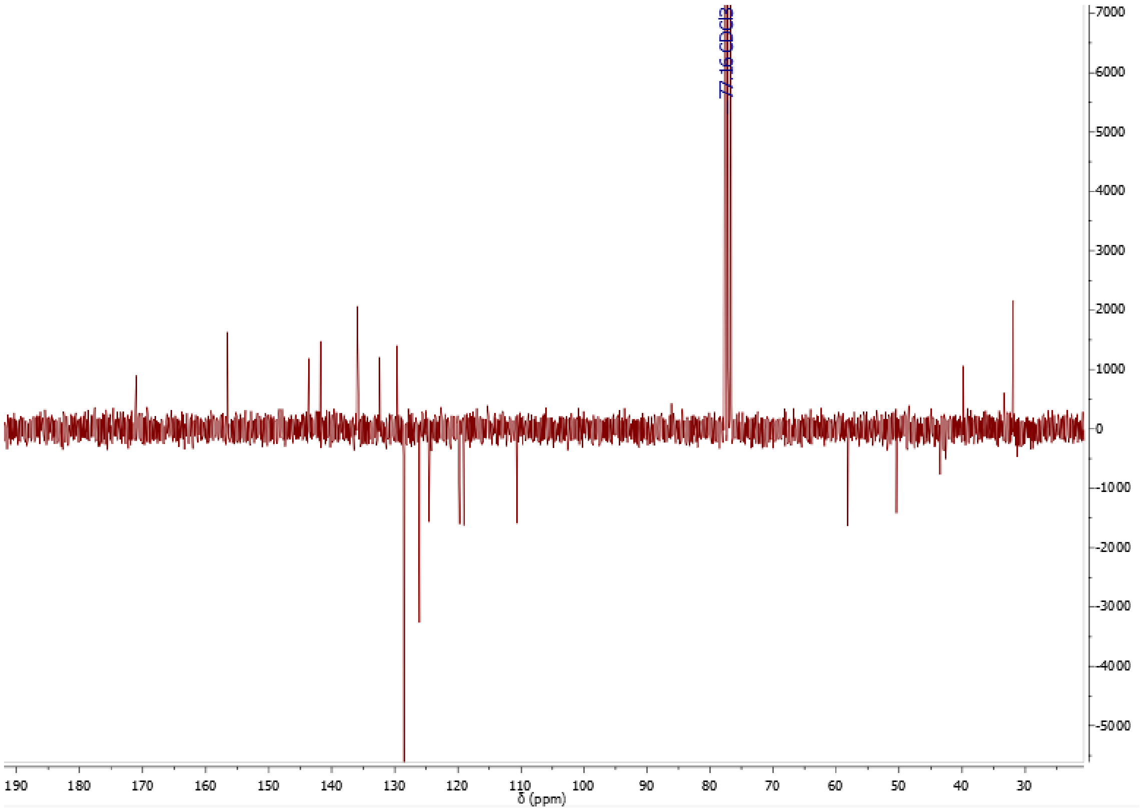

Figure S67. ^{13}C (APT) NMR spectrum of compound **32** (75 MHz, $CDCl_3$).

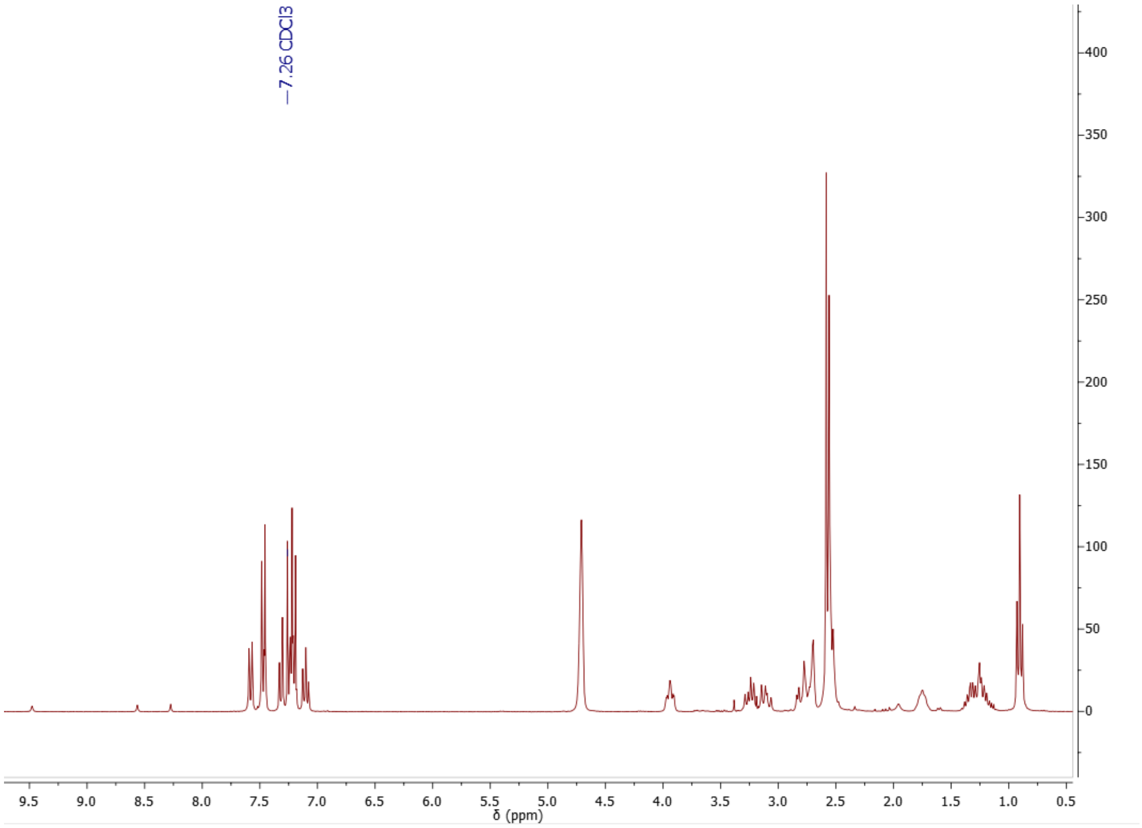

Figure S68. ^{1}H NMR spectrum of compound **28** (300 MHz, $CDCl_3 + D_2O$).

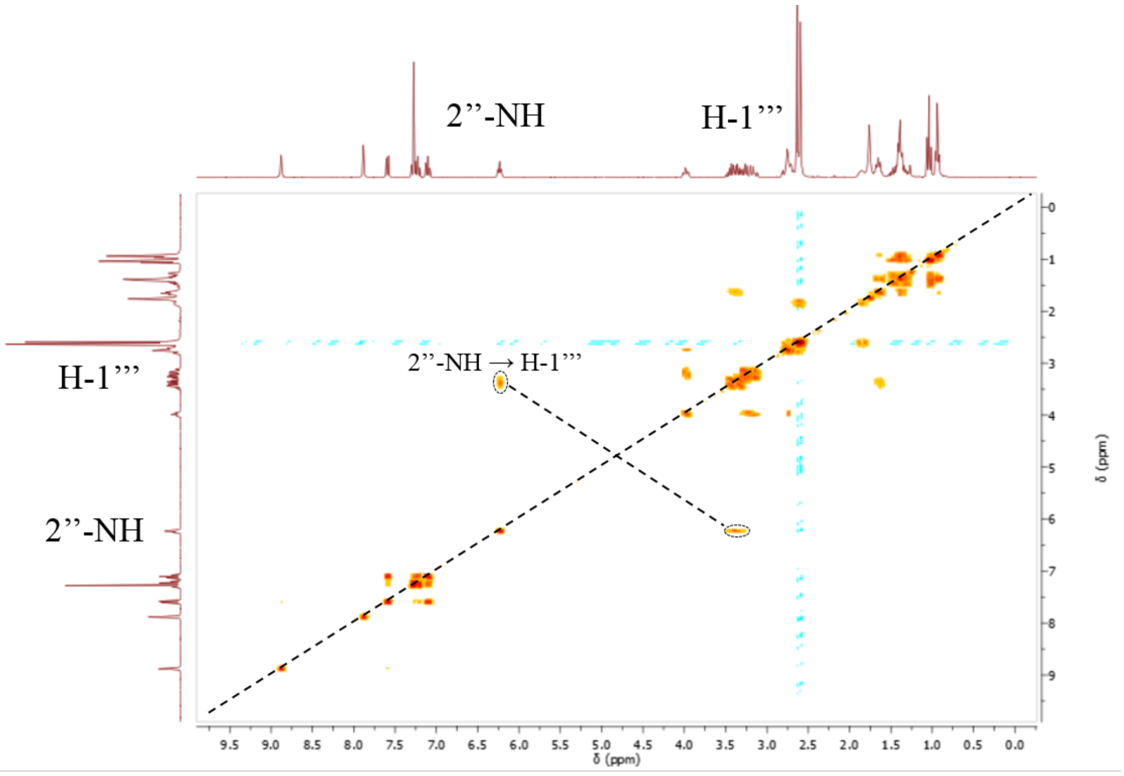

Figure S69. COSY spectrum of compound **22** (300 MHz, $CDCl_3$).

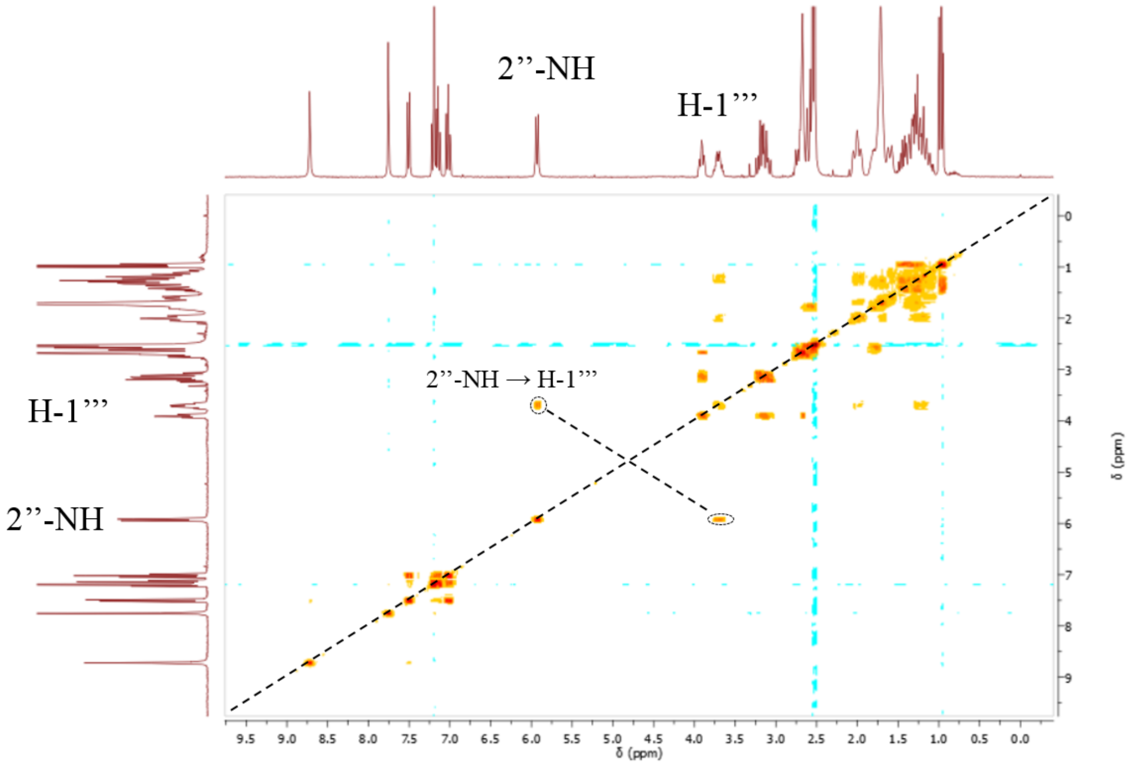

Figure S70. COSY spectrum of compound **24** (300 MHz, $CDCl_3$).

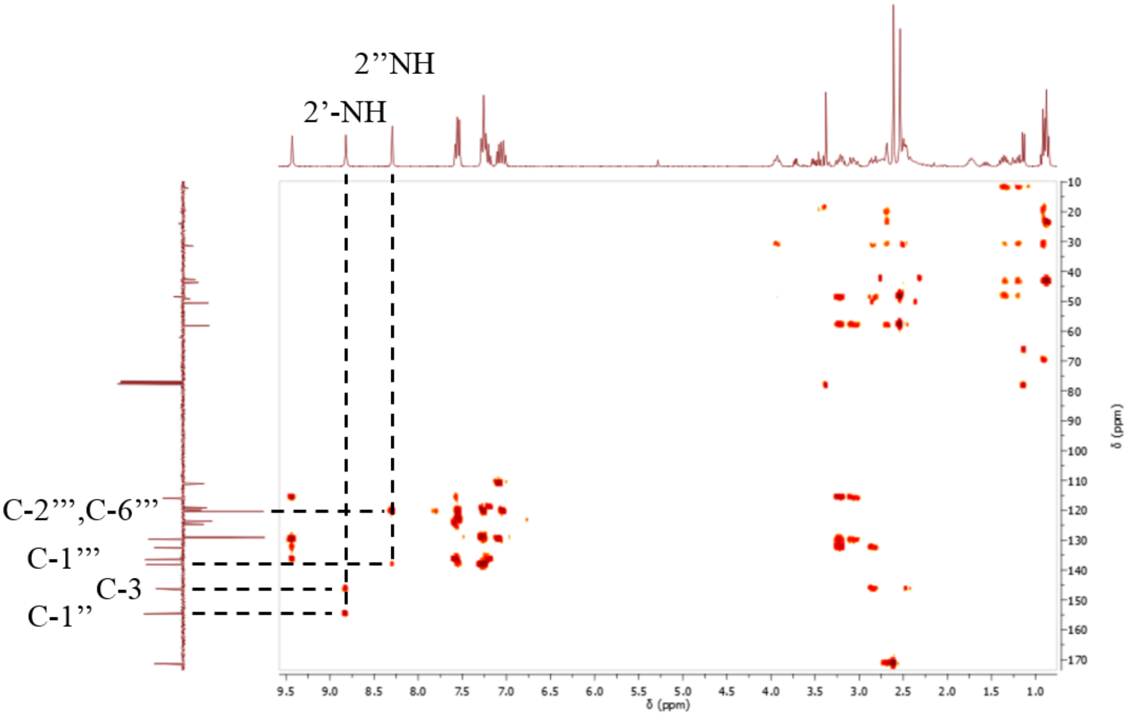

Figure S71. HMBC spectrum of compound **25** (300 MHz, $CDCl_3$).

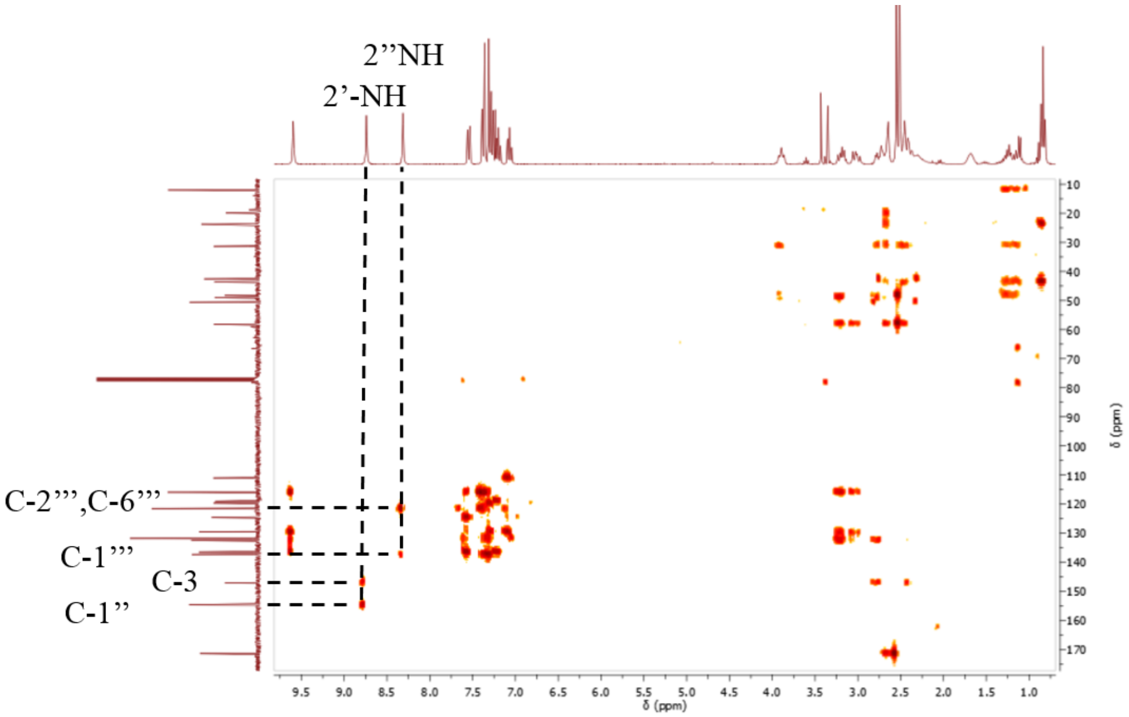

Figure S72. HMBC spectrum of compound **29** (300 MHz, $CDCl_3$).

VI) Computational Studies

Table S4. Molecular descriptors used for the univariate linear regression.

Compd.	Descriptor					FAR_{exp}
	EEig11x	X5	WTPT-2	GCUT_SLOGP_3	GCUT_SMR_3	
**1**	1.895	7.956	2.062	3.113	3.340	1.03
**3**	2.256	8.764	2.054	3.183	3.408	1.22
**4**	2.746	10.039	2.069	3.185	3.412	33.93
**5**	2.746	10.039	2.069	3.187	3.414	22.72
**6**	2.760	10.480	2.062	3.189	3.416	24.34
**7**	2.741	10.286	2.068	3.191	3.418	25.06
**8**	2.740	10.127	2.069	3.185	3.412	23.61
**9**	2.740	10.127	2.069	3.184	3.411	22.6
**10**	2.763	10.391	2.054	3.191	3.417	15.69
**11**	2.801	10.719	2.053	3.190	3.416	15.88
**12**	2.741	10.313	2.062	3.197	3.424	26.51
**13**	2.740	10.127	2.069	3.186	3.413	5.25
**14**	2.741	10.225	2.068	3.190	3.416	16.63
**15**	2.778	10.648	2.065	3.194	3.420	26.51
**16**	2.920	11.843	2.080	3.201	3.426	104.59
**17**	2.807	11.433	2.055	3.209	3.432	100.07
**18**	2.930	11.494	2.090	3.202	3.427	126.58
**19**	2.912	11.888	2.084	3.213	3.436	128.48
**20**	2.918	11.139	2.090	3.196	3.423	106.19
**21**	2.933	11.384	2.084	3.200	3.427	95.56
**22**	2.698	9.333	2.043	3.197	3.431	2.93
**23**	2.708	8.987	2.033	3.201	3.436	1.10
**24**	2.709	10.243	2.066	3.220	3.451	1.82
**25**	2.743	10.243	2.066	3.189	3.423	5.93
**26**	2.743	10.534	2.061	3.195	3.428	8.77
**27**	2.743	10.631	2.060	3.195	3.427	3.20
**28**	2.743	10.534	2.061	3.190	3.423	7.80
**29**	2.743	10.534	2.061	3.190	3.424	7.44
**30**	2.908	10.719	2.054	3.193	3.426	7.29
**31**	2.897	10.798	2.047	3.196	3.428	4.80
**32**	2.743	10.566	2.061	3.201	3.436	6.60

EEig11x: Eigenvalue 11 from edge adj. Matrix weighted by edge degrees; X5: Connectivity index of order 5; WTPT-2: Weighted path descriptor described by Randic (molecular ID/number of atoms); GCUT_SLOGP_3: Largest GCUT descriptor using atomic contribution to logP; GCUT_SMR_3: Largest GCUT descriptor using atomic contribution to molar refractivity.

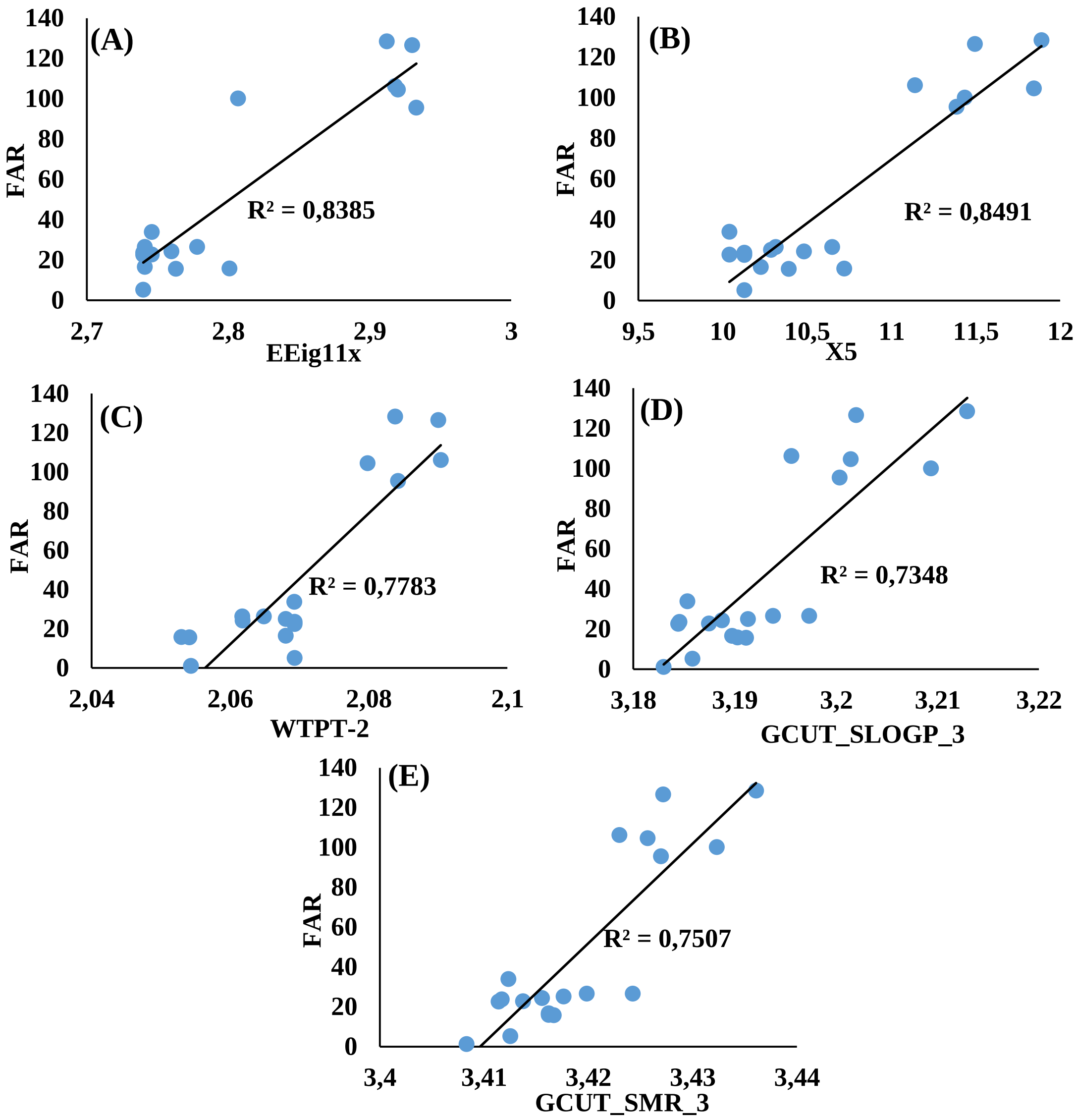

Figure S73. Representative plots of molecular descriptors (A) EEig11x, (B) X5, (C) WTPT-2, (D) GCUT_SLOGP_3 and (E) GCUT_SMR_3 *versus* experimental FAR values for the azines **3-19**. No correlation was found for semicarbazones set (**22-32**), thus were not represented.

Table S5. Molecular descriptors used for the multivariate linear regression model.

Compd.	Descriptor					FAR_{exp}	FAR_{pred}
	h_logD	vsurf_HL2	PW4	BIC3	BEHv2		
**1**	1.804	0.045	0.217	0.879	3.749	1.03	3.97
**3**	2.479	0.048	0.203	0.868	3.752	1.22	4.75
**4**	4.134	0.074	0.205	0.881	3.818	33.93	34.21
**5**	2.795	0.067	0.205	0.883	3.806	22.72	8.77
**6**	3.292	0.074	0.200	0.883	3.833	24.34	15.46
**7**	3.183	0.044	0.205	0.872	3.81	25.06	33.83
**8**	4.093	0.062	0.200	0.876	3.815	23.61	33.98
**9**	3.940	0.061	0.200	0.876	3.808	22.6	29.95
**10**	4.027	0.056	0.192	0.872	3.831	15.69	31.66
**11**	4.250	0.055	0.193	0.874	3.809	15.88	29.34
**12**	3.353	0.046	0.197	0.855	3.826	26.51	43.64
**13**	2.756	0.072	0.200	0.878	3.805	5.25	3.38
**14**	3.168	0.073	0.201	0.86	3.809	16.63	27.80
**15**	3.724	0.041	0.198	0.881	3.812	26.51	27.04
**16**	4.806	0.032	0.196	0.86	3.834	104.59	69.76
**17**	3.071	0.059	0.216	0.814	3.829	100.07	96.92
**18**	4.354	0.037	0.208	0.853	4.001	126.58	130.02
**19**	3.958	0.051	0.213	0.86	4.009	128.48	121.96
**20**	3.402	0.062	0.211	0.855	3.996	106.19	106.25
**21**	3.989	0.038	0.211	0.891	4.007	95.56	97.27
**22**	2.603	0.061	0.193	0.87	3.752	2.93	-13.50
**23**	1.881	0.073	0.194	0.84	3.752	1.1	-3.00
**24**	2.096	0.089	0.202	0.84	3.753	1.82	6.70
**25**	2.707	0.080	0.202	0.865	3.779	5.93	6.38
**26**	3.201	0.072	0.198	0.878	3.812	8.77	10.02
**27**	2.576	0.077	0.199	0.867	3.79	3.2	2.00
**28**	3.359	0.074	0.198	0.882	3.788	7.8	1.82
**29**	3.498	0.083	0.198	0.882	3.796	7.44	3.20
**30**	2.635	0.082	0.195	0.869	3.814	7.29	0.89
**31**	3.425	0.066	0.190	0.878	3.813	4.8	4.78
**32**	3.126	0.064	0.194	0.871	3.809	6.6	10.88

h_logD: Octanol/water distribution coefficient at pH 7 calculated as a state average; vsurf_HL2: Second_hydrophilic_lipophilic balance; PW4: path/walk 4 – Randic shape index; BIC3: Bond Information Content index (neighbourhood symmetry of 3-order); BEHv2: Largest eigenvalue n. 2 of Burden matrix weighted by van der Waals volume.
